# Hydrogen Bond Promoted Carbonylative Lactonization of Alkenes

**DOI:** 10.1002/chem.202500487

**Published:** 2025-03-18

**Authors:** Xin Qi, Yuanrui Wang, Xiao‐Feng Wu

**Affiliations:** ^1^ Dalian National Laboratory for Clean Energy Dalian Institute of Chemical Physics Chinese Academy of Sciences Dalian 116023, China; ^2^ University of Chinese Academy of Sciences Beijing 101408 China; ^3^ Leibniz-Institut für Katalyse e. V. Albert-Einstein-Straße 29a 18059 Rostock Germany

**Keywords:** carbonylation, cyclization, lactone, alkene, heterocycle

## Abstract

The formation of complexed molecules through intramolecular hydrogen bond to facilitate chemical reactions is an applicable strategy in organic synthesis. Among the lactones, γ‐butyrolactones is a class of biologically active and important structures. In this work, a series of γ‐butyrolactones were synthesised in a green and efficient manner by carbonylation of alkenes. This reaction is also applicable to the synthesis of lactones containing natural product modules. The formation of an intramolecular hydrogen bond between the pyridine group and the hydroxyl group is imperative for enhancing the nucleophilicity of the large steric alcohol, which are challenge to lactonize. The existence of intramolecular hydrogen bond was proven with the aid of FT‐IR and NMR characterisation.

## Graphical abstract

A new carbonylative procedure that use tertiary alcohols containing pyridine groups and an allyl group as substates to synthesize a series of lactones has been developed. The formation of an intramolecular hydrogen bond between the pyridine group and the hydroxyl group is crucial for the transformation which enhanced the nucleophilicity of the alcohol group.

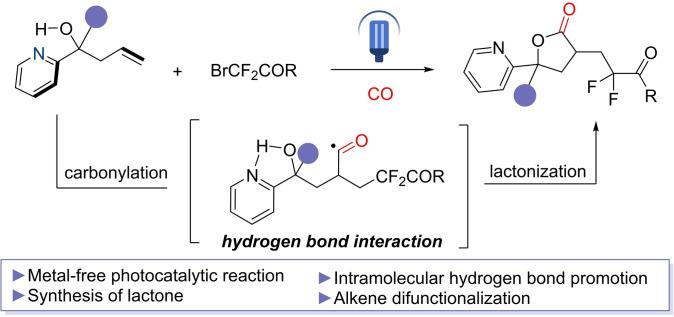

## Introduction

Hydrogen bond, a non‐chemical bond characterized by electrostatic attraction, has been identified in a variety of disciplines, including electrochemistry, biomimetic catalysis, biomedical science, materials science, and numerous others.[^1^, ^2^, ^3^, ^4^, ^5^, ^6^, ^7^] The formation of this strong electrostatic attraction is typically observed between a hydrogen atom and another atom that possesses a lone pair of electrons attached to a negatively charged atom, such as N, O, or F. This form of molecular interaction sets it apart from conventional organic transformations and molecular activation, which typically rely on catalysts, heat, light, electricity, or metals to activate molecular bonds.[^8^, ^9^, ^10^, ^11^, ^12^, ^13^, ^14^, ^15^]

**Figure 1:**
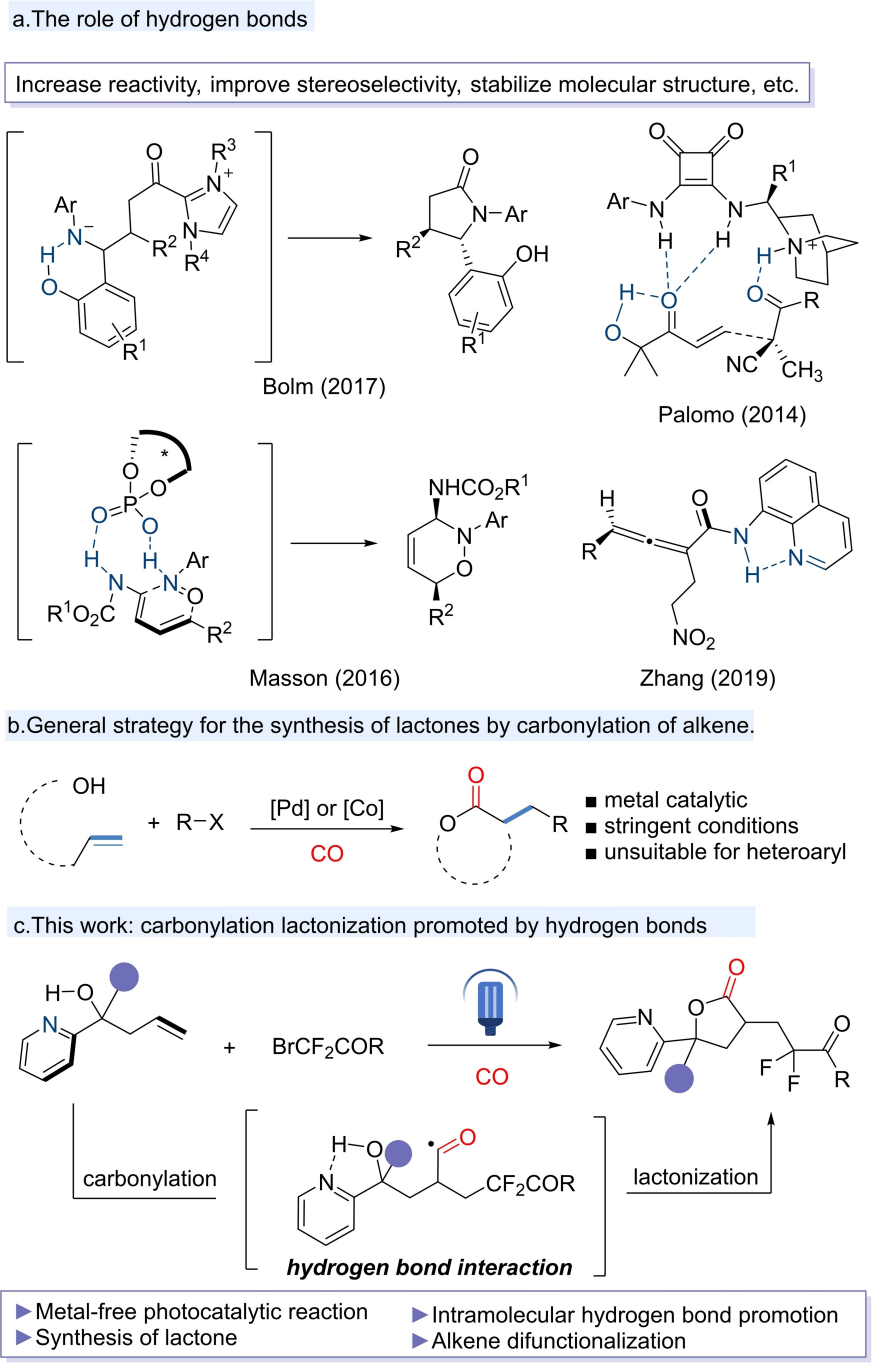
(a) The role of hydrogen bond. (b) General strategy for the synthesis of lactones by carbonylation of alkenes. (c) This work: carbonylation lactonization promoted by hydrogen bond.

Hydrogen bonds can be formed among different molecules, thereby facilitating intermolecular interactions that serve as the catalyst for chemical reactions, thus giving rise to the concept of hydrogen bonds catalysis. A number of organic catalysts based on hydrogen bonds have been developed, including TADDOL,[^13^, ^16^] guanidinium,^[17]^ phosphoric acid,^[18]^ and thiourea.^[19]^ The reported effects of hydrogen bonds include the enhancing molecular reactivity, improving stereoselectivity and stabilizing molecular structure (Figure 1a).[^20^, ^21^, ^22^, ^23^] Compared to those well‐established and utilized organic catalysts based on hydrogen bonds, fewer studies have been carried out on intramolecular hydrogen bonds promoted reactions. Furthermore, the formation of hydrogen bonds is facilitated by the presence of heteroatoms, so systems containing heteroatoms, such as heteroaryl groups, exhibit an inherent propensity for the development of hydrogen bond interactions. The presence of heteroaryls has been shown to often have deleterious effect on metal‐catalyzed coupling reactions (Figure 1b).[^24^, ^25^, ^26^] Given the above considerations, it is rational to utilize intramolecular hydrogen bond to facilitate reactions that are challenging to attain through traditional catalytic methodologies.

γ‐Butyrolactone, a five‐membered heterocyclic ring containing an ester functional group, has garnered significant attention from the scientific community due to its diverse biological and pharmacological activities.[^27^, ^28^, ^29^] The general synthesis of γ‐butyrolactones frequently necessitates the employment of strong oxidizing agents or acids, or the utilization of pre‐functionalized substrates.[^30^, ^31^, ^32^, ^33^] Consequently, the development of novel catalytic methods for the synthesis of arylated γ‐butyrolactones from simple starting materials has emerged as a prominent area of research in synthetic chemistry.

It has been established that carbonylation with CO is a highly effective method for synthesizing carbonyl‐containing compounds and their derivatives. Difunctionalization of alkenes has emerged as a particularly promising strategy, offering expeditious integration of multiple molecular backbones with a wide array of functionalities. The development of a catalytic carbonylation of alkenes is poised to become a potent synthetic strategy.[^34^, ^35^, ^36^, ^37^, ^38^] The synthesis of lactones by transition metal‐catalyzed carbonylation of alkenes has been developed (Figure 1b),[^24^, ^25^, ^26^] yet alkenes containing an electron‐deficient heteroaryl, such as pyridine, have rarely been utilized. Pyridine is of significant importance due to its role as the molecular foundation of numerous pharmaceutical agents and bioactive molecules, which are extensively utilized in the fields of pharmaceuticals and pesticides. Consequently, there is significant interest in the synthesis of lactones that incorporate pyridine moiety. The N atom in pyridine is a natural hydrogen bond acceptor. In our present design, the nitrogen atom in the 2‐pyridine group is utilized as a hydrogen bond acceptor to form intramolecular hydrogen bond with the hydroxyl group. This interaction enhances the nucleophilicity of the hydroxyl, promote the generation of lactones by intramolecular nucleophilic attract after carbonylation (Figure 1c).

## Results and Discussion

We started our investigation by using **1** **a** 1,1‐di(pyridin‐2‐yl)but‐3‐en‐1‐ol as the model substrate and ethyl bromodifluoroacetate as the coupling partner. In order to ascertain the optimal conditions, the reaction was conducted in the absence of a base, yet the yield was found to be suboptimal (Table 1, entry 1). It was determined that the addition of a base was necessary to neutralize the hydrogen bromide that might be produced by the reaction. Initially, we explored inorganic bases, such as KH_2_PO_4_ and Na_2_CO_3_, but these proved to be inadequate (Table 1, entries 2–4). Consequently, we turned to organic bases for further investigation. Organic bases, such as NEt_3_, yielded a 25 % result (Table 1, entries 5,6), falling significantly short of the desired outcome. The addition of DABCO resulted in a 50 % yield, which is considered the optimal base (Table 1, entry 7). Following the identification of the and base, a series of experiments were conducted with various solvents. It was ascertained that the reaction exhibited enhanced efficacy in the majority of non‐protonic solvents. DMF was identified as the optimal solvent (Table 1, entries 8–12). Thereafter, we explored the use of organic dyes as photocatalysts. However, only minimal product detection was observed with Rhodamine B. 4CzIPN emerged as the most effective photocatalyst here (Table 1, entries 12–15). Subsequent to the establishment of the prevailing conditions, an effort was undertaken to mitigate the pressure of CO. Upon achieving a pressure reduction to 30 bar, the yield demonstrated comparability to that attained under 40 bar conditions. As both the base and **2** **a** were increased to 2 equiv, the template reaction yielded 77 % of the desired product **3** **a** (Table 1, entry 16). The reaction could not proceed in the absence of the photocatalyst, indicating that in this reaction was not initiated by DABCO (Table 1, entry 17). Shorten the reaction time led to significantly decreased yield of the desired product (For more details of optimization, please see Supporting Information).

**Table 1:** Optimization of reaction condition.^[a]^

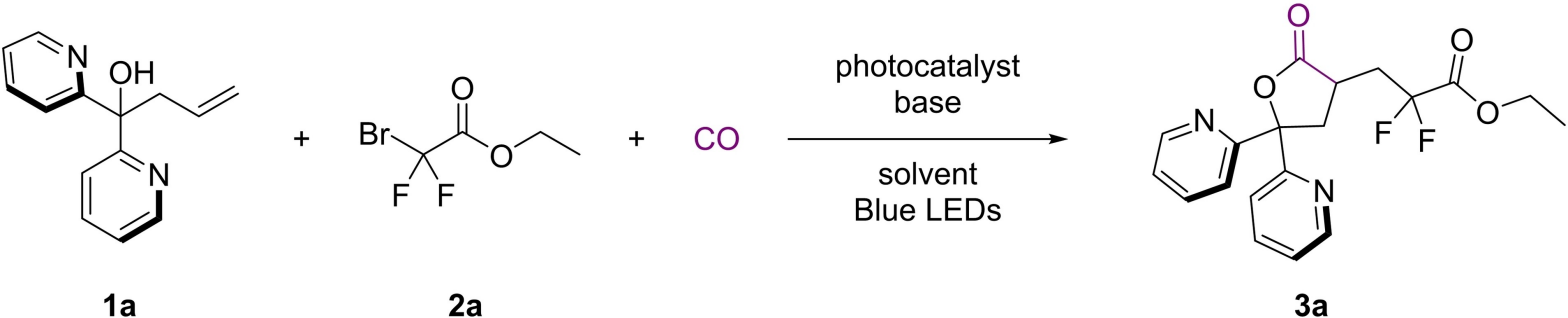				
				
Entry	Base	Catalyst	Solvent	Yield (%)^[b]^
1	no base	4CzIPN	CH_3_CN	12
2	KH_2_PO_4_	4CzIPN	CH_3_CN	trace
3	Na_2_CO_3_	4CzIPN	CH_3_CN	18
4	KO^*t*^Bu	4CzIPN	CH_3_CN	NR
5	NEt_3_	4CzIPN	CH_3_CN	25
6	DIPEA	4CzIPN	CH_3_CN	21
7	DABCO	4CzIPN	CH_3_CN	53
8	DABCO	4CzIPN	DCE	50
9	DABCO	4CzIPN	Dioxane	57
10	DABCO	4CzIPN	EA	50
11	DABCO	4CzIPN	PhCF_3_	15
12	DABCO	4CzIPN	DMF	67
13	DABCO	Rhodamine B	DMF	trace
14	DABCO	Rose bengal	DMF	NR
15	DABCO	Eosin Y	DMF	NR
16^[c]^	DABCO	4CzIPN	DMF	77 (70)
17	DABCO	no catalyst	CH_3_CN	NR
Photocatalyst:				
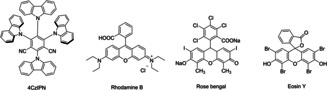				

[a] Reaction conditions: **1** **a** (0.2 mmol), **2** **a** (0.3 mmol), base (1.5 equiv.), photocatalyst (2 mol%), CO (40 bar), solvent (2.0 mL), rt, 24 h. [b] Yield was determined by GC the isolated yield is given in parentheses. NR=no reaction. [c] **2** **a** (0.4 mmol), base (2.0 equiv.), CO (30 bar), other conditions remain the same.

**Scheme 1:**
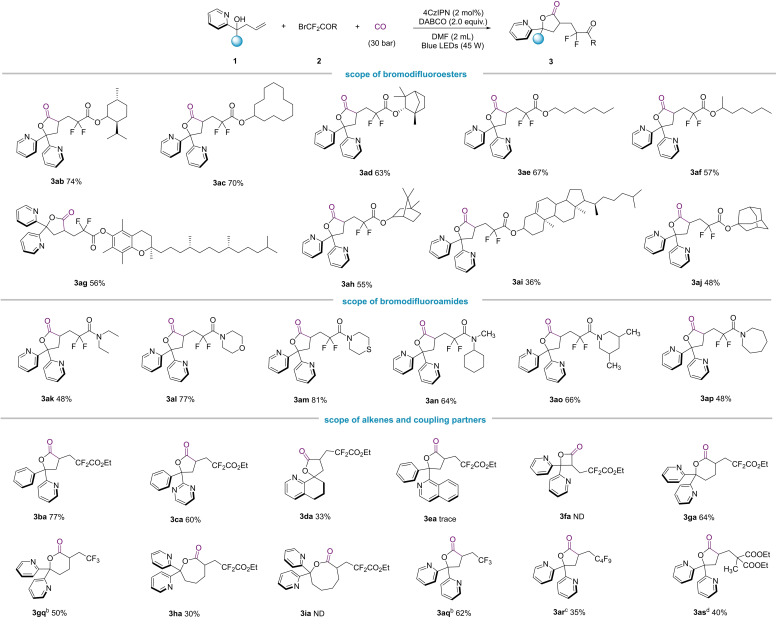
Evaluating of the substrates scope.^[a]^ Reaction conditions: (a) **1** (0.2 mmol), **2** (0.3 mmol), base (1.5 equiv.), photocatalyst (2 mol %), CO (30 bar), solvent (2.0 mL), rt, 24 h. (b) **1** (0.2 mmol), Togni's Reagent (0.3 mmol), photocatalyst (2 mol %), CO (30 bar), solvent (2.0 mL), rt, 24 h. (c) **1** (0.2 mmol), perfluorobutyl Iodide (0.3 mmol), base (1.5 equiv.), photocatalyst (2 mol %), CO (30 bar), solvent (2.0 mL), rt, 24 h. (d) **1** (0.2 mmol), diethyl 2‐bromo‐2‐methylmalonate (0.3 mmol), base (1.5 equiv.), photocatalyst (2 mol %), CO (30 bar), solvent (2.0 mL), rt, 24 h.

With the base and reaction conditions optimized, the generality of the alkene carbonylation lactonization reaction was investigated by employing various coupling partners. Various bromodifluoroesters were compatible with the standard reaction conditions. The bromodifluoroesters from long chain alkyl alcohols were all well tolerated and possessed high yields of the corresponding products (Scheme 1, **3** **ae**, **3** **af**). To demonstrate the utility of this reaction, we tried to use it to synthesize γ‐butyrolactones containing natural products such as **3** **ag** from vitamin E and **3** **ai** from cholesterol, but the yield of **3** **ai** was not high, presumably because of the poor solubility of the bromodifluorinated ester from cholesterol. Additionally, spironolactone skeletons could be synthesized by this method (Scheme 1, **3** **da**), but the yield was only 33 %, probably due to ring tension. Over and above bromodifluoroesters, bromodifluoroamides were also compatible with this reaction and gave the corresponding products in good yields (Scheme 1, **3** **ak**–**3** **ap**). In addition to the introduction of difluoromethyl group, we also tried to use perfluoroalkyl iodide as the radical precursor and resulted the targeted product in 35 % yield (Scheme 1, **3** **ar**). Non‐fluoro‐containing alkyl radical can be applied as well and 40 % of the corresponding lactone was isolated (Scheme 1, **3** **as**). In the cases for introducing trifluoromethyl using Togni's Reagent, better yields were obtained (Scheme 1, **3** **gq**, **3** **aq**). Outside of the initial five‐membered lactones, different ring size can also be produced. However, the reaction failed with seven‐membered ring and four‐membered ring systems (Scheme 1, **3** **fa**–**3** **ia**).

**Scheme 2:**
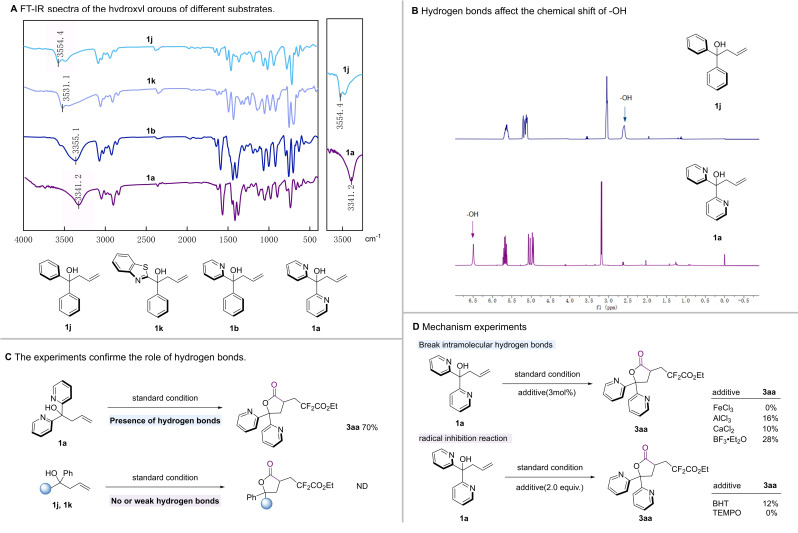
Characterization and mechanism experiments. (A) FT‐IR spectra of the hydroxyl groups of different substrates. (B) Partial NMR ^1^H of **1** **j** and **1** **a**, comparing the chemical shifts of hydroxyl affected by hydrogen bonds. (C) The experiments confirm the role of hydrogen bonds. (D) Mechanism experiments include hydrogen bond disruption experiments and radical inhibition experiments.

From mechanism point of view, the tertiary alcohol used here is characterized by a large site resistance, which results in a low propensity for lactone formation. It is hypothesized that the presence of intramolecular hydrogen bonds facilitates lactonization with **1** **a**. In order to be more explicit about the presence of intramolecular hydrogen bond in pyridine‐containing substrates, we performed infrared characterization of several substrates (Scheme 2, **A**). The general vibrational peak of the hydroxyl group is at 3600 cm^−1^, and the vibrational peak is red‐shifted after the formation of intramolecular hydrogen bonds, a phenomenon that can be well demonstrated in **A**. The hydroxyl groups were red‐shifted for substrates containing 2‐pyridine group substituents (Scheme 2, **A**, **1** **a**, **1** **b**). We have previously studied that tertiary alcohols containing benzothiazole undergo benzothiazole migration after intercalation,[^39^, ^40^, ^41^, ^42^, ^43^, ^44^] and the hydroxyl group and benzothiazole can also generate hydrogen bond force, but we did not find the formation of lactone in this experiment (Scheme 2, **C**), and we hypothesized based on the infrared results that it should be due to the fact that the strength of the hydrogen bond formed between benzothiazole and hydroxyl group is too weak and does not enhance the nucleophilicity of the hydroxyl group, and thus no lactone product is formed. We then characterized the **1** **a** and **1** **j** hydrogen bonds by NMR ^1^H (Scheme 2, **B**). And comparing to the substrate that does not contain intramolecular hydrogen bond **1** **j**, the chemical shift of hydroxyl of **1** **a** is more low‐field, and it is less polar in column chromatography separation than the general compounds containing bipyridine. These spectral characterizations indicate the presence of strong intramolecular hydrogen bond in **1** **a**.

The role of intramolecular hydrogen bond is crucial in this reaction, and the lactone products cannot be obtained from **1** **j** and **1** **k**, which do not contain intramolecular hydrogen bond (Scheme 2, **C**). The addition of a modest amount of Lewis acid (3 mol%) has been observed to disrupt the hydrogen bond. Experimental results showed that the incorporation of a minute quantity of Lewis acid, such as ferric chloride or aluminum chloride, results in a substantial decline in the yield (Scheme 2, **D**). To further substantiate the mechanisms through which the reaction occurs, radical inhibition experiments were conducted. The incorporation of a radical inhibitor led to a substantial decline in yield, thereby indicating that the reaction occurs through the radical pathway.

**Scheme 3:**
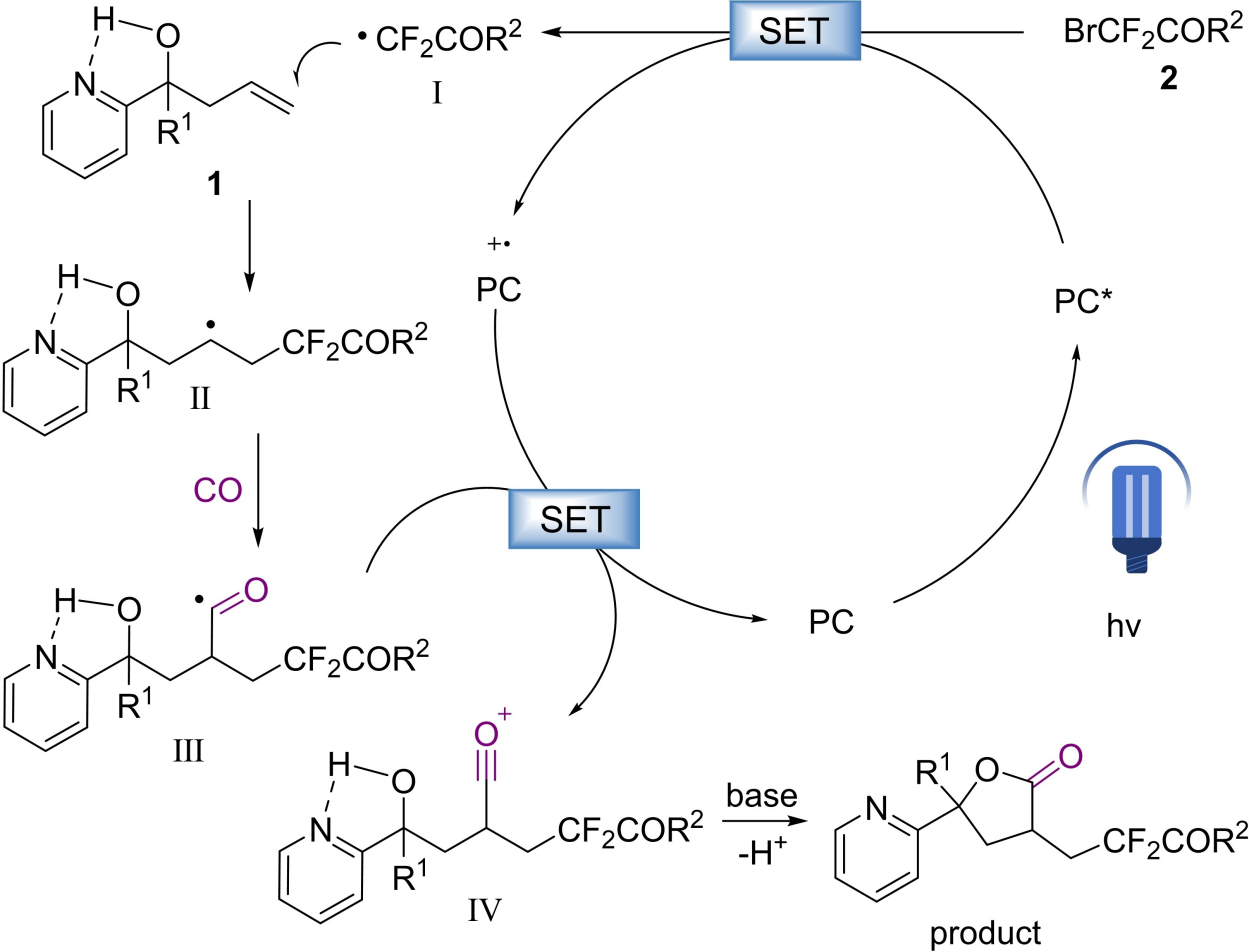
Proposed mechanism.

Combined mechanism validation experiments and our previous studies,[^39^, ^40^, ^41^, ^42^, ^44^, ^45^, ^46^] we propose a possible mechanism for this reaction (Scheme 3). First, the photocatalyst absorbs photons, entering an excited state that subsequently generates the radical intermediate I from **2** through a process of single electron transfer (SET) process. The radical intermediate I then interacts with the alkene, resulting in the formation of the radical species II, which generates the acyl radical III by capturing a molecule of CO. Compound from the hydrogen quenched radical II can be detected during the optimization process which supports the presence of intermediate II. Acyl radical III oxidized by photocatalysts to give acyl cation **IV**which subsequently deprotonate in the presence of a base and followed by intramolecular nucleophilic attack to give the final γ‐butyrolactone products. The hydrogen bond effect formed by pyridine and hydroxyl group can enhance the nucleophilicity of hydroxyl group and promote the reaction.

## Conclusions

In summary, we have developed a new carbonylative procedure that use tertiary alcohols containing pyridine groups and an allyl group as substates to synthesize a series of lactones. The tertiary alcohol, characterized by its large site resistance, usually exhibits a low propensity for lactone formation. The formation of an intramolecular hydrogen bond between the pyridine group and the hydroxyl group is crucial for the transformation which enhanced the nucleophilicity of the alcohol group. This reaction is also suitable for the synthesis of diverse lactones containing natural product modules, spironolactones, pentanolactones and hexanolactones. We demonstrated the existence of hydrogen bond with the help of FT‐IR and NMR characterization.

## Supporting Information

The authors have cited additional references within the Supporting Information.[^47^, ^48^, ^49^]

## Conflict of Interests

The authors declare no conflict of interest.

1

## Supporting information

As a service to our authors and readers, this journal provides supporting information supplied by the authors. Such materials are peer reviewed and may be re‐organized for online delivery, but are not copy‐edited or typeset. Technical support issues arising from supporting information (other than missing files) should be addressed to the authors.

Supporting Information

## Data Availability

The data that support the findings of this study are available in the supplementary material of this article.
