# Supplementary material for: Hydrogen Bond Promoted Carbonylative Lactonization of Alkenes
Source: Chemistry. 2025 Mar 18;31(22):e202500487. doi: 10.1002/chem.202500487 (PMC12015388; doi:10.1002/chem.202500487)
Supplement: Supplementary file 1 — Supporting Information [file CHEM-31-e202500487-s001.pdf]

Chemistry–A European Journal

Supporting Information

Hydrogen Bond Promoted Carbonylative Lactonization of Alkenes

Xin Qi, Yuanrui Wang, and Xiao-Feng Wu*

Supporting Information

Hydrogen Bond Promoted Carbonylative Lactonization of Alkenes

Xin Qi[ab], Yuanrui Wang[ab], Xiao-Feng Wu[abc]*

[a]Dalian National Laboratory for Clean Energy, Dalian Institute of Chemical Physics, Chinese Academy of Sciences, Dalian 116023, Liaoning, China,

[b]University of Chinese Academy of Sciences, Huairou District, Beijing, 101408, China,

[c]Leibniz-Institut für Katalyse e.V., Albert-Einstein-Straße 29a, 18059 Rostock, Germany

Table of Contents

1. General Information S2
2. Preparation of Substrates S3
3. Optimization of Reaction Conditions S6
4. Mechanistic Studies S8
5. Characterization of Substrates and Products S10
6. NMR Spectra of the Substrates and Products S24
7 Reference S80

1. General Information

Unless otherwise noted, all reactions were carried out under a carbon monoxide or nitrogen atmosphere. The reagents were ordered from Adamas-beta®, Energy Chemical Sigma-Aldrich, Bidepharm and used without purification. All solvents were dried by standard techniques and distilled prior to use. Column chromatography was performed on silica gel (200-300 meshes). All NMR spectra were recorded at ambient temperature using Bruker Avance III 400 MHz NMR (^{1}H, 400 MHz; ^{13}C{^{1}H}, 101 MHz, ^{19}F 376 MHz). ^{1}H NMR chemical shifts are reported relative to TMS and were referenced via residual proton resonances of the corresponding deuterated solvent ($CDCl_3$: 7.26 ppm) whereas ^{13}C{^{1}H} NMR spectra are reported relative to TMS via the carbon signals of the deuterated solvent ($CDCl_3$: 77.0 ppm). Data for ^{1}H are reported as follows: chemical shift (δ ppm), multiplicity (s = singlet, d = doublet, t = triplet, q = quartet, quint = quintet, m = multiplet, td = triple doublet, br = broad), coupling constant (Hz), and integration. All ^{13}C NMR spectra were broad-band ^{1}H decoupled. All reactions were monitored by GC-FID or NMR analysis. HRMS data was obtained with Micro-mass HPLC-Q-TOF mass spectrometer (ESI) or Agilent 6540 Accurate-MS spectrometer (Q-TOF). Fourier transforms infrared (FT-IR) spectra were measured in KBr media on a Bruker Tensor 2.

Because of the high toxicity of carbon monoxide, all the reactions should be performed in an autoclave. The laboratory should be well-equipped with a CO detector and alarm system.

2. Preparation of Substrates

General procedure for: 1a etc.[1]

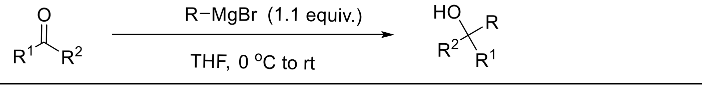

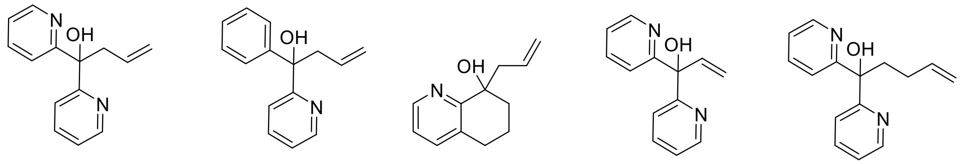

1a **1b** **1d** **1f** **1j**

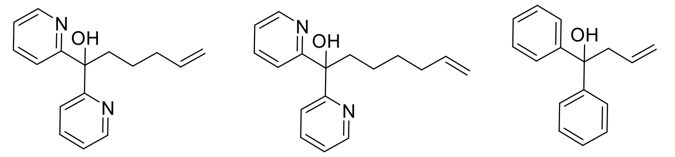

1h **1i** **1j**

To an oven-dried 25 mL double neck round bottom flask, Grignard reagent (1.1 equiv) was added dropwise to a solution of ketone 1 (1.0 equiv) in dry THF (0.2 M) under N_2 atmosphere at 0 °C. The resulting mixture was warmed gradually to room temperature and stirred for 0.5-2 h. After completion of the reaction, the reaction mixture was quenched with a saturated NH_4Cl solution, extracted with ethyl acetate and dried before purified on a silica column using an eluent of ethyl acetate/petroleum ether (1/20) to afford 1a-1j.

General procedure for: 2b etc.[2,3]

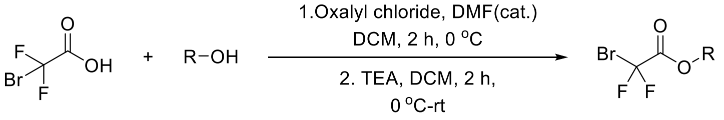

2-bromo-2,2-difluoroacetic acid (1 equiv.) was dissolved in dry DCM in a dry round bottom flask under an atmosphere of nitrogen. Oxalyl chloride (1.1 equiv.) was then added slowly into reaction system over 5 min with stirring at 20 °C. Two drops of DMF were then added, and the reaction mixture was stirred at 20 °C for further 2 h. Then a mixture of alcohol (2.0 equiv.) and Et_3N (1.1 equiv.) dissolved in dry DCM (15 mL) was added dropwise to the reaction mixture at 0 °C. The cooling was removed and stirring was continued for 6 hours. After the reaction was over, water was added to the reaction mixture and the crude was extracted with DCM (30 mL x 3). The combined organic extracts were then dried with anhydrous $MgSO_4$ and concentrated under vacuum. The residue was purified by flash column chromatography on silica gel to give the corresponding products **2b-2j.**

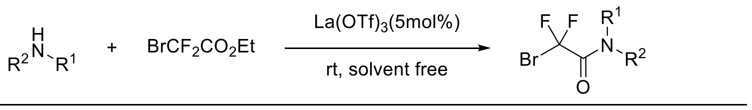

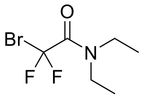

2k

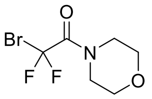

2l

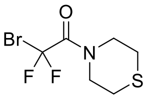

2m

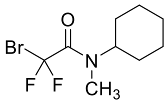

2n

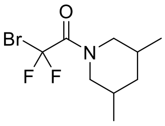

2o

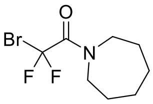

2p

A 4 mL screw-cap vial equipped with a magnetic stir bar was charged with $La(OTf)_3$ (0.1 mmol, 5 mol%). The tube was backfilled with argon, then ethyl bromodifluoroacetate **1** (2.4 mmol) and amine (2.0 mmol) were added. The mixture was stirred at the room temperature and monitored by TLC. After the amine was exhausted, purification by chromatography on silica gel afforded **2k-2p** compound is known.

General procedure for carbonylative lactonization of alkenes

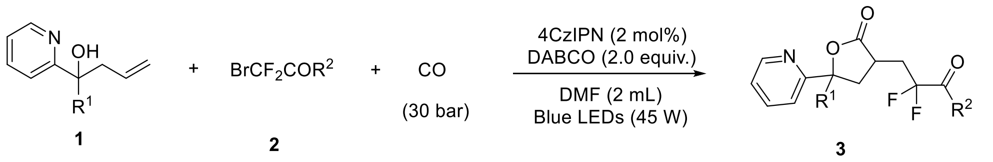

A 4 mL screw-capped vial was charged with **1** (1.0 equiv.,45 mg), 4CzIPN (2 mol%, 3.2mg), DABCO (1.5 equiv, 33.0 mg), and an oven-dried stirring bar. The vial was sealed with a Teflon septum and cap and connected to the atmosphere via a needle. After adding $BrCF_2COOR$ (**2**, 2.0 equiv., 0.3 mmol) and DMF (2.0 mL) with a syringe under nitrogen atmosphere, the vial was moved to an alloy plate and placed in a Parr 4560 series autoclave (300 mL). The autoclave was purged three times with CO and pressurized with 30 bar of CO. The autoclave was placed on a heating plate equipped with a magnetic stirrer. Reaction stirring at room temperature for 24 hours. After irradiation, the light was turned off and the pressure was released carefully. The mixture was concentrated under vacuum. The crude product was purified by column chromatography (PE/EA =5/1 to 3/1) on silica gel to afford the corresponding products.

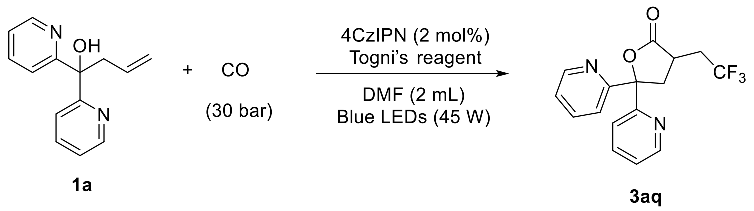

A 4 mL screw-cap vial was charged with **1a** (1.0 equiv.,45 mg, 0.2 mmol), Togni's reagent (II) (0.3 mmol), photocatalyst (2 mol%) and an oven-dried stirring bar. The vial was closed with a Teflon septum and cap and connected to the atmosphere via a needle. Then DMF (2 mL) was added with a syringe under N_2 atmosphere. The closed autoclave was flushed two times with nitrogen (~ 5 bar), and a pressure of 30 bar CO were charged. The autoclave was then placed on a magnetic stirrer. The reaction mixture was stirred while being irradiated with 45 w blue light at room temperature for 24 h. After irradiation, the light was turned off and the pressure was released carefully. The mixture was concentrated under vacuum. The crude product was purified by column chromatography (PE/EA =5/1 to 3/1) on silica gel to afford the corresponding products.

3. Optimization of Reaction Conditions

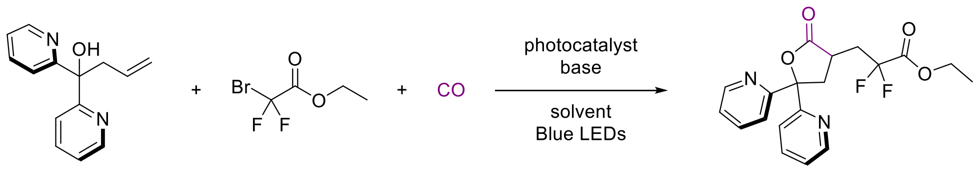

1a **2a** **3a**

Table S1. Optimization of amount of base

Entry	Base	Yield(%)
1	KH_2PO_4	trace
2	K_3PO_4	trace
3	Na_3PO_4	trace
4	$NaHCO_3$	11
5	Na_2CO_3	18
6	KO^tBu	NR
7	quinuclidine	40
8	NEt_3	25
9	DIPEA	21
10	DABCO	53
11	DBU	15
12	Without base	12

Reaction conditions: **1a** (0.2 mmol), **2a** (0.3 mmol), base (1.5 equiv.), 4CzIPN (2 mol%), CO (40 bar), MeCN (2.0 mL), rt, 24 h.

Table S2. Optimization of amount of solvent

Entry	Solvent	Yield(%)
1	DMF	67
2	DCE	50
3	Dioxane	57
4	EA	50
5	$PhCF_3$	15
6	MeCN	53
7	toluene	10
8	ethanol	NR

Reaction conditions: **1a** (0.2 mmol), **2a** (0.3 mmol), DABCO (1.5 equiv.), 4CzIPN (2 mol%), CO (40 bar), solvent (2.0 mL), rt, 24 h.

Table S3. Optimization of amount of catalyst

Entry	Catalyst	Yield(%)
1	4CzIPN	67
2	Rhodamine B	trace
3	Rose bengal	NR
4	Eosin Y	NR
5	3CzFIPN	31
6	Ir(ppy)3	52
7	Ru(bpy)3	57

Reaction conditions: **1a** (0.2 mmol), **2a** (0.3 mmol), DABCO (1.5 equiv.), photocatalyst (2 mol%), CO (40 bar), DMF (2.0 mL), rt, 24 h.

Table S4. Optimization of pressure of CO

Entry	CO	time	Yield(%)
1	30	24	77
2	20	24	62
3	10	36	50
4	1	48	24

Reaction conditions: **1a** (0.2 mmol), **2a** (0.4 mmol), DABCO (2.0 equiv.), photocatalyst (2 mol%), DMF (2.0 mL), rt.

4. Mechanistic Studies

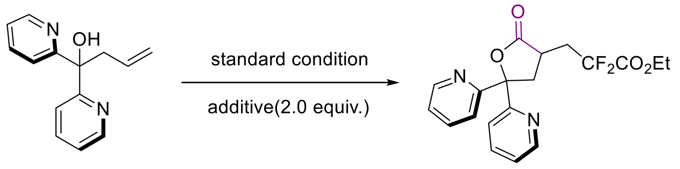

1a 3aa

additive	3aa
BHT	12%
TEMPO	0%

A 4 mL screw-capped vial was charged with **1a** (1.0 equiv.,0.2mmol, 45 mg), 4CzIPN (2 mol%, 3.2mg), DABCO (2.0 equiv, 45.0 mg), TEMPO (93.6 mg, 2.0 equiv., 0.4mmol) and an oven-dried stirring bar. The vial was sealed with a Teflon septum and cap and connected to the atmosphere via a needle. After adding $BrCF_2COOEt$ (**2a**, 2.0 equiv., 0.4 mmol) and DMF (2.0 mL) with a syringe under nitrogen atmosphere, the vial was moved to an alloy plate and placed in a Parr 4560 series autoclave (300 mL). The autoclave was purged three times with CO and pressurized with 30 bar of CO. The autoclave was placed on a heating plate equipped with a magnetic stirrer. Reaction stirring at room temperature for 24 hours. After the reaction was completed, the autoclave was cooled down with ice water to room temperature and the pressure was carefully released. After cooling to room temperature, **3aa** detected by GC-MS analysis.

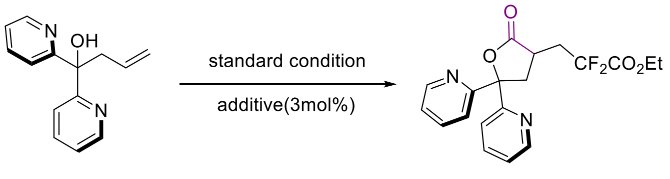

1a 3aa

additive	3aa
$FeCl_3$	0%
$AlCl_3$	16%
$CaCl_2$	10%
$BF_3 \cdot Et_2O$	28%

A 4 mL screw-capped vial was charged with **1a** (1.0 equiv., 0.2mmol, 45 mg), 4CzIPN (2 mol%, 3.2mg), DABCO (2.0 equiv, 45.0 mg), additive (3 mol%) and an oven-dried stirring bar. The vial was sealed with a Teflon septum and cap and connected to the atmosphere via a needle. After adding $BrCF_2COOEt$ (**2a**, 2.0 equiv., 0.4 mmol) and DMF (2.0 mL) with a syringe under nitrogen atmosphere, the vial was moved to an alloy plate and placed in a Parr 4560 series autoclave (300 mL). The autoclave was purged three times with CO and pressurized with 30 bar of CO. The autoclave was placed on a heating plate equipped with a magnetic stirrer. Reaction stirring at room temperature for 24 hours. After the reaction was completed, the autoclave was cooled down with ice water to room temperature and the pressure was carefully released. After cooling to room temperature, **3aa** detected by GC-MS analysis.

FT-IR spectra of the hydroxyl groups of different substrates.

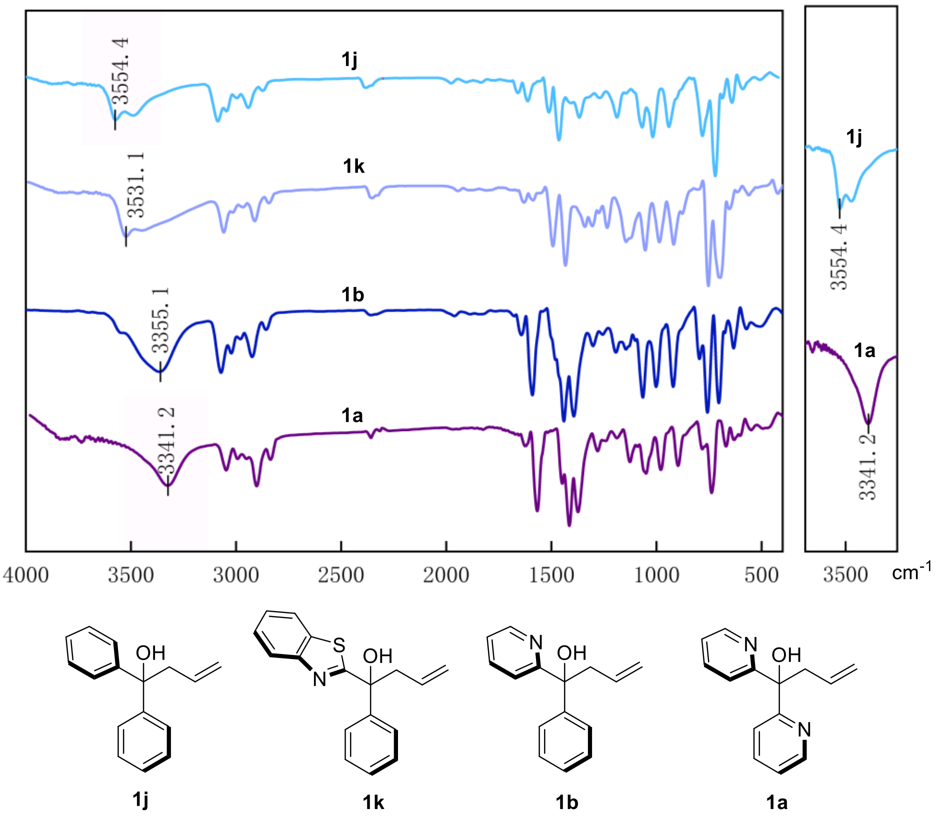

5. Characterization of Substrates and Products

Substrates

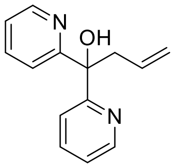

1,1-di(pyridin-2-yl)but-3-en-1-ol (1a)

^{1}H NMR (400 MHz, CDCl$_3$) δ 8.51 (d, *J* = 5.0 Hz, 2H), 7.83 (d, *J* = 8.2 Hz, 2H), 7.63 (m,2H), 7.13 (m, 2H), 6.50 (s, 1H), 5.68 (m, 1H), 5.05 (m, 1H), 5.00 – 4.92 (m, 1H), 3.18 (d, *J* = 7.0 Hz, 2H).

^{13}C NMR (101 MHz, CDCl$_3$) δ 162.9, 147.5, 136.6, 133.6, 133.5, 121.9, 121.0, 118.0, 77.8, 47.0.

HRMS (ESI-TOF) m/z: [M + H]$^+$ calculated for $C_{14}H_{14}N_2O$ 227.1179; Found 227.1180.

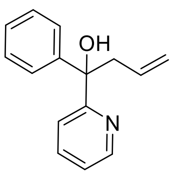

1-phenyl-1-(pyridin-2-yl)but-3-en-1-ol (1b)

^{1}H NMR (400 MHz, CDCl$_3$) δ 8.71 (d, *J* = 4.9 Hz, 2H), 7.74 (d, *J* = 7.1 Hz, 2H), 7.31 (dd, *J* = 8.5, 7.0 Hz, 2H), 7.24 – 7.18 (m, 1H), 7.15 (t, *J* = 4.9 Hz, 1H), 5.72 (m, 1H), 5.45 (s, 1H), 5.07 (d, *J* = 17.1 Hz, 1H), 4.97 (d, *J* = 10.2 Hz, 1H), 3.25 (dd, *J* = 14.0, 7.3 Hz, 1H), 3.13 (dd, *J* = 14.0, 6.6 Hz, 1H).

^{13}C NMR (101 MHz, CDCl$_3$) δ 171.7, 156.7, 145.0, 133.6, 128.0, 127.0, 125.9, 112.0, 118.2, 78.0, 45.8.

HRMS (ESI-TOF) m/z: [M + H]$^+$ calculated for $C_{15}H_{15}NO$ 226.1226; Found 226.1218.

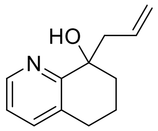

8-allyl-5,6,7,8-tetrahydroquinolin-8-ol (1d)

^{1}H NMR (400 MHz, CDCl$_3$) δ 8.42 (d, *J* = 3.2 Hz, 1H), 7.40 (d, *J* = 7.7 Hz, 1H), 7.11 (dd, *J* = 7.7, 4.7 Hz, 1H), 5.87 (m, 1H), 5.15 – 5.04 (m, 2H), 3.59 (s, 1H), 2.80 (d, *J* = 5.6 Hz, 2H), 2.68 – 2.49 (m, 2H), 2.21 – 2.10 (m, 1H), 1.95 – 1.81 (m, 3H).

^{13}C NMR (101 MHz, CDCl$_3$) δ 160.2, 146.9, 136.9, 134.1, 131.2, 122.3, 118.0, 72.1, 46.6, 33.2, 28.5, 18.9.

HRMS (ESI-TOF) m/z: [M + H]$^+$ calculated for $C_{12}H_{15}NO$ 190.1126; Found 190.1121

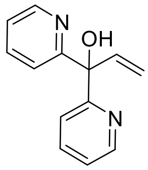

1,1-di(pyridin-2-yl)prop-2-en-1-ol (1f)
^{1}H NMR (400 MHz, CDCl$_3$) δ 8.53 (d, *J* = 4.8 Hz, 2H), 7.78 (d, *J* = 8.1 Hz, 2H), 7.66 (m, 2H), 7.20 – 7.12 (m, 2H), 6.89 (dd, *J* = 17.1, 10.5 Hz, 1H), 6.56 (s, 1H), 5.50 (dd, *J* = 17.1, 1.6 Hz, 1H), 5.25 (dd, *J* = 10.5, 1.6 Hz, 1H).
^{13}C NMR (101 MHz, CDCl$_3$) δ 162.6, 147.6, 142.4, 136.8, 122.2, 121.4, 113.7, 78.2.
HRMS (ESI-TOF) m/z: [M + H]$^+$ calculated for $C_{13}H_{12}N_2O$ 213.1022; Found 213.1033

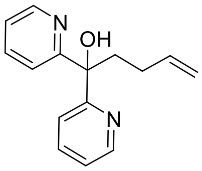

1,1-di(pyridin-2-yl)pent-4-en-1-ol (1g)
^{1}H NMR (400 MHz, CDCl$_3$) δ 8.51 (d, *J* = 4.9 Hz, 2H), 7.84 (d, *J* = 8.0 Hz, 2H), 7.64 (m, 2H), 7.13 (m, 2H), 6.53 (s, 1H), 5.80 (m, 1H), 4.94 (d, *J* = 16.8 Hz, 1H), 4.86 (d, *J* = 10.2 Hz, 1H), 2.51 – 2.43 (m, 2H), 2.06 – 1.93 (m, 2H).
^{13}C NMR (101 MHz, CDCl$_3$) δ 163.3, 147.5, 138.7, 136.7, 121.9, 121.0, 114.1, 77.9, 41.0, 28.1.
HRMS (ESI-TOF) m/z: [M + H]$^+$ calculated for $C_{15}H_{16}N_2O$ 241.1355; Found 241.1358.

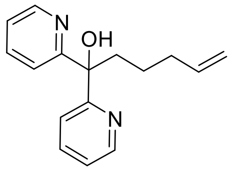

1,1-di(pyridin-2-yl)hex-5-en-1-ol (1h)
^{1}H NMR (400 MHz, CDCl$_3$) δ 8.50 (d, *J* = 4.8 Hz, 2H), 7.84 (d, *J* = 8.1 Hz, 2H), 7.62 (m, 2H), 7.11 (m, 2H), 6.51 (s, 1H), 5.75 (m, 1H), 4.94 (d, *J* = 17.2 Hz, 1H), 4.88 (d, *J* = 10.1 Hz, 1H), 2.43 – 2.34 (m, 2H), 2.03 (q, *J* = 7.2 Hz, 2H), 1.39 – 1.24 (m, 2H).
^{13}C NMR (101 MHz, CDCl$_3$) δ 163.5, 147.4, 138.8, 136.6, 121.8, 120.9, 114.3, 78.1, 41.3, 33.9, 22.9.
HRMS (ESI-TOF) m/z: [M + H]$^+$ calculated for $C_{16}H_{18}N_2O$ 255.1492; Found 255.1516.

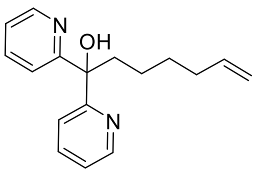

1,1-di(pyridin-2-yl)hept-6-en-1-ol (1i)

^{1}H NMR (400 MHz, CDCl$_3$) δ 8.51 (d, *J* = 4.8 Hz, 2H), 7.83 (d, *J* = 8.1 Hz, 2H), 7.63 (m, 2H), 7.16 – 7.09 (m, 2H), 6.49 (s, 1H), 5.74 (m, 1H), 5.04 – 4.58 (m, 2H), 2.42 – 2.32 (m, 2H), 2.04 – 1.89 (m, 2H), 1.43 – 1.30 (m, 2H), 1.29 – 1.17 (m, 2H).

^{13}C NMR (101 MHz, CDCl$_3$) δ 163.6, 147.4, 147.3, 139.0, 136.6, 136.6, 121.9, 121.1, 121.0, 114.0, 78.1, 47.5, 41.6, 33.8, 33.7, 29.2, 24.8, 23.1.

HRMS (ESI-TOF) m/z: $[M + H]^+$ calculated for $C_{17}H_{20}N_2O$ 269.1648; Found 269.1660.

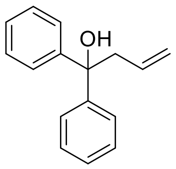

1,1-diphenylbut-3-en-1-ol (1j)

^{1}H NMR (400 MHz, CDCl$_3$) δ 7.42 (d, *J* = 9.4 Hz, 4H), 7.32 – 7.22 (m, 4H), 7.18 (d, *J* = 8.0 Hz, 2H), 5.72 – 5.56 (m, 1H), 5.19 (d, *J* = 16.9 Hz, 1H), 5.12 (d, *J* = 10.0 Hz, 1H), 3.04 (d, *J* = 8.6 Hz, 2H), 2.60 (s, 1H).

^{13}C NMR (101 MHz, CDCl$_3$) δ 146.5, 133.4, 128.1, 126.7, 125.9, 120.2, 76.8, 46.6.

HRMS (ESI-TOF) m/z: $[M + H]^+$ calculated for $C_{16}H_{16}O$ 225.1274; Found 225.1299.

Products

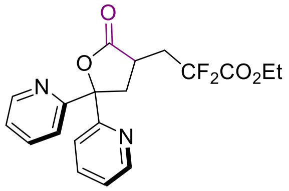

ethyl 2,2-difluoro-3-(2-oxo-5,5-di(pyridin-2-yl)tetrahydrofuran-3-yl)propanoate (3aa)

Yellow oil, 52.6 mg, 70% yield, R_f = 0.3 (PE/EA = 3/1, 1% NEt_3)

^{1}H NMR (400 MHz, $CDCl_3$) δ 8.63 (s, 2H), 7.74 – 7.63 (m, 2H), 7.48 (d, *J* = 7.9 Hz, 1H), 7.41 (d, *J* = 7.9 Hz, 1H), 7.30 – 7.19 (m, 2H), 4.33 (q, *J* = 7.1 Hz, 2H), 3.87 (dd, *J* = 13.1, 8.7 Hz, 1H), 3.10 (q, *J* = 10.4 Hz, 1H), 2.94 – 2.74 (m, 2H), 2.34 – 2.15 (m, 1H), 1.34 (t, *J* = 7.1 Hz, 3H).

^{13}C NMR (101 MHz, $CDCl_3$) δ 176.7, 163.5 (t, *J* = 32.3 Hz), 160.0, 159.4, 149.4, 149.3, 136.9, 123.1, 123.0, 120.9, 120.5, 115.0 (t, *J* = 251.1 Hz), 88.7, 63.1, 39.7, 35.5 (t, *J* = 23.7 Hz), 34.8 (t, *J* = 3.5 Hz), 13.8.

^{19}F NMR (376 MHz, $CDCl_3$) δ -104.11 (d, *J* = 261.7 Hz), -106.75 (d, *J* = 261.8 Hz).

HRMS (ESI-TOF) m/z: $[M + H]^+$ calculated for $C_{19}H_{18}F_2N_2O_4$ 377.1307; Found 377.1296.

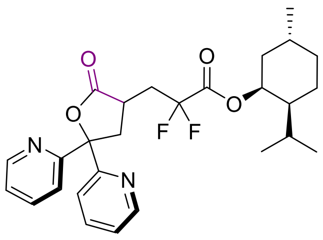

(1S,2S,5R)-2-isopropyl-5-methylcyclohexyl 2,2-difluoro-3-(2-oxo-5,5-di(pyridin-2-yl) tetrahydrofuran-3-yl)propanoate (3ab)

Colorless oil, 72.0 mg, 74% yield, R_f = 0.3 (PE/EA = 3/1, 1% NEt_3)

^{1}H NMR (400 MHz, $CDCl_3$) δ 8.64 (s, 2H), 7.68 (m, 2H), 7.48 (d, *J* = 8.1 Hz, 1H), 7.41 (d, *J* = 7.9 Hz, 1H), 7.23 (dd, *J* = 7.6, 4.8 Hz, 2H), 4.81 (m, 1H), 3.93 – 3.82 (m, 1H), 3.18 – 3.02 (m, 1H), 2.94 – 2.74 (m, 2H), 2.32 – 2.12 (m, 1H), 1.99 (d, *J* = 8.6 Hz, 1H), 1.82 (d, *J* = 7.1 Hz, 1H), 1.70 (d, *J* = 10.1 Hz, 2H), 1.48 (d, *J* = 8.8 Hz, 2H), 1.12 – 0.98 (m, 2H), 0.90 (m, 7H), 0.73 (d, *J* = 7.0 Hz, 3H).

^{13}C NMR (101 MHz, $CDCl_3$) δ 176.8, 176.7, 163.3(t, *J* = 33.0 Hz), 160.1 159.46, 149.4, 149.3, 136.9, 136.87, 123.1, 123.0, 120.9, 120.6, 88.8, 77.8, 46.7, 40.2, 40.16, 39.9, 35.5(t, *J* = 22.0 Hz), 34.8(t, *J* = 9.7 Hz), 33.9, 31.4, 26.14, 23.3, 21.9, 20.6, 16.0.

^{19}F NMR (376 MHz, $CDCl_3$) δ -103.78 (dd, *J* = 260.9, 21.3 Hz), -106.96 (dd, *J* = 260.9, 60.5 Hz).

HRMS (ESI-TOF) m/z: $[M + H]^+$ calculated for $C_{27}H_{32}F_2N_2O_4$ 487.2403; Found 487.2379.

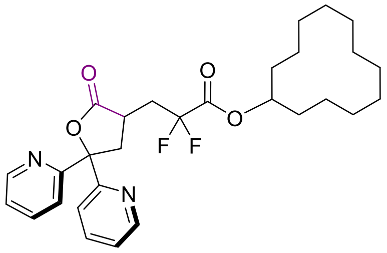

cyclododecyl 2,2-difluoro-3-(2-oxo-5,5-di(pyridin-2-yl)tetrahydrofuran-3-yl)propanoate (3ac)

Colorless oil, 72.1 mg, 70% yield, R_f = 0.3 (PE/EA = 3/1, 1% NEt_3)

^{1}H NMR (400 MHz, $CDCl_3$) δ 8.63 (d, *J* = 4.7 Hz, 2H), 7.68 (q, *J* = 7.5 Hz, 2H), 7.48 (d, *J* = 7.9 Hz, 1H), 7.41 (s, 1H), 7.26 – 7.19 (m, 2H), 5.13 (t, *J* = 4.4 Hz, 1H), 3.87 (dd, *J* = 13.2, 8.6 Hz, 1H), 3.16 – 3.04 (m, 1H), 2.84 – 2.74 (m, 2H), 2.22 (m, 1H), 1.82 – 1.72 (m, 2H), 1.56 (d, *J* = 7.1 Hz, 2H), 1.35 (d, *J* = 13.8 Hz, 18H).

^{13}C NMR (101 MHz, $CDCl_3$) δ 176.7, 163.1 (t, *J* = 32.4 Hz), 160.0, 159.4, 149.3, 149.3, 136.9, 136.8, 123.1, 123.0, 120.9, 120.5, 115.1 (t, *J* = 251.5 Hz), 88.7, 76.2, 39.8, 35.5(t, *J* = 23.8 Hz), 34.8, 28.6 (t, *J* = 5.5 Hz), 24.0, 23.8, 23.1, 20.6 .

^{19}F NMR (376 MHz, $CDCl_3$) δ -103.32 – -107.23. (m)

HRMS (ESI-TOF) m/z: $[M + H]^+$ calculated for $C_{29}H_{36}F_2N_2O_4$ 515.2716; Found 515.2717.

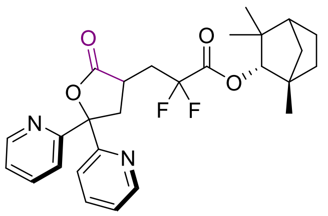

(1S,2S)-1,3,3-trimethylbicyclo[2.2.1]heptan-2-yl 2,2-difluoro-3-(2-oxo-5,5-di(pyridin-2-yl) tetrahydrofuran-3-yl)propanoate (3ad)

Colorless oil, 61.1 mg, 63% yield, R_f = 0.3 (PE/EA = 3/1, 1% NEt_3)

^{1}H NMR (400 MHz, $CDCl_3$) δ 8.63 (s, 2H), 7.68 (q, *J* = 8.3 Hz, 2H), 7.49 (d, *J* = 7.9 Hz, 1H), 7.40 (d, *J* = 6.3 Hz, 1H), 7.27 – 7.19 (m, 2H), 4.47 (s, 1H), 3.88 (dd, *J* = 13.2, 8.7 Hz, 1H), 3.20 – 3.07 (m, 1H), 2.97 – 2.76 (m, 2H), 2.33 – 2.14 (m, 1H), 1.79 – 1.67 (m, 3H), 1.59 (d, *J* = 10.6 Hz, 1H), 1.46 (d, *J* = 16.2 Hz, 1H), 1.26 – 1.21 (m, 1H), 1.11 (m, 4H), 1.03 (d, *J* = 13.9 Hz, 3H), 0.77 (d, *J* = 5.1 Hz, 3H).

^{13}C NMR (101 MHz, $CDCl_3$) δ 176.7, 163.9 (t, *J* = 33.3 Hz), 160.0, 159.4, 149.4, 149.3, 136.9, 123.1, 123.0, 120.9, 120.8, 120.5, 115.3 (t, *J* = 252.5 Hz), 89.3, 88.7, 48.4, 48.1, 41.2, 39.6, 39.0, 35.6 (t, *J* = 23.2 Hz), 34.83 (t, *J* = 3.5 Hz), 29.5, 26.4, 25.6, 19.9, 19.1.

^{19}F NMR (376 MHz, $CDCl_3$) δ -102.73 (dd, *J* = 261.8, 55.2 Hz), -106.85 (dd, *J* = 261.8, 13.5 Hz).

HRMS (ESI-TOF) m/z: $[M + H]^+$ calculated for $C_{27}H_{30}F_2N_2O_4$ 485.2246; Found 485.2249.

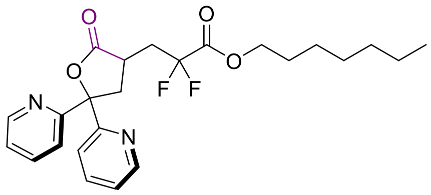

heptyl 2,2-difluoro-3-(2-oxo-5,5-di(pyridin-2-yl)tetrahydrofuran-3-yl)propanoate (3ae)
Colorless oil, 60.0 mg, 67% yield, R_f = 0.3 (PE/EA = 3/1, 1% NEt_3)
^{1}H NMR (400 MHz, $CDCl_3$) δ 8.63 (s, 2H), 7.74 – 7.63 (m, 2H), 7.48 (d, *J* = 8.0 Hz, 1H), 7.41 (d, *J* = 8.0 Hz, 1H), 7.27 – 7.19 (m, 2H), 4.26 (t, *J* = 6.7 Hz, 2H), 3.87 (dd, *J* = 13.2, 8.7 Hz, 1H), 3.17 – 3.04 (m, 1H), 2.94 – 2.74 (m, 2H), 2.25 (m, 1H), 1.69 (t, *J* = 7.0 Hz, 2H), 1.31 (d, *J* = 19.1 Hz, 8H), 0.88 (t, *J* = 6.6 Hz, 3H).
^{13}C NMR (101 MHz, $CDCl_3$) δ 176.7, 163.6(t, *J* = 32.3 Hz), 160.1, 159.4, 149.4, 149.3, 136.9, 136.87, 123.1, 123.0, 120.9, 120.5, 115.1(t, *J* = 251.2 Hz), 88.7, 67.2, 39.7, 35.6(t, *J* = 23.7 Hz), 34.8(t, *J* = 3.5 Hz), 31.5, 28.7, 3.18, 25.5, 22.5, 14.0.
^{19}F NMR (376 MHz, $CDCl_3$) δ -104.00 (d, *J* = 261.7 Hz), -106.69 (d, *J* = 261.7 Hz).
HRMS (ESI-TOF) m/z: $[M + H]^+$ calculated for $C_{27}H_{30}F_2N_2O_4$ 447.2090; Found 447.2185.

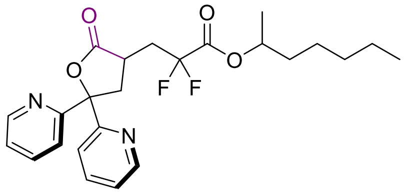

heptan-2-yl 2,2-difluoro-3-(2-oxo-5,5-di(pyridin-2-yl)tetrahydrofuran-3-yl)propanoate (3af)
Colorless oil, 51.0 mg, 57% yield, R_f = 0.3 (PE/EA = 3/1, 1% NEt_3)
^{1}H NMR (400 MHz, $CDCl_3$) δ 8.63 (d, *J* = 5.2 Hz, 2H), 7.68 (q, *J* = 7.7 Hz, 2H), 7.48 (d, *J* = 7.9 Hz, 1H), 7.41 (d, *J* = 8.0 Hz, 1H), 7.26 – 7.19 (m, 2H), 5.03 (q, *J* = 6.4 Hz, 1H), 3.88 (dd, *J* = 13.2, 8.6 Hz, 1H), 3.18 – 3.04 (m, 1H), 2.94 – 2.82 (m, 1H), 2.78 (d, *J* = 12.4 Hz, 1H), 2.32 – 2.13 (m, 1H), 1.65 (d, *J* = 4.8 Hz, 1H), 1.55 (s, 1H), 1.28 (t, *J* = 6.1 Hz, 11H), 0.87 (t, *J* = 6.7 Hz, 3H).
^{13}C NMR (101 MHz, $CDCl_3$) δ 176.8, 176.7, 163.0(t, *J* = 32.2 Hz), 160.1, 159.4, 149.4, 149.3, 136.9, 123.1, 123.0, 121.0, 120.5, 116.4(t, *J* = 252.0 Hz), 88.7, 74.90, 39.9, 35.5 (t, *J* = 25.3 Hz), 34.8, 31.5, 28.9, 25.1, 25.0, 22.5, 19.6, 19.5, 14.0.
^{19}F NMR (376 MHz, $CDCl_3$) δ **-**104.04 (dd, *J* = 260.9, 91.9 Hz), -106.99 (dd, *J* = 260.8, 21.8 Hz).
HRMS (ESI-TOF) m/z: $[M + H]^+$ calculated for $C_{27}H_{30}F_2N_2O_4$ 447.2090; Found 447.2184.

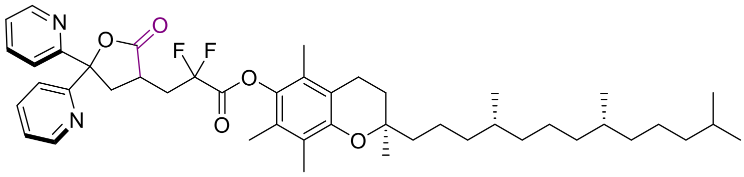

(R)-2,5,7,8-tetramethyl-2-((4R,8R)-4,8,12-trimethyltridecyl)chroman-6-yl 2,2-difluoro-3-(2-oxo-5,5-di(pyridin-2-yl)tetrahydrofuran-3-yl)propanoate (3ag)

Yellow oil, 85.3 mg, 56% yield, R_f = 0.3 (PE/EA = 3/1, 1% NEt_3)

^{1}H NMR (400 MHz, $CDCl_3$) δ 8.68 – 8.61 (m, 2H), 7.69 (m, 2H), 7.50 (d, *J* = 8.0 Hz, 1H), 7.45 – 7.38 (m, 1H), 7.23 (dd, *J* = 7.4, 1.1 Hz, 2H), 3.97 (dd, *J* = 13.2, 8.6 Hz, 1H), 3.32 – 3.19 (m, 1H), 3.16 – 2.97 (m, 1H), 2.93 – 2.82 (m, 1H), 2.59 (t, *J* = 6.8 Hz, 2H), 2.52 – 2.35 (m, 1H), 2.09 (s, 3H), 1.97 (d, *J* = 15.2 Hz, 6H), 1.79 (m,, 2H), 1.52 (p, *J* = 6.6 Hz, 3H), 1.46 – 1.25 (m, 10H), 1.24 (s, 5H), 1.16 – 1.04 (m, 6H), 0.85 (dd, *J* = 8.6, 6.5 Hz, 12H).

^{13}C NMR (101 MHz, $CDCl_3$) δ 176.8, 162.1(t, *J* = 33.3 Hz),160.1, 159.5, 150.0, 149.4, 139.5, 137.0, 136.9, 126.1, 124.5, 123.5, 123.2, 123.1, 121.0, 120.6, 117.7, 115.6(t, *J* = 250.5 Hz), 88.9, 75.3, 40.1, 39.4, 37.4, 37.3, 35.6(t, *J* = 24.4 Hz), 34.9, 32.8, 32.7, 31.0, 28.0, 24.8, 24.4, 23.9, 22.7, 22.6, 21.0, 20.5, 19.7, 19.6, 12.7, 11.8.

^{19}F NMR (376 MHz, $CDCl_3$) δ -102.01 – -108.08 (m).

HRMS (ESI-TOF) m/z: $[M + H]^+$ calculated for $C_{46}H_{62}F_2N_2O_5$ 761.4700; Found 761.4689.

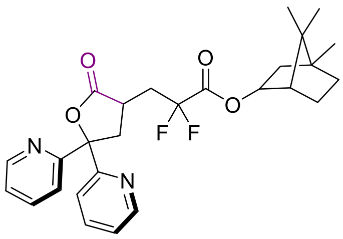

(1S,4S)-4,7,7-trimethylbicyclo[2.2.1]heptan-2-yl 2,2-difluoro-3-(2-oxo-5,5-di(pyridin-2-yl)tetrahydrofuran-3-yl)propanoate (3ah)

Colorless oil, 53.4 mg, 55% yield, R_f = 0.3 (PE/EA = 3/1, 1% NEt_3)

^{1}H NMR (400 MHz, $CDCl_3$) δ 8.63 (d, *J* = 5.0 Hz, 2H), 7.74 – 7.63 (m, 2H), 7.48 (d, *J* = 8.0 Hz, 1H), 7.40 (dd, *J* = 8.0, 4.1 Hz, 1H), 7.29 – 7.19 (m, 2H), 4.84 – 4.75 (m, 1H), 3.91 – 3.81 (m, 1H), 3.17 – 3.03 (m, 1H), 2.86 (d, *J* = 29.6 Hz, 1H), 2.77 (d, *J* = 11.4 Hz, 1H), 2.30 – 2.11 (m, 1H), 1.91 – 1.65 (m, 5H), 1.58 (t, *J* = 12.4 Hz, 1H), 1.24 – 0.99 (m, 3H), 0.95 (s, 3H), 0.83 (d, *J* = 9.6 Hz, 6H).

^{13}C NMR (101 MHz, $CDCl_3$) δ 176.7, 163.0 (t, *J* = 33.3 Hz), 159.4, 149.4, 149.3, 136.9, 123.1, 123.0, 120.9, 120.6, 115.1(t, *J* = 251.5 Hz), 88.7, 84.1, 49.1, 49.0, 46.9, 44.9, 39.9, 38.3, 38.2, 35.5 (t, *J* = 24.1 Hz), 34.8, 33.4, 29.6, 26.8, 19.9, 19.7, 19.6, 11.2.

^{19}F NMR (376 MHz, $CDCl_3$) δ -103.57 (dd, *J* = 261.6, 43.0 Hz), -107.13 (dd, *J* = 261.5, 4.9 Hz).

HRMS (ESI-TOF) m/z: $[M + H]^+$ calculated for $C_{27}H_{30}F_2N_2O_4$ 485.2246; Found 485.2246.

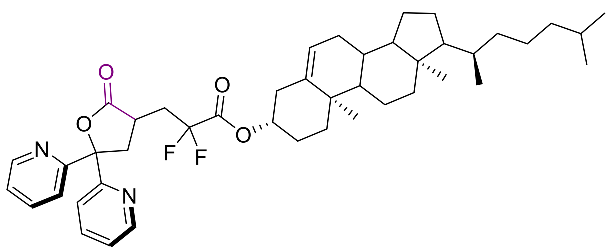

(3S,10R,13R)-10,13-dimethyl-17-((R)-6-methylheptan-2-yl)-2,3,4,7,8,9,10,11,12,13,14,15,16,17-tetradecahydro-1H-cyclopenta[a]phenanthren-3-yl 2,2-difluoro-3-(2-oxo-5,5-di(pyridin-2-yl)tetrahydrofuran-3-yl)propanoate (3ai)

Colorless oil, 51.7 mg, 36% yield, R_f = 0.3 (PE/EA = 3/1, 1% NEt_3)

^{1}H NMR (400 MHz, CDCl$_3$) δ 8.63 (d, *J* = 4.7 Hz, 2H), 7.68 (m, 2H), 7.48 (d, *J* = 7.9 Hz, 1H), 7.41 (d, *J* = 7.9 Hz, 1H), 7.23 (dd, *J* = 7.5, 4.8 Hz, 2H), 5.43 – 5.35 (m, 1H), 4.74 (dt, *J* = 11.1, 5.1 Hz, 1H), 3.86 (dd, *J* = 13.1, 8.6 Hz, 1H), 3.09 (d, *J* = 10.0 Hz, 1H), 2.93 – 2.71 (m, 2H), 2.43 – 2.13 (m, 3H), 2.05 – 1.95 (m, 2H), 1.88 (d, *J* = 24.7 Hz, 2H), 1.71 – 1.60 (m, 2H), 1.60 – 1.44 (m, 6H), 1.36 (d, *J* = 15.4 Hz, 3H), 1.27 (s, 2H), 1.17 – 1.07 (m, 6H), 1.00 (d, *J* = 14.6 Hz, 5H), 0.94 – 0.89 (m, 4H), 0.87 (d, *J* = 7.0 Hz, 6H), 0.68 (s, 3H).

^{13}C NMR (101 MHz, CDCl$_3$) δ 176.8, 163.1(t, *J* = 32.1 Hz), 160.0, 159.4, 149.4, 138.7, 136.9, 123.4, 123.1, 120.9, 120.6,115.1(t, *J* = 254.5 Hz), 88.8, 56.6, 56.1, 49.9, 42.3, 39.7, 39.5, 37.6, 36.8, 36.5, 36.2, 35.8,35.5 (t, *J* = 24.4 Hz), 34.9, 31.8, 28.2, 28.0, 27.3, 24.3, 23.8, 22.8, 22.5, 21.0, 19.3, 18.7, 11.8.

^{19}F NMR (376 MHz, CDCl$_3$) δ -103.33 – -107.67 (m).

HRMS (ESI-TOF) m/z: [M + H]$^+$ calculated for $C_{44}H_{58}F_2N_2O_4$ 717.4435; Found 717.4437.

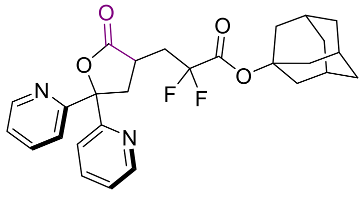

(*3S,5S,7S*)-adamantan-1-yl 2,2-difluoro-3-(2-oxo-5,5-di(pyridin-2-yl)tetrahydrofuran-3-yl) Propanoate (3aj)

Colorless oil, 46.4 mg, 48% yield (95% purity), R_f = 0.3 (PE/EA = 3/1, 1% NEt_3)

^{1}H NMR (400 MHz, CDCl$_3$) δ 8.63 (d, *J* = 4.9 Hz, 2H), 7.74 – 7.63 (m, 2H), 7.49 (d, *J* = 7.9 Hz, 1H), 7.41 (d, *J* = 8.0 Hz, 1H), 7.27 – 7.19 (m, 2H), 3.85 (dd, *J* = 13.2, 8.6 Hz, 1H), 3.15 – 3.02 (m, 1H), 2.90 – 2.71 (m, 2H), 2.20 (s, 4H), 2.12 (s, 6H), 1.66 (s, 6H).

^{13}C NMR (101 MHz, CDCl$_3$) δ 176.8, 162.0(t, *J* = 31.6 Hz), 160.1, 159.5, 149.3, 149.3, 136.9, 136.85, 123.1, 123.0, 120.9, 120.5, 114.9 (t, *J* = 251.5 Hz), 88.7, 85.0, 40.9, 39.8, 35.8, 35.5(t, *J* = 23.9 Hz), 35.0 (t, *J* = 3.6 Hz), 30.9, .

^{19}F NMR (376 MHz, CDCl$_3$) δ -103.82 (d, *J* = 257.5 Hz), -106.63 (dd, *J* = 257.5, 2.8 Hz).

HRMS (ESI-TOF) m/z: [M + H]$^+$ calculated for $C_{27}H_{28}F_2N_2O_4$ 483.2090; Found 483.2102.

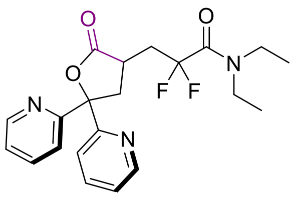

N,N-diethyl-2,2-difluoro-3-(2-oxo-5,5-di(pyridin-2-yl)tetrahydrofuran-3-yl)propanamide (3ak)

Colorless oil, 39.0 mg, 48% yield, R_f = 0.3 (PE/EA = 3/1, 1% NEt_3)

^{1}H NMR (400 MHz, $CDCl_3$) δ 8.63 (t, *J* = 5.4 Hz, 2H), 7.68 (q, *J* = 7.4 Hz, 2H), 7.52 (d, *J* = 8.0 Hz, 1H), 7.46 (d, *J* = 7.9 Hz, 1H), 7.22 (t, *J* = 5.6 Hz, 2H), 3.94 – 3.84 (m, 1H), 3.51 (q, *J* = 7.1 Hz, 2H), 3.37 (q, *J* = 7.1 Hz, 2H), 3.19 – 3.06 (m, 1H), 3.05 – 2.86 (m, 1H), 2.78 (t, *J* = 12.5 Hz, 1H), 2.34 (m, 1H), 1.17 (m, 7H).

^{13}C NMR (101 MHz, $CDCl_3$) δ 177.2, 162.0(t, *J* = 29.3 Hz), 160.4, 159.7, 149.3, 136.9, 123.0, 122.9, 120.9, 120.3, 118.8(t, *J* = 256.5 Hz), 88.6, 41.73(t, *J* = 7.1 Hz), 41.5, 40.1, 35.9 (t, *J* = 23.5 Hz), 35.0, 14.2, 12.2.

^{19}F NMR (376 MHz, $CDCl_3$) δ -98.05 (d, *J* = 279.3 Hz), -100.64 (d, *J* = 279.2 Hz).

HRMS (ESI-TOF) m/z: $[M + H]^+$ calculated for $C_{21}H_{23}F_2N_3O_3$ 404.1780; Found 404.1789.

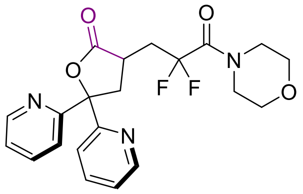

3-(2,2-difluoro-3-morpholino-3-oxopropyl)-5,5-di(pyridin-2-yl)dihydrofuran-2(3H)-one(3al)

Colorless oil, 63.0 mg, 77% yield, R_f = 0.5 (PE/EA / DCM/ MeOH= 1/1/1/0.05, 1% NEt_3)

^{1}H NMR (400 MHz, $CDCl_3$) δ 8.63 (t, *J* = 5.3 Hz, 2H), 7.74 – 7.63 (m, 2H), 7.51 (d, *J* = 7.9 Hz, 1H), 7.45 (d, *J* = 7.9 Hz, 1H), 7.22 (d, *J* = 4.9 Hz, 2H), 3.88 (dd, *J* = 12.3, 9.4 Hz, 1H), 3.71 (d, *J* = 4.7 Hz, 6H), 3.64 (s, 2H), 3.13 (q, *J* = 11.5 Hz, 1H), 3.07 – 2.88 (m, 1H), 2.79 (t, *J* = 12.5 Hz, 1H), 2.44 – 2.24 (m, 1H).

^{13}C NMR (101 MHz, $CDCl_3$) δ 177.0, 161.1 (t, *J* = 29.2 Hz), 160.3, 159.6, 149.3, 136.9, 123.0, 122.9, 120.8, 120.4, 118.7 (t, *J* = 256.5 Hz), 88.6, 66.7, 66.6, 46.4, 43.3, 40.0, 35.7 (t, *J* = 23.2 Hz), 34.9.

^{19}F NMR (376 MHz, $CDCl_3$) δ -97.22 (d, *J* = 282.7 Hz), -99.87 (d, *J* = 282.7 Hz).

HRMS (ESI-TOF) m/z: $[M + H]^+$ calculated for $C_{21}H_{21}F_2N_3O_4$ 408.1570; Found 408.1573.

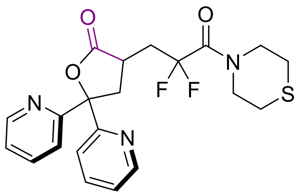

4-(2,2-difluoro-3-oxo-3-thiomorpholinopropyl)-5,5-di(pyridin-2-yl)dihydrofuran-2(3H)-one (3am)

Colorless oil, 70.3 mg, 81% yield, R_f = 0.5 (PE/EA / DCM/ MeOH= 1/1/1/0.05, 1% NEt_3)

^{1}H NMR (400 MHz, CDCl3) δ 8.63 (t, *J* = 5.2 Hz, 2H), 7.74 – 7.64 (m, 2H), 7.51 (d, *J* = 8.0 Hz, 1H), 7.45 (d, *J* = 8.0 Hz, 1H), 7.22 (t, *J* = 6.3 Hz, 2H), 3.96 (s, 2H), 3.88 (d, *J* = 3.5 Hz, 3H), 3.19 – 3.07 (m, 1H), 3.07 – 2.88 (m, 1H), 2.79 (t, *J* = 12.5 Hz, 1H), 2.66 (d, *J* = 5.9 Hz, 4H), 2.44 – 2.24 (m, 1H).
^{13}C NMR (101 MHz, $CDCl_3$) δ 177.0, 161.3(t, *J* = 29.0 Hz), 160.3, 159.6, 149.3, 136.9, 136.9, 123.0, 122.9, 120.9, 120.4, 117.5(t, *J* = 256.1 Hz), 88.6, 48.6, 45.9, 40.1, 40.0, 35.8(t, *J* = 23.1 Hz), 34.9, 28.0, 27.2.
^{19}F NMR (376 MHz, $CDCl_3$) δ -97.35 (d, *J* = 282.5 Hz), -100.02 (d, *J* = 282.5 Hz).
HRMS (ESI-TOF) m/z: $[M + H]^+$ calculated for $C_{21}H_{21}F_2N_3O_3S$ 434.1350; Found 434.1344.

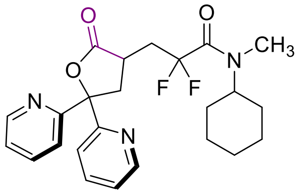

N-cyclohexyl-2,2-difluoro-N-methyl-3-(2-oxo-5,5-di(pyridin-2-yl)tetrahydrofuran-3-yl)propanamide (3an)
Colorless oil, 57.0 mg, dr:=1:1, 64% yield, R_f = 0.3 (PE/EA = 2/1, 1% NEt_3)
^{1}H NMR (400 MHz, $CDCl_3$) δ 8.67 – 8.59 (m, 4H), 7.73 – 7.63 (m, 4H), 7.49 (dd, *J* = 19.7, 8.0 Hz, 4H), 7.21 (m, 4H), 4.27 (t, *J* = 11.0 Hz, 1H), 4.01 (t, *J* = 11.6 Hz, 1H), 3.94 – 3.83 (m, 2H), 3.13 (m, 2H), 2.99 (t, *J* = 2.2 Hz, 3H), 2.83 (s, 3H), 2.78 (dd, *J* = 12.4, 5.7 Hz, 2H), 2.43 – 2.24 (m, 2H), 1.82 (s, 4H), 1.68 (s, 6H), 1.58 – 1.18 (m, 10H), 1.14 (m, 2H).
^{13}C NMR (101 MHz, $CDCl_3$) δ 177.2, 162.24 (t, *J* = 30.0 Hz), 160.3, 159.7, 149.3, 136.9, 136.8, 122.9, 120.9, 120.8, 120.4, 120.3, 118.9 (t, *J* = 254.5 Hz), 88.5, 56.1(t, *J* = 6.3 Hz), 54.4, 40.1, 36.1(t, *J* = 23.2 Hz), 35.93 (t, *J* = 23.2 Hz),35.0 (t, *J* = 3.1 Hz) 30.8, 30.7, 29.1, 29.0, 28.9, 28.5, 25.5, 25.4, 25.2.
^{19}F NMR (376 MHz, $CDCl_3$) δ -96.02 – -102.85 (m).
HRMS (ESI-TOF) m/z: $[M + H]^+$ calculated for $C_{24}H_{27}F_2N_3O_3$ 444.2093; Found 444.2100.

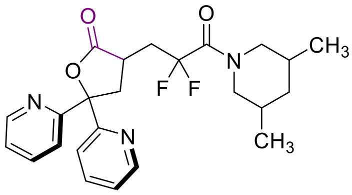

3-(3-(3,5-dimethylpiperidin-1-yl)-2,2-difluoro-3-oxopropyl)-5,5-di(pyridin-2-yl)dihydrofuran-2(3H)-one (3ao)
Colorless oil, 58.6 mg, 66% yield, R_f = 0.3 (PE/EA = 2/1, 1% NEt_3)
^{1}H NMR (400 MHz, $CDCl_3$) δ 8.67 – 8.59 (m, 2H), 7.68 (q, *J* = 7.1 Hz, 2H), 7.51 (d, *J* = 7.9 Hz, 1H), 7.46 (d, *J* = 8.0 Hz, 1H), 7.22 (t, *J* = 5.2 Hz, 2H), 4.44 (d, *J* = 12.7 Hz, 1H), 4.14 (d, *J* = 13.7 Hz, 1H), 3.88 (dd, *J* = 13.2, 8.7 Hz, 1H), 3.12 (dd, *J* = 11.2, 6.0 Hz, 1H), 3.06 – 2.86 (m, 1H), 2.80 (t, *J* = 12.5 Hz, 1H), 2.50 (dd, *J* = 13.5, 11.5 Hz, 1H), 2.43 – 2.22 (m, 1H), 2.11 (t, *J* = 12.2 Hz, 1H), 1.84 (d, *J* = 12.9 Hz, 1H), 1.71 – 1.52 (m, 2H), 1.27 – 1.11 (m, 1H), 0.90 (dd, *J* = 6.6, 4.3 Hz, 6H).

^{13}C NMR (101 MHz, CDCl$_3$) δ 177.1, 160.8(t, *J* = 28.5 Hz), 160.3, 159.6, 149.3, 149.26, 136.9, 136.8, 123.0, 122.9, 120.8, 120.4, 120.3, 118.8(t, *J* = 256.1 Hz), 88.5, 52.6(t, *J* = 6.1 Hz), 50.2, 42.2, 40.1 (t, *J* = 85.85 Hz), 35.9(t, *J* = 23.3 Hz), 34.9, 32.1, 31.0, 19.0, 18.7, 17.8.
^{19}F NMR (376 MHz, CDCl$_3$) δ -95.53 – -102.06 (m).
HRMS (ESI-TOF) m/z: $[M + H]^+$ calculated for $C_{24}H_{27}F_2N_3O_3$ 444.2093; Found 444.2104.

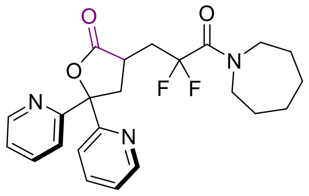

4-(3-(azepan-1-yl)-2,2-difluoro-3-oxopropyl)-5,5-di(pyridin-2-yl)dihydrofuran-2(3H)-one (3ap)
White solid, 41.3 mg,48% yield, R_f = 0.3 (PE/EA = 2/1, 1% NEt_3)
^{1}H NMR (400 MHz, CDCl$_3$) δ 8.63 (t, *J* = 4.8 Hz, 2H), 7.68 (m, 2H), 7.52 (d, *J* = 8.0 Hz, 1H), 7.46 (d, *J* = 7.9 Hz, 1H), 7.26 – 7.17 (m, 2H), 3.89 (dd, *J* = 13.3, 8.6 Hz, 1H), 3.65 (t, *J* = 6.1 Hz, 2H), 3.51 (t, *J* = 6.0 Hz, 2H), 3.20 – 3.07 (m, 1H), 3.07 – 2.88 (m, 1H), 2.78 (t, *J* = 12.5 Hz, 1H), 2.34 (m, 1H), 1.74 (s, 4H), 1.57 (s, 4H).
^{13}C NMR (101 MHz, CDCl$_3$) δ 177.2, 162.4(t, *J* = 28.4 Hz), 160.4, 159.7, 149.3, 136.8, 122.9, 120.9, 120.3, 117.6(t, *J* = 255.9 Hz), 88.5, 48.2, 47.7(t, *J* = 6.2 Hz), 40.1, 36.0 (t, *J* = 23.4 Hz), 35.0(t, *J* = 3.8 Hz), 29.6, 27.3, 26.3, 25.9.
^{19}F NMR (376 MHz, CDCl$_3$) δ -97.45 (d, *J* = 280.0 Hz), -100.12 (d, *J* = 280.0 Hz).
HRMS (ESI-TOF) m/z: $[M + H]^+$ calculated for $C_{23}H_{25}F_2N_3O_3$ 430.1937; Found 430.1943.

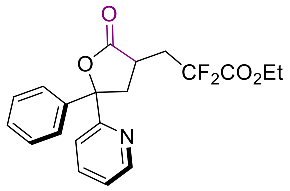

ethyl 2,2-difluoro-3-(2-oxo-5-phenyl-5-(pyridin-2-yl)tetrahydrofuran-3-yl)propanoate (3ba)
Colorless oil, 58.0 mg, 77% yield, R_f = 0.3 (PE/EA = 4/1, 1% NEt_3)
^{1}H NMR (400 MHz, CDCl$_3$) δ 8.59 (d, *J* = 4.9 Hz, 1H), 7.63 (t, *J* = 7.8 Hz, 1H), 7.55 (d, *J* = 7.6 Hz, 2H), 7.43 (d, *J* = 8.0 Hz, 1H), 7.40 – 7.33 (m, 2H), 7.32 – 7.27 (m, 1H), 7.19 (dd, *J* = 7.5, 4.8 Hz, 1H), 4.31 (q, *J* = 7.1 Hz, 2H), 3.36 (dd, *J* = 12.7, 7.4 Hz, 1H), 3.04 – 2.88 (m, 2H), 2.88 – 2.73 (m, 1H), 2.31 – 2.12 (m, 1H), 1.31 (t, *J* = 7.1 Hz, 3H).
^{13}C NMR (101 MHz, CDCl$_3$) δ 176.3, 163.5 (t, *J* = 32.3 Hz), 160.9, 148.9, 141.5, 136.9, 128.7, 128.1, 125.0, 122.9, 120.7, 115.0(t, *J* = 251.5 Hz), 88.2, 63.2, 41.5, 35.2 (t, *J* = 23.6 Hz), 34.3, 13.8.
^{19}F NMR (376 MHz, CDCl$_3$) δ -103.89 (d, *J* = 261.1 Hz), -107.07 (d, *J* = 261.5 Hz).
HRMS (ESI-TOF) m/z: $[M + H]^+$ calculated for $C_{20}H_{19}F_2NO_4$ 376.1354; Found 376.1355.

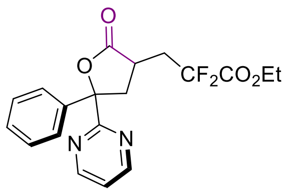

Ethyl 2,2-difluoro-3-(2-oxo-5-phenyl-5-(pyrimidin-2-yl)tetrahydrofuran-3-yl) propanoate (3ca)

Colorless oil, 45.1 mg, 60% yield, R_f = 0.3 (PE/EA = 4/1, 1% NEt_3)

^{1}H NMR (400 MHz, $CDCl_3$) δ 8.59 (d, *J* = 4.8 Hz, 1H), 7.63 (t, *J* = 7.7 Hz, 1H), 7.55 (d, *J* = 7.6 Hz, 2H), 7.43 (d, *J* = 8.4 Hz, 1H), 7.39 – 7.33 (m, 2H), 7.23 – 7.16 (m, 1H), 4.31 (q, *J* = 7.1 Hz, 2H), 3.36 (dd, *J* = 13.1, 7.2 Hz, 1H), 3.03 – 2.73 (m, 3H), 2.22 (m, *J* = 22.0, 15.1, 10.2 Hz, 1H), 1.31 (t, *J* = 7.1 Hz, 3H).

^{13}C NMR (101 MHz, $CDCl_3$) δ 176.6, 176.3, 163.4 (t, *J* = 31.3 Hz), 160.9, 160.1, 148.9, 142.6, 141.5, 137.1, 136.9, 128.7, 128.5, 128.1, 127.9, 125.0, 124.7, 123.0, 122.9, 121.3, 120.7, 115.0(t, *J* = 252.5 Hz), 88.6, 88.2, 63.2, 41.5, 35.1(t, *J* = 23.2 Hz), 34.3, 13.8, 13.1.

^{19}F NMR (376 MHz, $CDCl_3$) δ -103.08 – -108.18 (m).

HRMS (ESI-TOF) m/z: $[M + H]^+$ calculated for $C_{19}H_{18}F_2N_2O_4$ 377.1307; Found 377.1363.

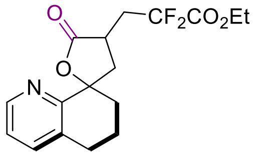

Ethyl 2,2-difluoro-3-(5-oxo-4,5,6',7'-tetrahydro-3H,5'H-spiro[furan-2,8'-quinolin]-4-yl) propanoate (3da)

Yellow solid, 22.5 mg, 33% yield, R_f = 0.3 (PE/EA = 4/1, 1% NEt_3)

^{1}H NMR (400 MHz, $CDCl_3$) δ 8.40 (d, *J* = 4.7 Hz, 1H), 7.42 (d, *J* = 7.9 Hz, 1H), 7.15 (dd, *J* = 7.8, 4.6 Hz, 12H), 4.35 (q, *J* = 7.1 Hz, 23H), 4.08 – 3.95 (m, 2H), 3.05 – 2.72 (m, 37H), 2.39 – 2.28 (m, 1H), 2.28 – 1.84 (m, 11H), 1.37 (t, *J* = 7.1 Hz, 34H).

^{13}C NMR (101 MHz, $CDCl_3$) δ 178.0, 163.6 (t, *J* = 32.4 Hz), 155.5, 147.8, 137.0, 131.8, 123.5, 115.5 (t, *J* = 251.7 Hz), 83.4, 63.1, 42.3, 37.6, 36.6 (t, *J* = 23.3 Hz), 36.3, 28.6, 19.3, 13.9.

^{19}F NMR (376 MHz, $CDCl_3$) δ -103.73 (d, *J* = 259.6 Hz), -106.97 (d, *J* = 259.6 Hz).

HRMS (ESI-TOF) m/z: $[M + H]^+$ calculated for $C_{17}H_{19}F_2NO_4$ 340.1355; Found 340.1347.

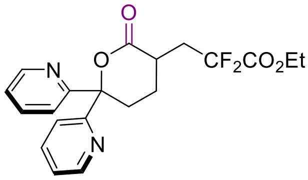

ethyl 2,2-difluoro-3-(2-oxo-6,6-di(pyridin-2-yl)tetrahydro-2H-pyran-3-yl)propanoate (3ga)

Colorless oil, 50.0 mg, 64% yield, R_f = 0.3 (PE/EA = 4/1, 1% NEt_3)

^{1}H NMR (400 MHz, $CDCl_3$) δ 8.62 (dd, *J* = 17.8, 5.7 Hz, 3H), 7.67 (t, *J* = 7.8 Hz, 3H), 7.53 (d, *J* = 8.0 Hz, 3H), 7.25 – 7.16 (m, 3H), 4.31 (q, *J* = 7.1 Hz, 3H), 3.17 – 3.06 (m, 2H), 3.06 – 2.95 (m, 1H), 2.81 – 2.69 (m, 3H), 2.20 (dd, *J* = 20.9, 12.6 Hz, 2H), 2.15 – 2.07 (m, 1H), 1.63 – 1.54 (m, 1H), 1.34 (t, *J* = 7.1 Hz, 3H).

^{13}C NMR (101 MHz, CDCl$_3$) δ 172.1, 163.7(t, J = 32.5 Hz), 160.4, 148.8, 136.9, 122.9, 122.6, 120.8, 116.8(t, J = 250.5 Hz), 88.9, 63.0, 36.2(t, J = 23.2 Hz), 35.4(t, J = 3.4 Hz), 31.4, 23.8, 13.8.
^{19}F NMR (376 MHz, CDCl$_3$) δ -101.91 (d, J = 258.9 Hz), -107.08 (d, J = 259.1 Hz).
HRMS (ESI-TOF) m/z: $[M + H]^+$ calculated for $C_{20}H_{20}F_2N_2O_4$ 391.1464; Found 391.1473.

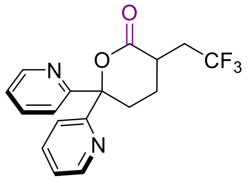

6,6-di(pyridin-2-yl)-3-(2,2,2-trifluoroethyl)tetrahydro-2H-pyran-2-one (3gq)
Colorless oil, 33.7 mg, 50% yield, R_f = 0.3 (PE/EA = 4/1, 1% NEt_3)
^{1}H NMR (400 MHz, CDCl$_3$) δ 8.68 – 8.57 (m, 2H), 7.73 – 7.63 (m, 2H), 7.57 – 7.47 (m, 2H), 7.26 – 7.16 (m, 2H), 3.20 – 3.02 (m, 2H), 2.81 – 2.67 (m, 2H), 2.31 – 2.12 (m, 2H), 1.62 – 1.47 (m, 1H).
^{13}C NMR (101 MHz, CDCl$_3$) δ 171.5, 160.4, 148.9, 137.0, 126.6(q, J = 276.8 Hz), 123.0, 122.8, 121.0, 89.0, 36.1, 35.6 (q, J = 29.3 Hz), 31.4, 23.1.
^{19}F NMR (376 MHz, CDCl$_3$) δ -63.84.
HRMS (ESI-TOF) m/z: $[M + H]^+$ calculated for $C_{17}H_{15}F_3N_2O_2$ 337.1158; Found 337.1164.

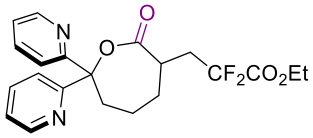

ethyl 2,2-difluoro-3-(2-oxo-7,7-di(pyridin-2-yl)oxepan-3-yl)propanoate (3ha)
Colorless oil, 36.5 mg, 45% yield, R_f = 0.3 (PE/EA = 4/1, 1% NEt_3)
^{1}H NMR (400 MHz, CDCl$_3$) δ 8.68 (d, J = 4.9 Hz, 1H), 8.50 (d, J = 4.4 Hz, 1H), 7.89 (d, J = 8.2 Hz, 1H), 7.73 – 7.61 (m, 2H), 7.49 (d, J = 8.0 Hz, 1H), 7.19 (dd, J = 7.5, 4.8 Hz, 1H), 7.14 – 7.06 (m, 1H), 4.27 (q, J = 7.1 Hz, 2H), 3.64 (d, J = 15.4 Hz, 1H), 3.01 – 2.80 (m, 1H), 2.45 – 2.26 (m, 2H), 2.23 – 2.06 (m, 1H), 2.06 – 1.88 (m, 2H), 1.78 (d, J = 11.7 Hz, 1H), 1.72 – 1.58 (m, 1H), 1.33 (t, J = 7.1 Hz, 3H).
^{13}C NMR (101 MHz, CDCl$_3$) δ 174.7, 163.8(t, J = 32.6 Hz), 162.3, 159.7, 149.0, 148.8, 137.1, 136.9, 122.5, 122.2, 121.7, 119.9, 115.1(t, J =250.5 Hz), 88.2, 62.9, 39.4(t, J = 3.7 Hz), 37.1(t, J = 23.1 Hz), 36.6, 30.8, 23.4, 13.9.
^{19}F NMR (376 MHz, CDCl$_3$) δ **-**102.39 (d, J = 259.9 Hz), **-**106.93 (d, J = 259.7 Hz).
HRMS (ESI-TOF) m/z: $[M + H]^+$ calculated for $C_{21}H_{22}F_2N_2O_4$ 405.1620; Found 405.1620.

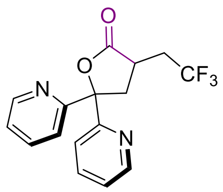

5,5-di(pyridin-2-yl)-3-(2,2,2-trifluoroethyl)dihydrofuran-2(3H)-one (3aq)

Colorless oil, 40.0 mg, 62% yield, R_f = 0.3 (PE/EA = 4/1, 1% NEt_3)

^{1}H NMR (400 MHz, $CDCl_3$) δ 8.65 (d, *J* = 5.2 Hz, 2H), 7.75 – 7.64 (m, 2H), 7.48 (d, *J* = 8.0 Hz, 1H), 7.40 (d, *J* = 7.9 Hz, 1H), 7.27 – 7.22 (m, 2H), 3.88 (dd, *J* = 13.1, 8.7 Hz, 1H), 3.19 – 3.05 (m, 1H), 2.84 (m, 2H), 2.26 (m, *J* = 15.1, 10.4 Hz, 1H).

^{13}C NMR (101 MHz, $CDCl_3$) δ 176.0, 159.9, 159.2, 149.4, 137.0, 126.2(q, *J* = 276.7 Hz), 123.2, 121.0, 120.5, 88.8, 39.4, 35.3(d, *J* = 3.0 Hz), 35.0 (q, *J* = 29.9 Hz), .

^{19}F NMR (376 MHz, $CDCl_3$) δ -65.26.

HRMS (ESI-TOF) m/z: $[M + H]^+$ calculated for $C_{16}H_{13}F_3N_2O_2$ 323.1002; Found 323.1010.

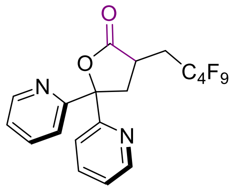

3-(2,2,3,3,4,4,5,5,5-nonafluoropentyl)-5,5-di(pyridin-2-yl)dihydrofuran-2(3H)-one (3ar)

Colorless oil, 33.1 mg, 35% yield, R_f = 0.3 (PE/EA = 4/1, 1% NEt_3)

^{1}H NMR (400 MHz, $CDCl_3$) δ 8.65 (t, *J* = 4.8 Hz, 2H), 7.70 (m, 2H), 7.48 (d, *J* = 7.9 Hz, 1H), 7.39 (d, *J* = 7.9 Hz, 1H), 7.24 (d, *J* = 6.2 Hz, 2H), 3.93 (m, 1H), 3.23 (q, *J* = 8.8 Hz, 1H), 3.00 – 2.76 (m, 2H), 2.34 – 2.17 (m, 1H).

^{13}C NMR (101 MHz, $CDCl_3$) δ 176.4, 159.9, 159.3, 149.4, 137.0, 123.3, 123.2, 121.0, 120.5, 117.7, 88.9, 39.9, 34.2, 32.2 (t, *J* = 21.6 Hz), .

^{19}F NMR (376 MHz, $CDCl_3$) δ -81.03, -112.01, -112.72, -114.58, -115.30, -124.48, -125.94.

HRMS (ESI-TOF) m/z: $[M + H]^+$ calculated for $C_{19}H_{13}F_9N_2O_2$ 473.0906; Found 473.0896.

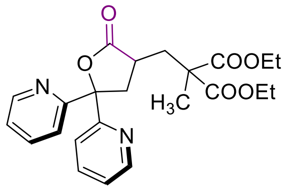

diethyl 2-methyl-2-((2-oxo-5,5-di(pyridin-2-yl)tetrahydrofuran-3-yl)methyl)malonate (3as)

Colorless oil, 34.1 mg, 40% yield, R_f = 0.3 (PE/EA = 4/1, 1% NEt_3)

^{1}H NMR (400 MHz, $CDCl_3$) δ 8.61 (t, *J* = 5.3 Hz, 2H), 7.72 – 7.61 (m, 2H), 7.55 – 7.48 (m, 1H), 7.43 (d, *J* = 8.0 Hz, 1H), 7.25 – 7.16 (m, 2H), 4.23 – 4.14 (m, 4H), 3.76 (dd, *J* = 12.6, 8.4 Hz, 1H), 2.84 – 2.72 (m, 1H), 2.70 – 2.59 (m, 2H), 2.08 (dd, *J* = 14.6, 9.3 Hz, 1H), 1.46 (s, 3H), 1.23 (t, *J* = 7.1 Hz, 6H).

^{13}C NMR (101 MHz, $CDCl_3$) δ 177.9, 171.7, 160.5, 159.7, 149.3, 136.9, 136.8, 123.0, 122.8, 120.9, 120.2, 88.2, 61.5, 52.8, 40.3, 37.0, 36.7, 20.5, 13.9.

HRMS (ESI-TOF) m/z: $[M + H]^+$ calculated for $C_{23}H_{26}N_2O_6$ 427.1864; Found 427.1869.

6. NMR Spectra of the Substrates and Products

Substrates

^{1}H NMR spectrum of **1a** in CDCl3 (400 MHz)

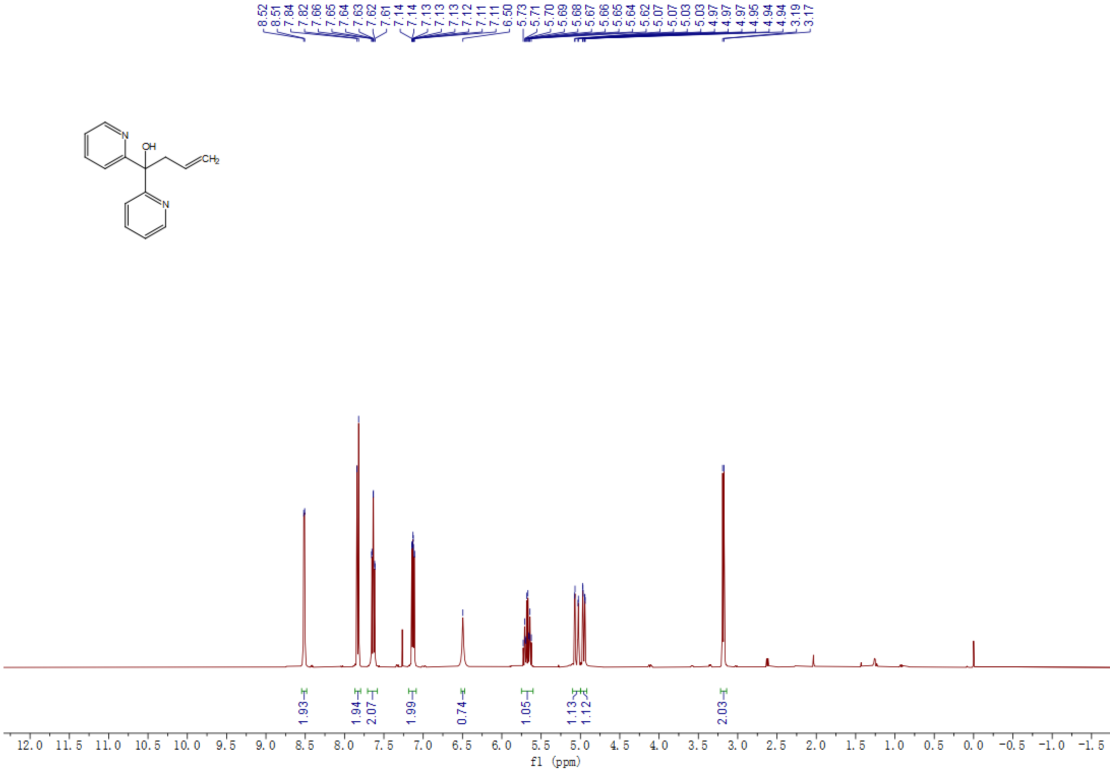

^{13}C NMR spectrum of **1a** in $CDCl_3$ (101 MHz)

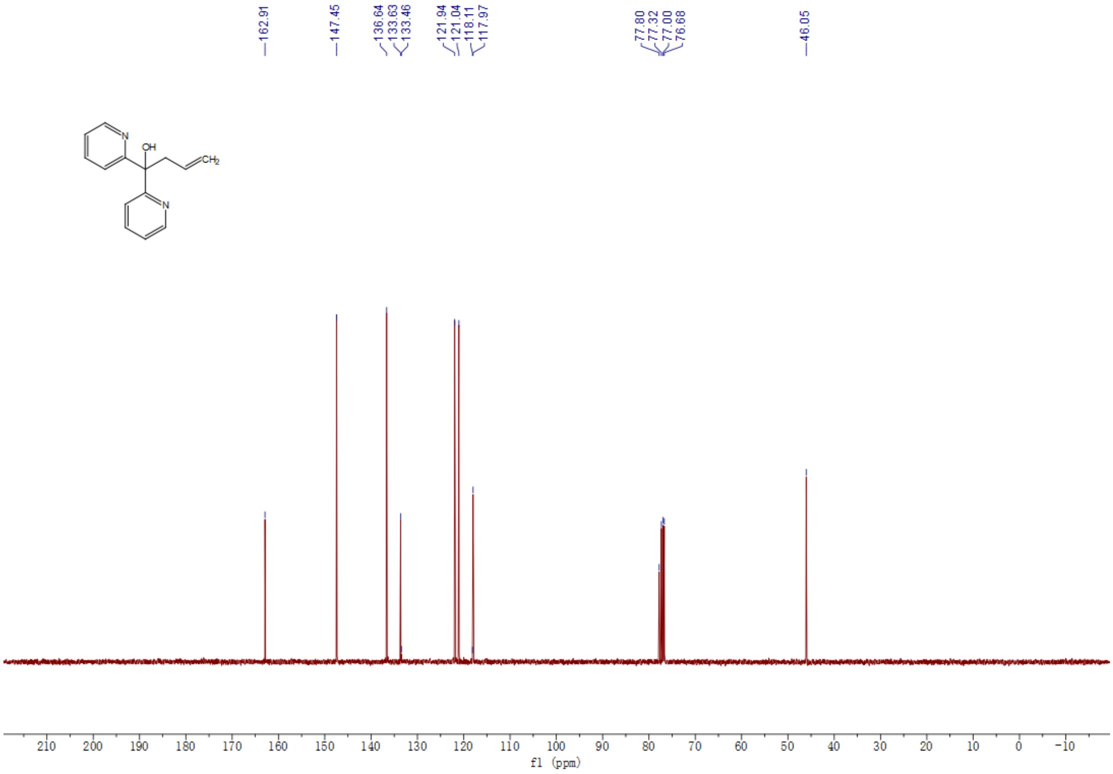

^{1}H NMR spectrum of **1b** in CDCl3 (400 MHz)

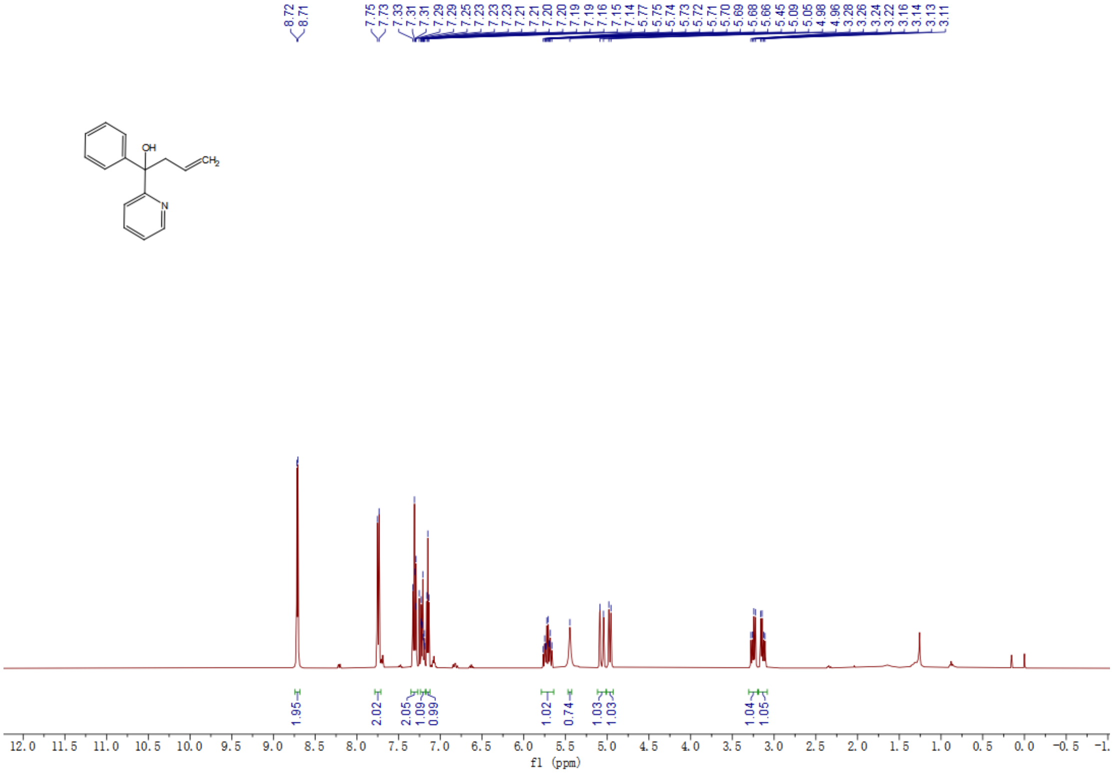

^{13}C NMR spectrum of **1b** in $CDCl_3$ (101 MHz)

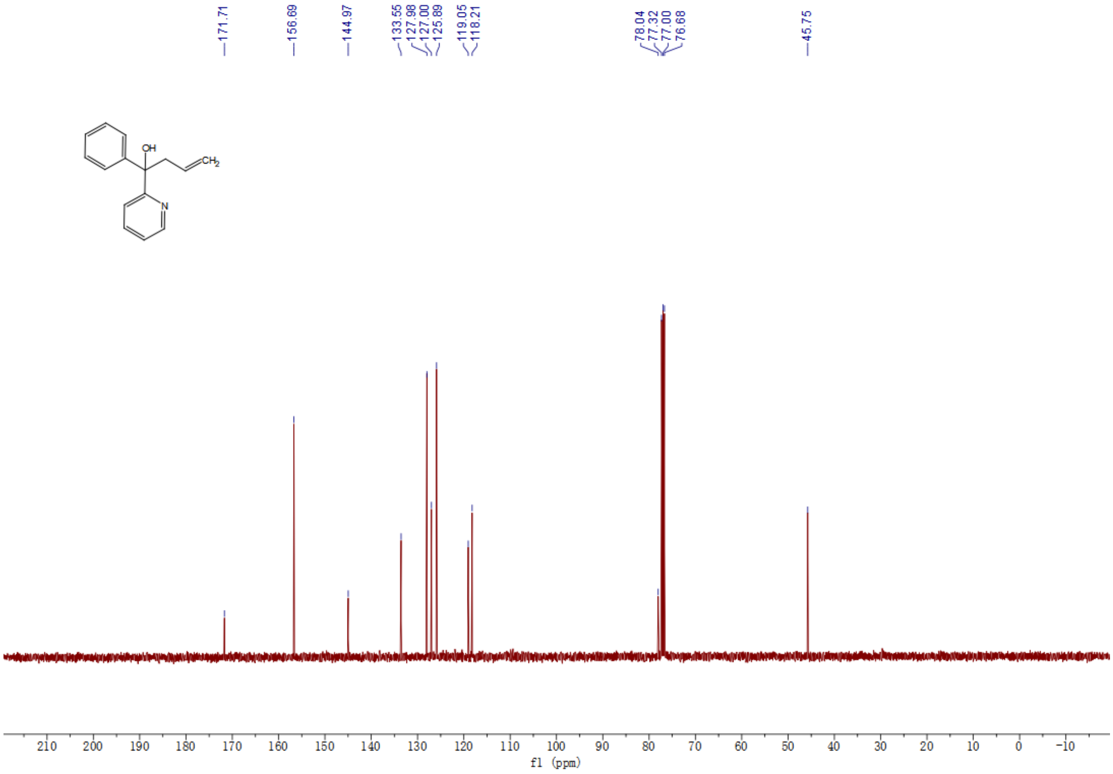

^{1}H NMR spectrum of **1d** in CDCl3 (400 MHz)

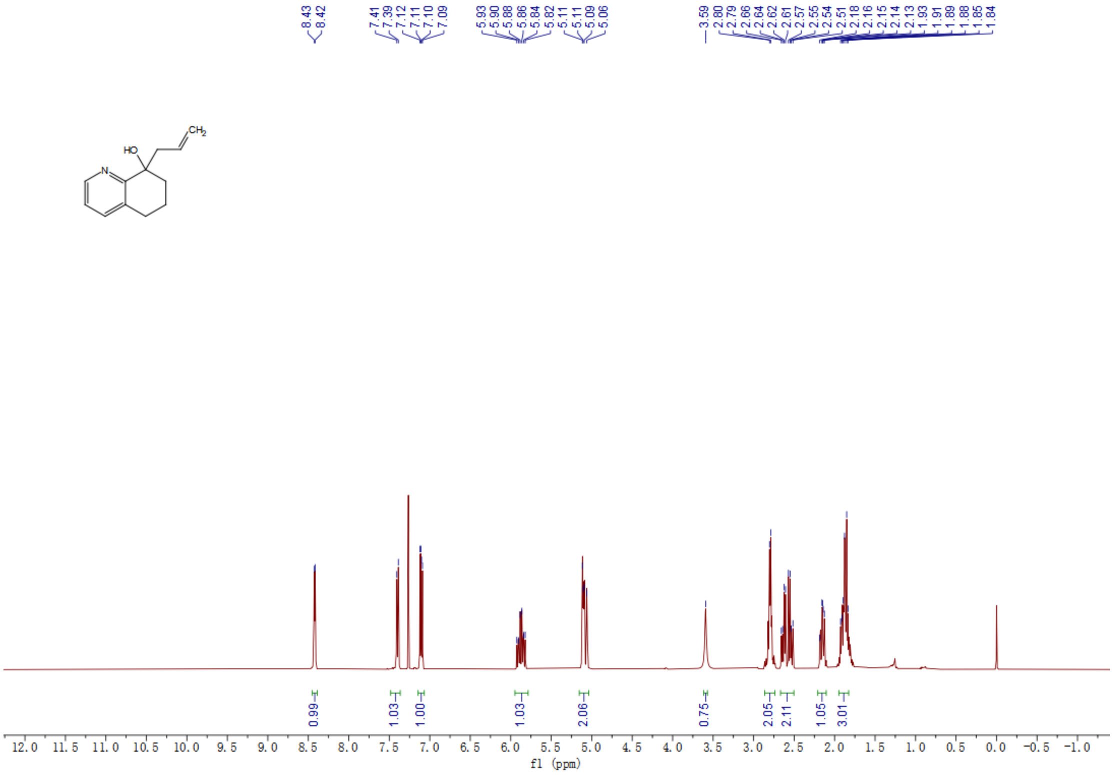

^{13}C NMR spectrum of **1d** in $CDCl_3$ (101 MHz)

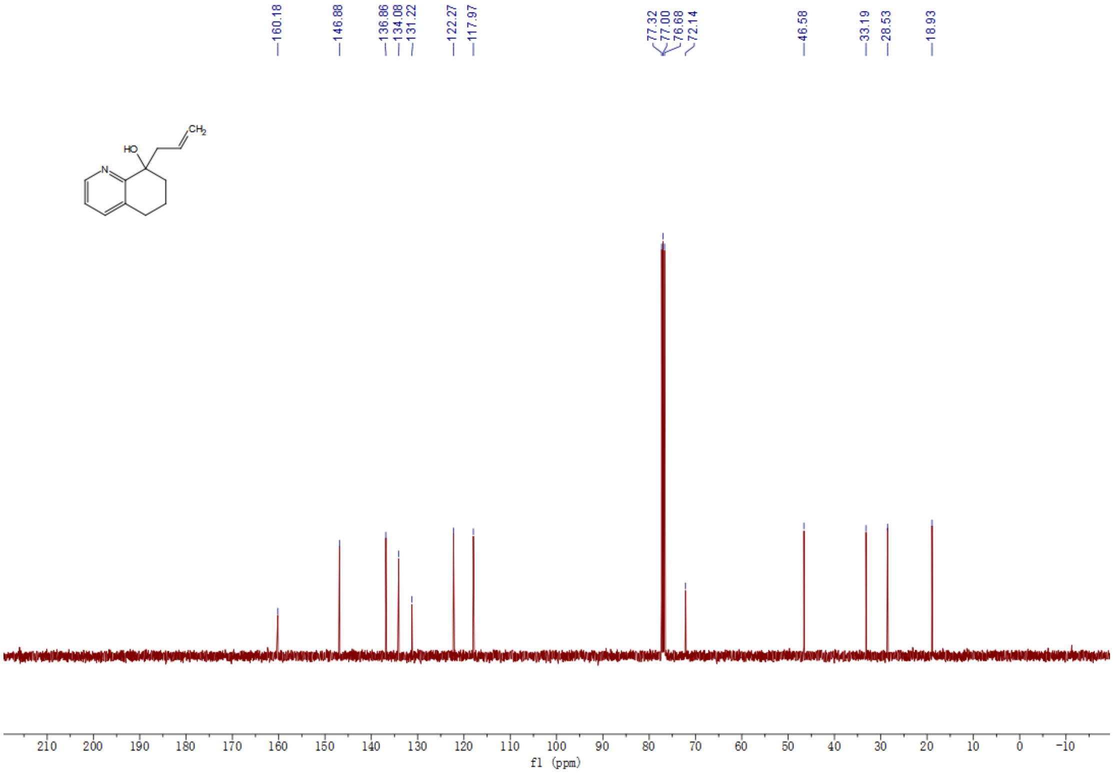

^{1}H NMR spectrum of **1f** in CDCl3 (400 MHz)

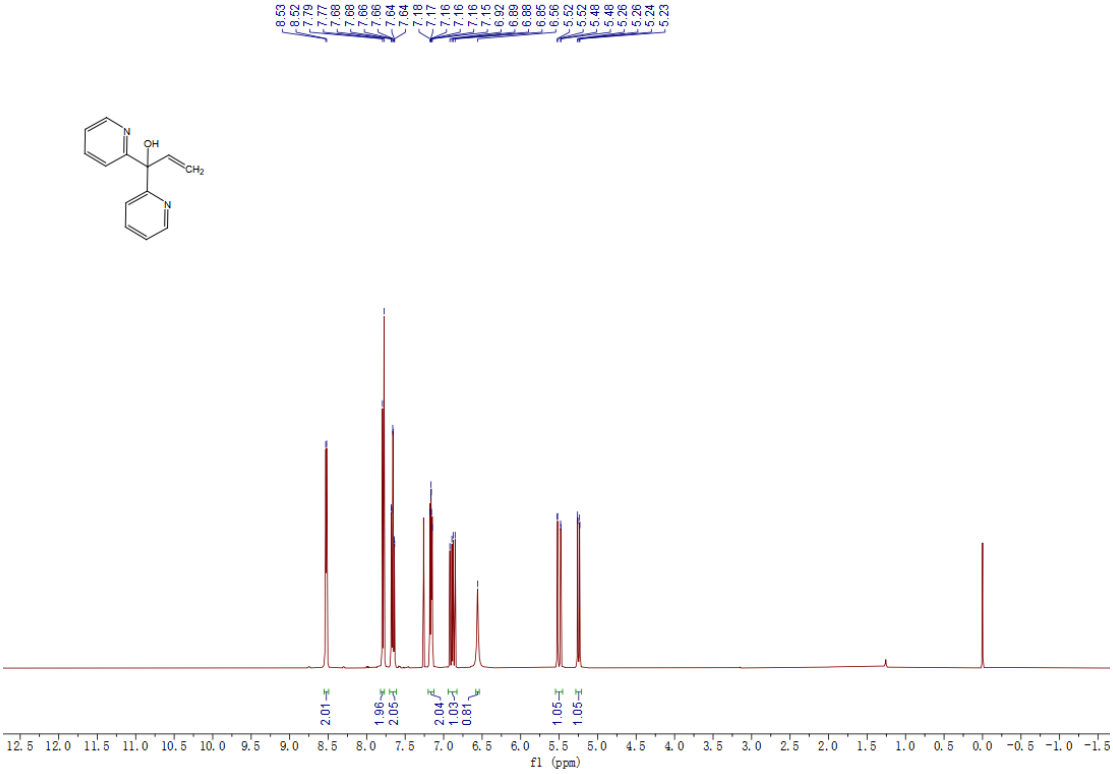

^{13}C NMR spectrum of **1f** in $CDCl_3$ (101 MHz)

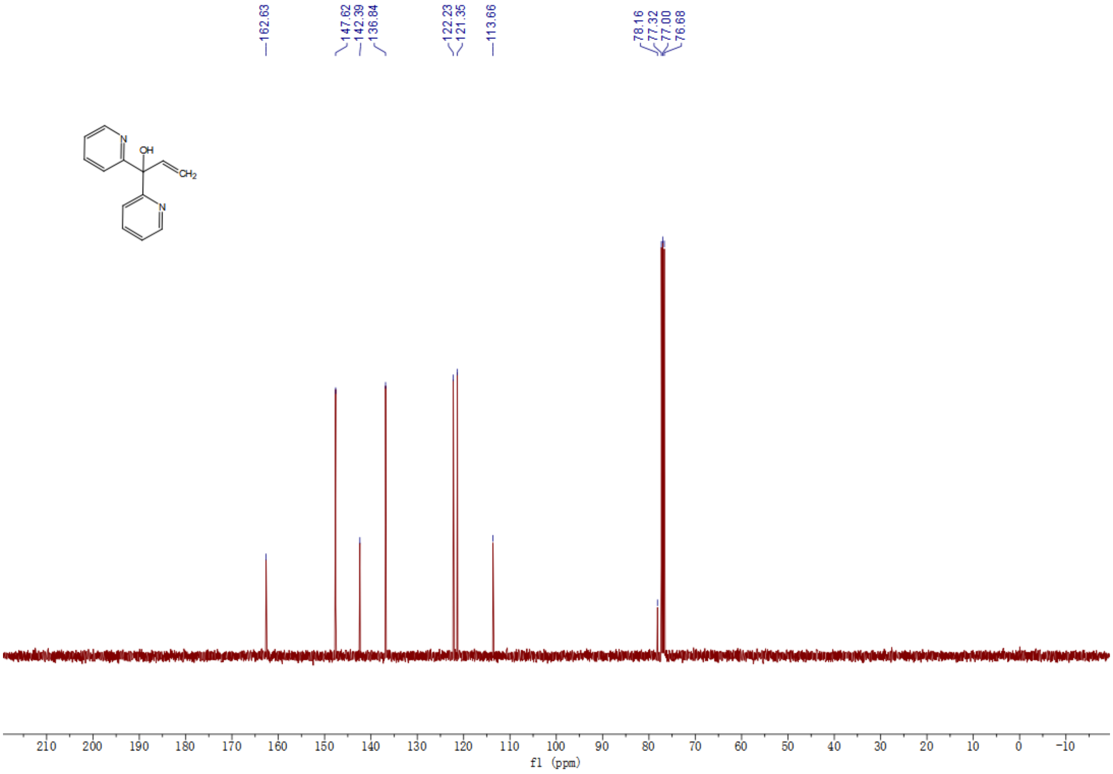

^{1}H NMR spectrum of **1g** in CDCl3 (400 MHz)

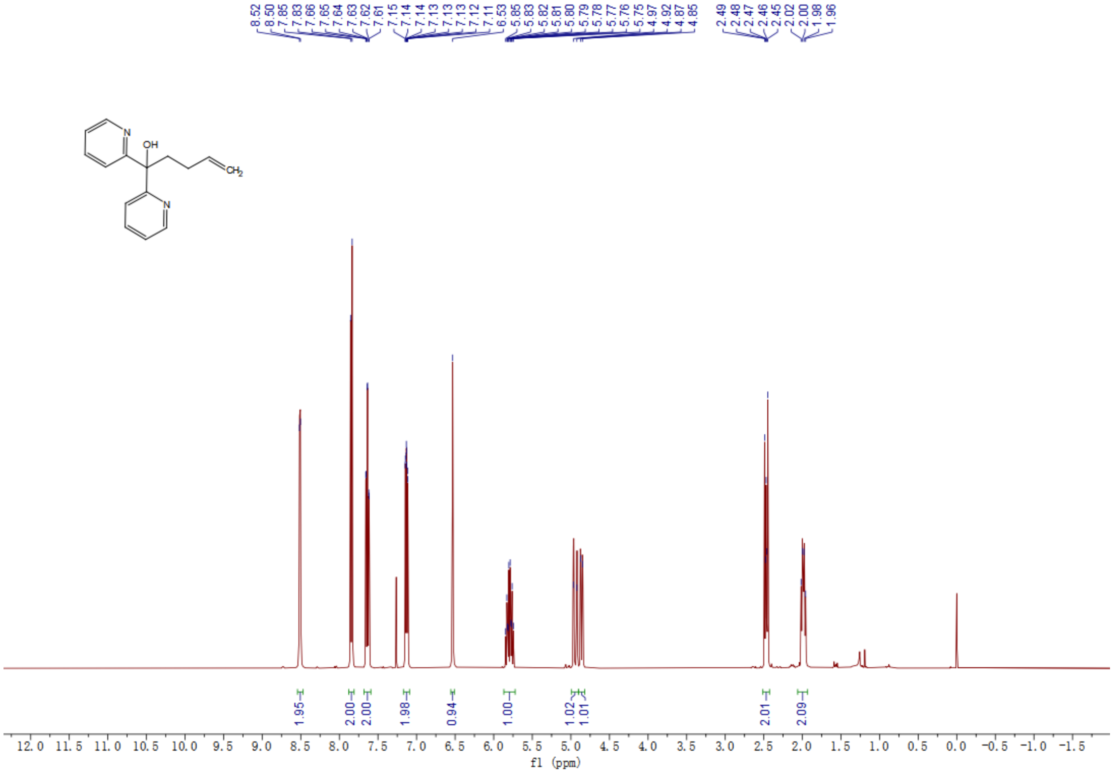

^{13}C NMR spectrum of **1g** in $CDCl_3$ (101 MHz)

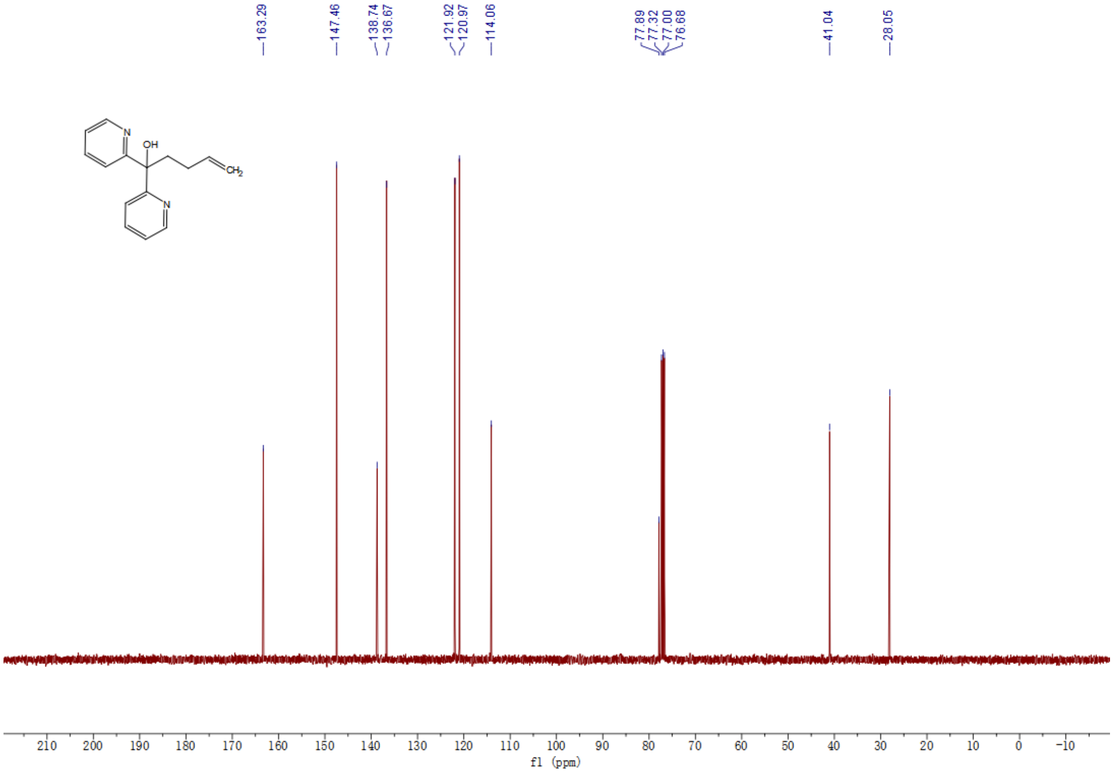

^{1}H NMR spectrum of **1h** in CDCl3 (400 MHz)

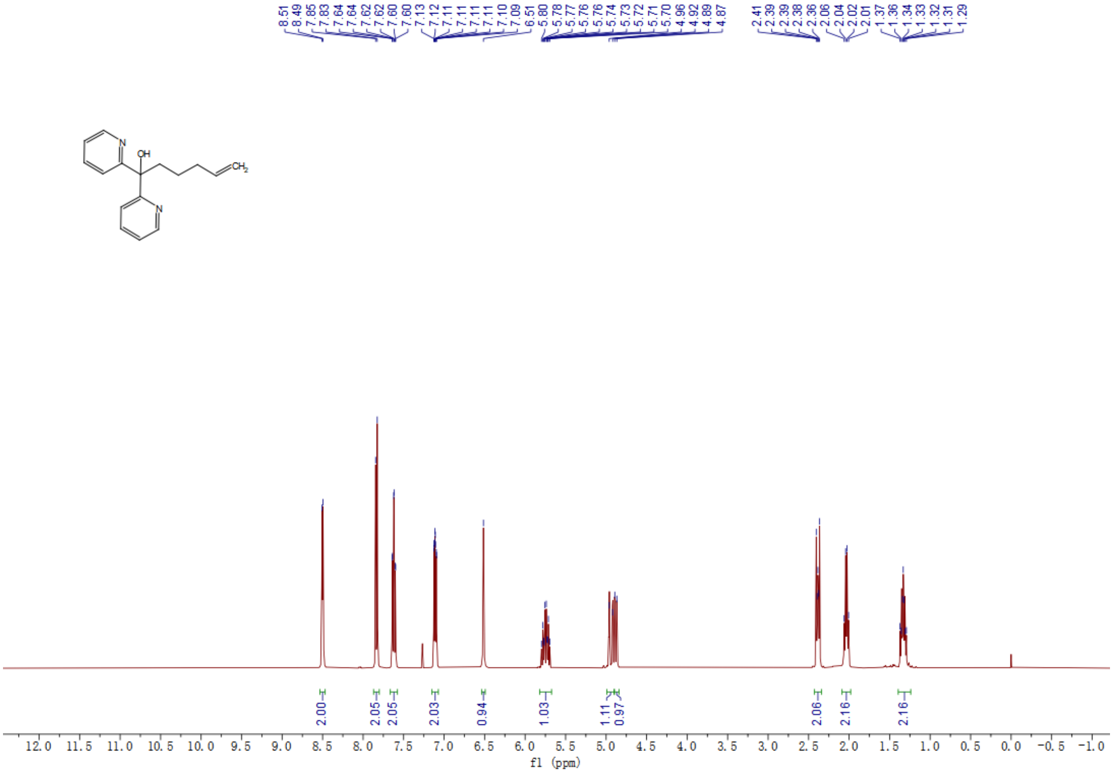

^{13}C NMR spectrum of **1h** in $CDCl_3$ (101 MHz)

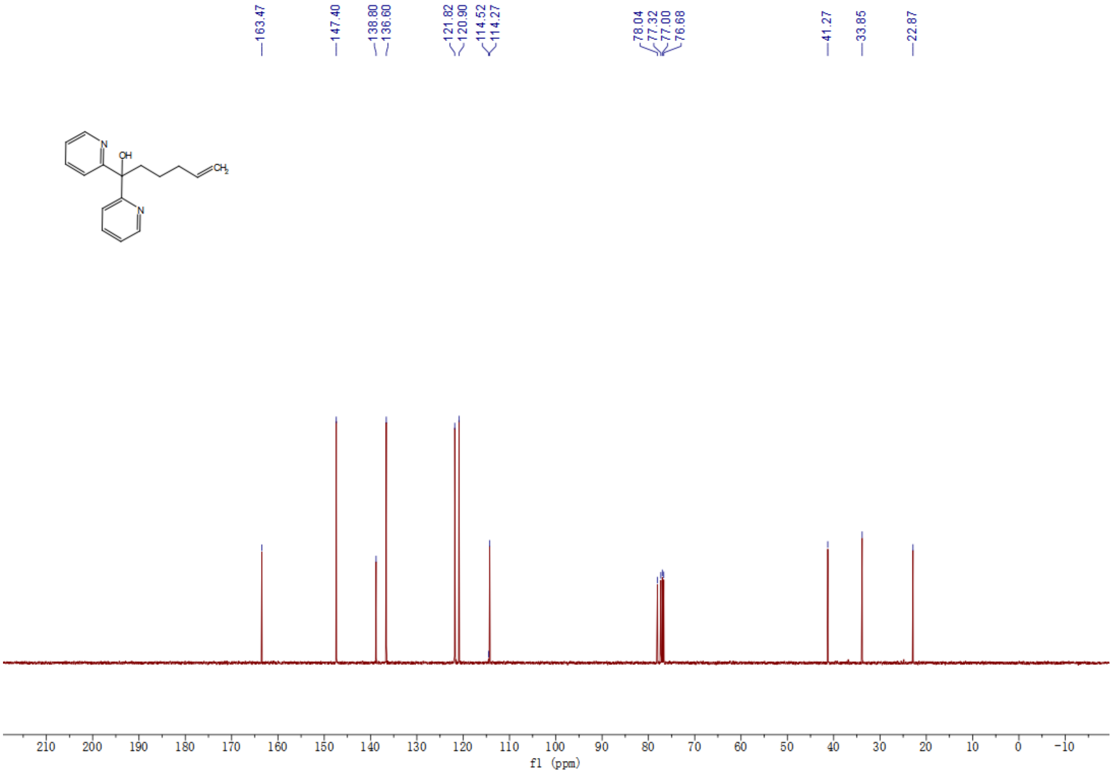

^{1}H NMR spectrum of **1i** in CDCl3 (400 MHz)

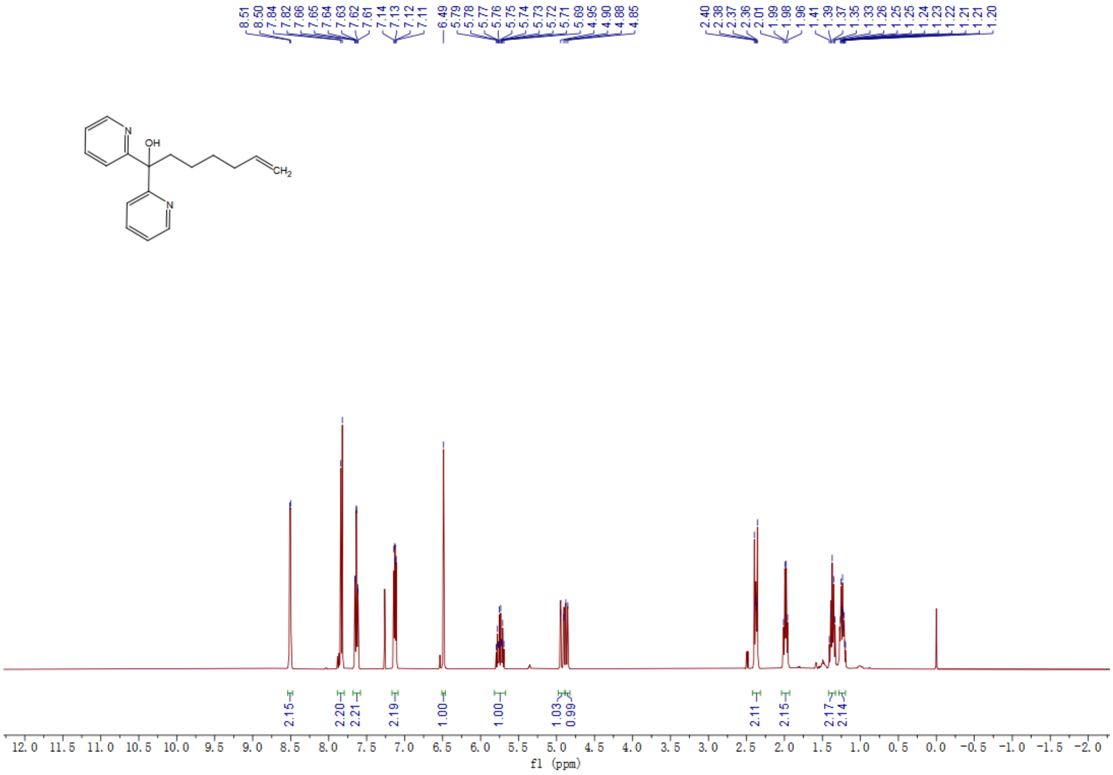

^{13}C NMR spectrum of **1i** in $CDCl_3$ (101 MHz)

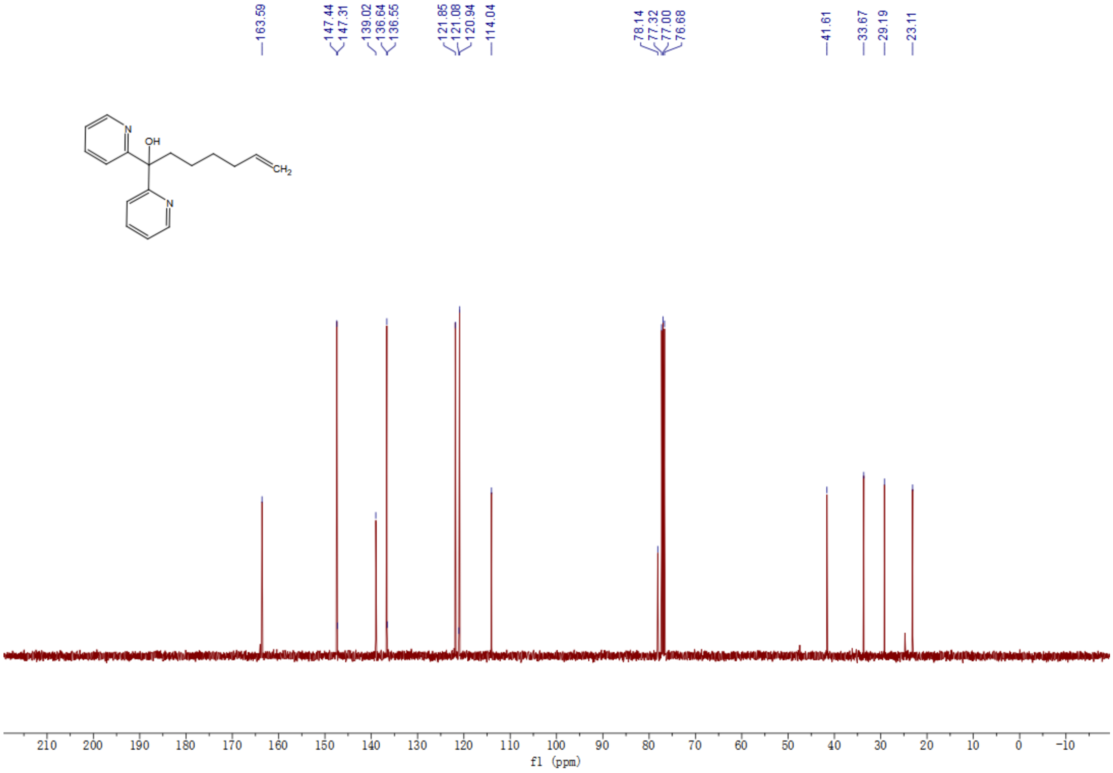

^{1}H NMR spectrum of **3aa** in CDCl3 (400 MHz)

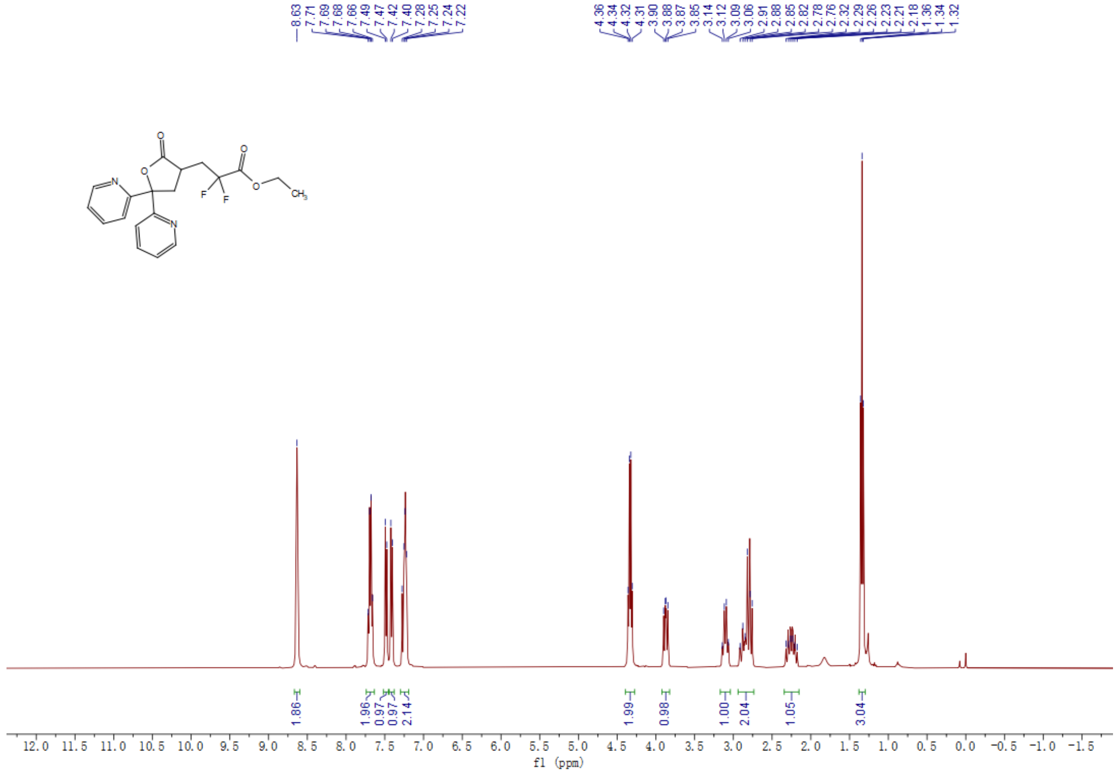

^{13}C NMR spectrum of **3aa** in $CDCl_3$ (101 MHz)

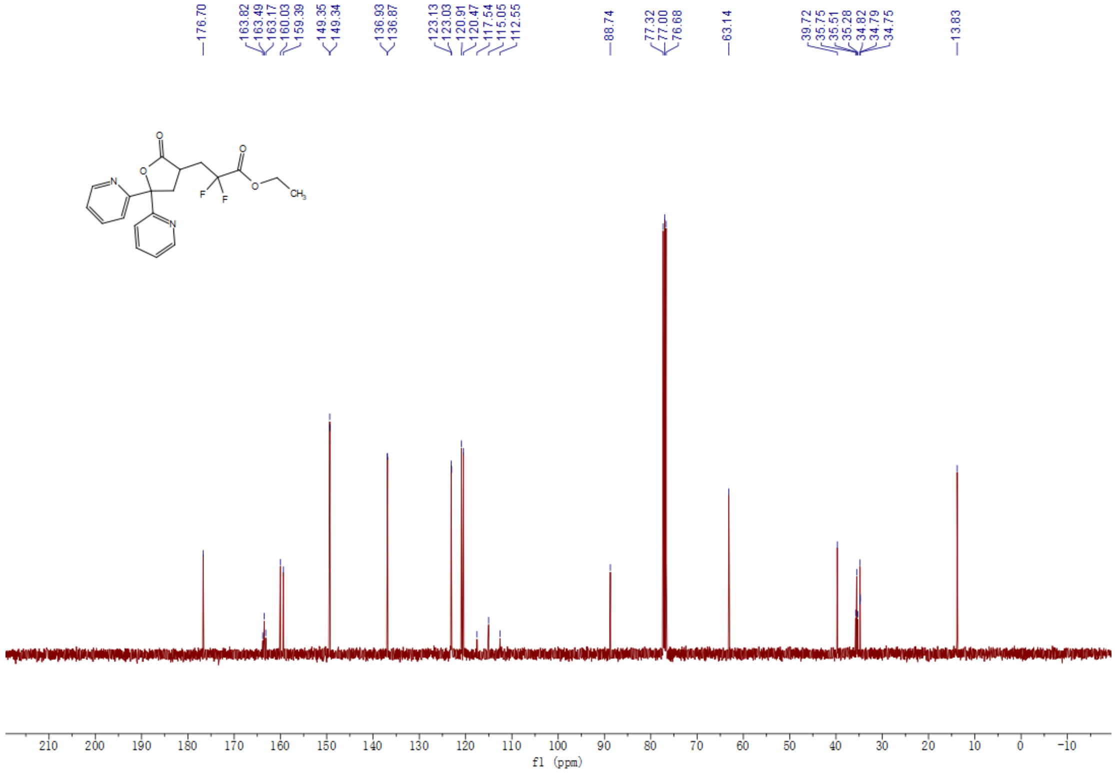

^{19}F NMR spectrum of **3aa** in $CDCl_3$ (376 MHz)

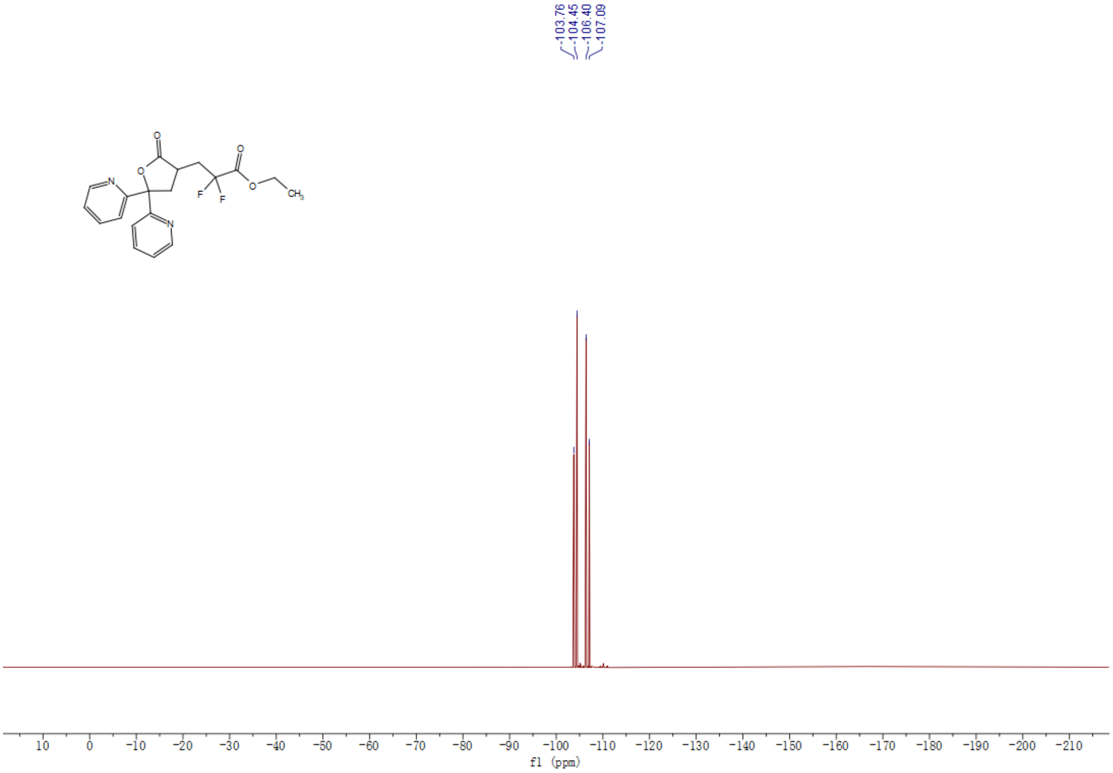

^{1}H NMR spectrum of **3ab** in CDCl3 (400 MHz)

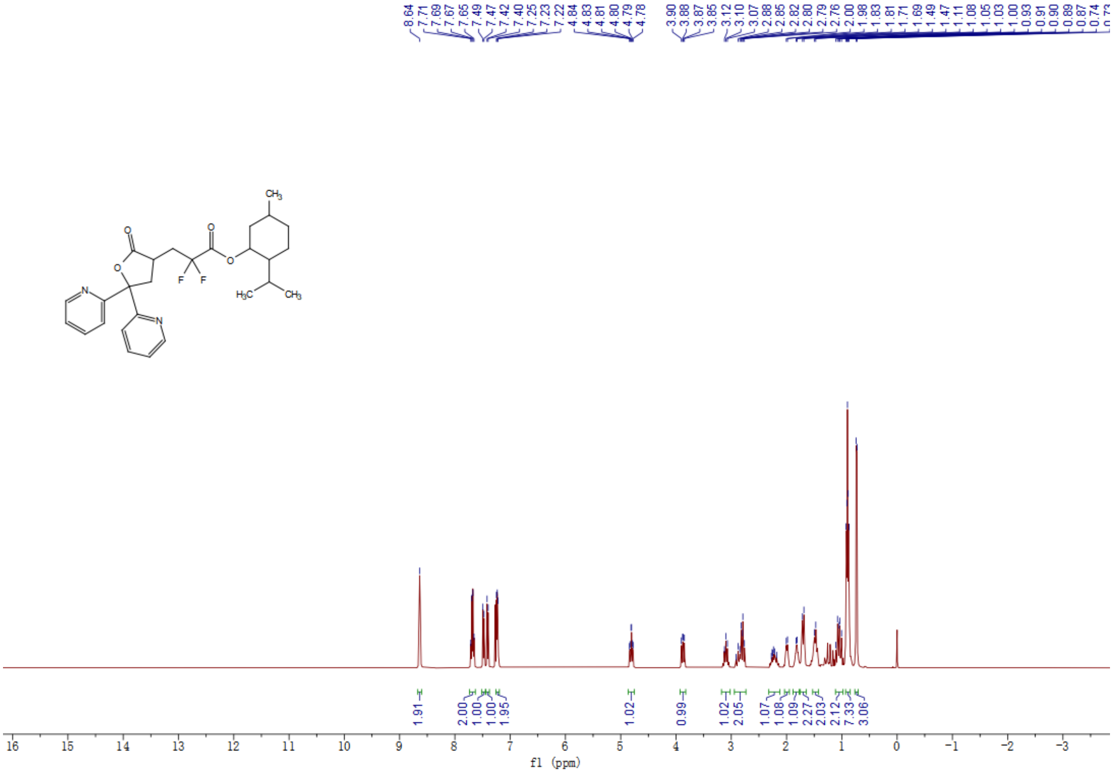

^{13}C NMR spectrum of **3ab** in $CDCl_3$ (101 MHz)

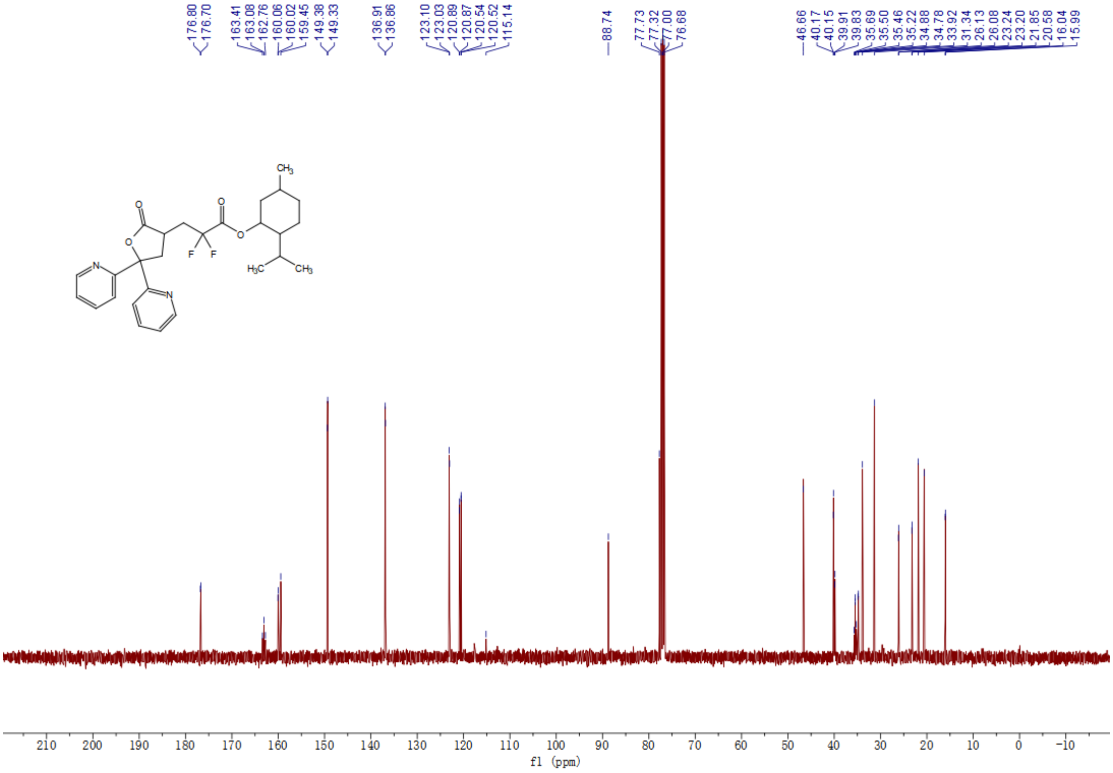

^{19}F NMR spectrum of **3ab** in $CDCl_3$ (376 MHz)

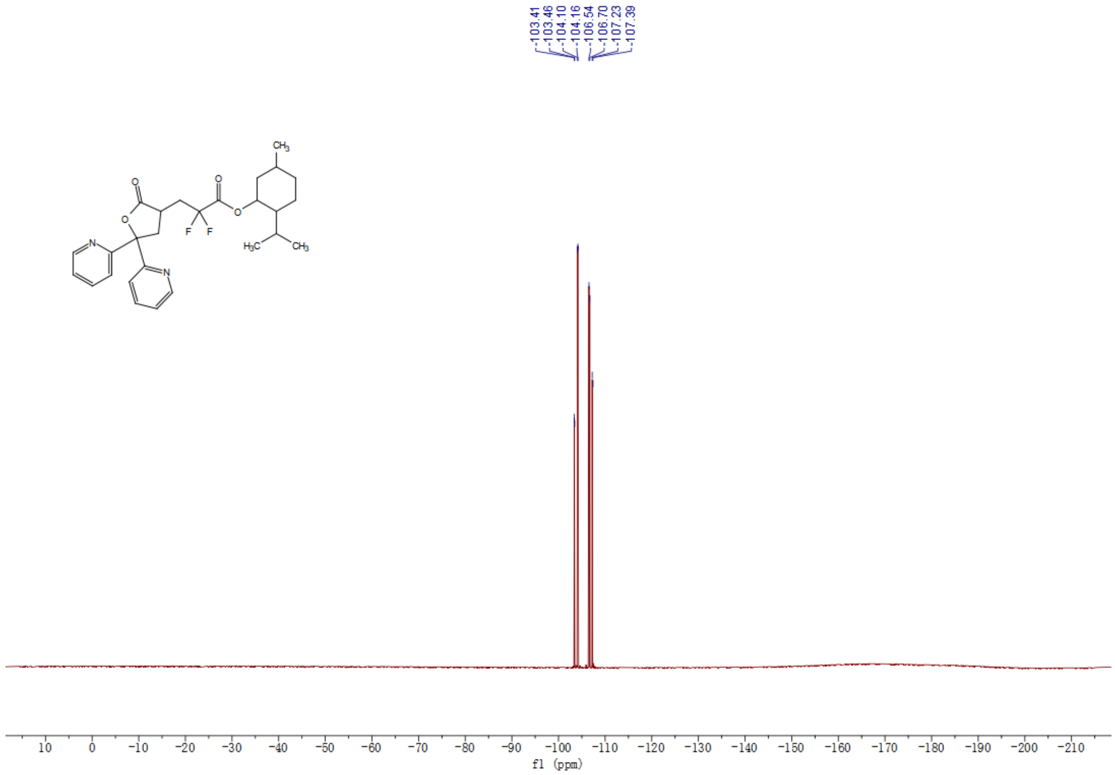

^{1}H NMR spectrum of **3ac** in CDCl3 (400 MHz)

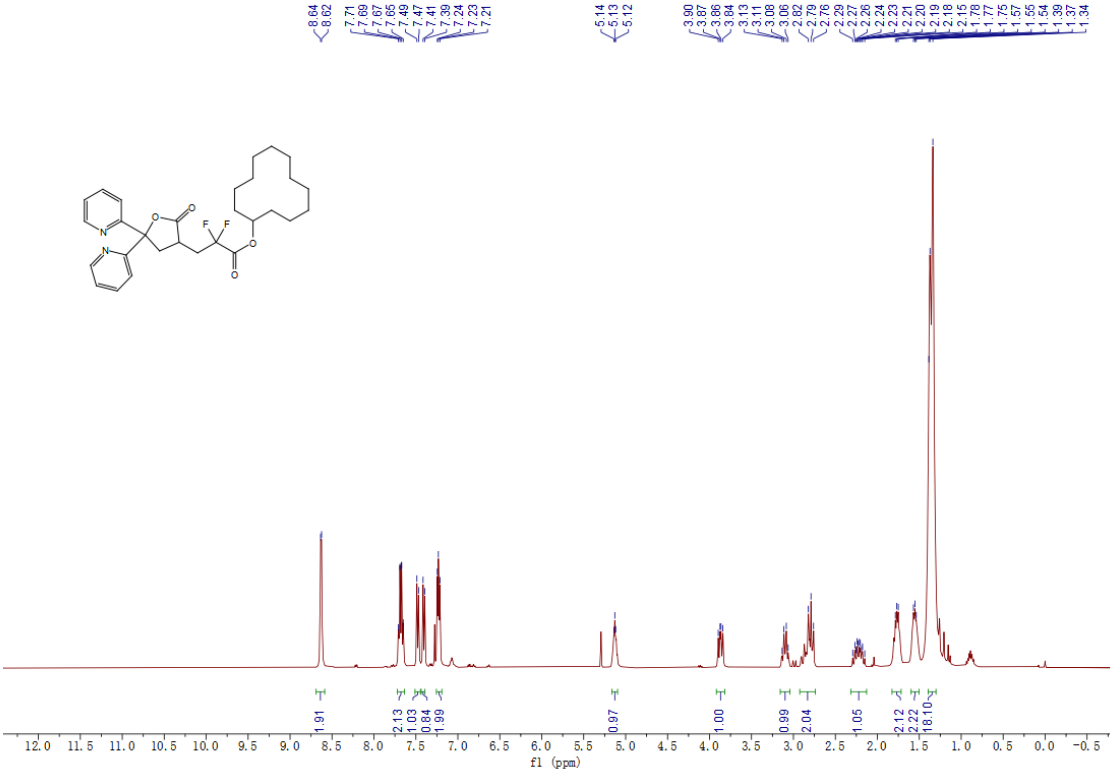

^{13}C NMR spectrum of **3ac** in $CDCl_3$ (101 MHz)

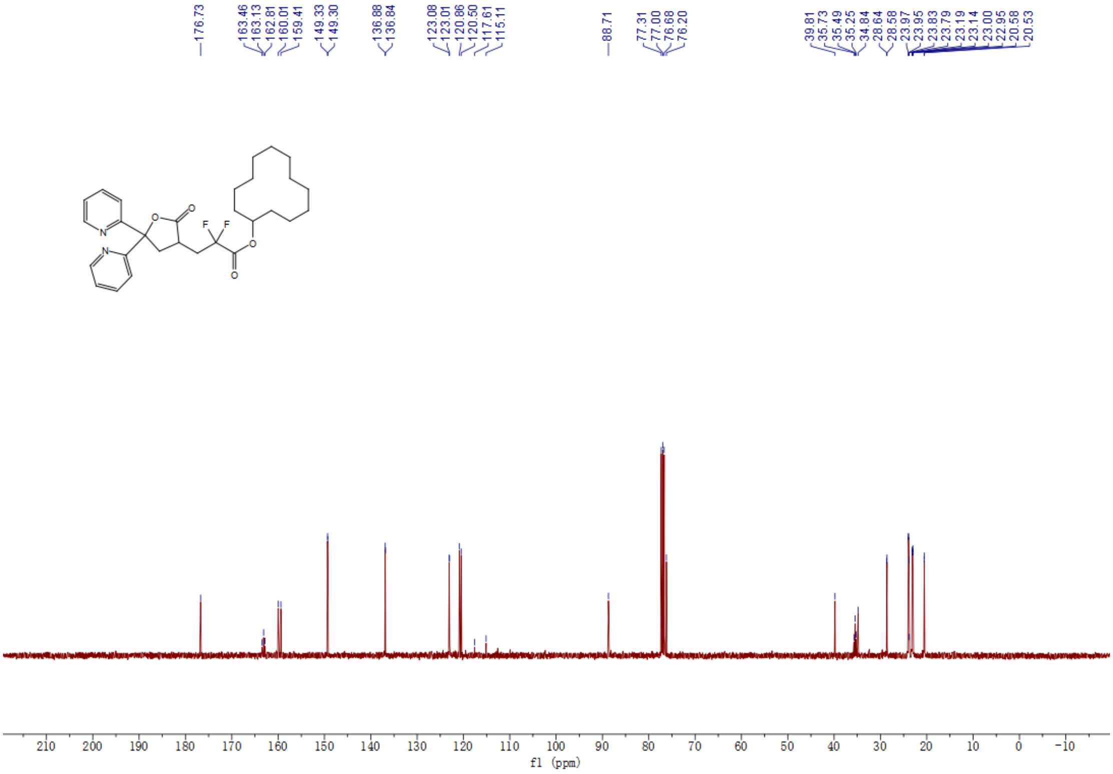

^{19}F NMR spectrum of **3ac** in $CDCl_3$ (376 MHz)

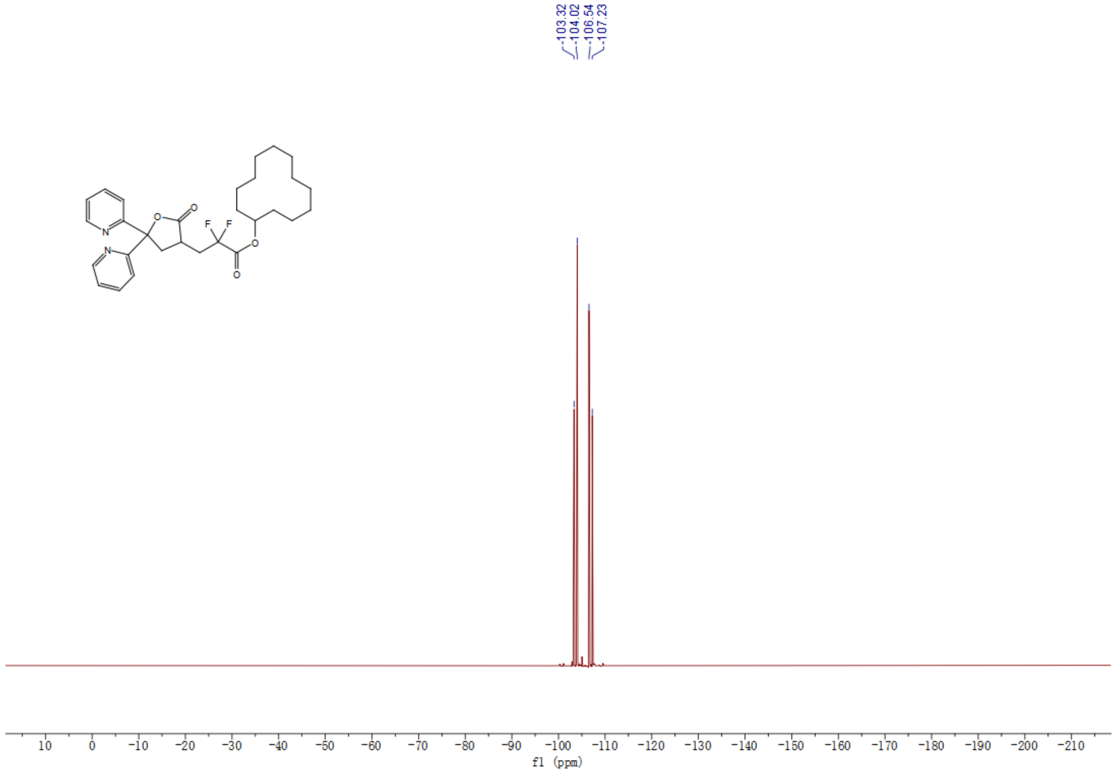

^{1}H NMR spectrum of **3ad** in CDCl3 (400 MHz)

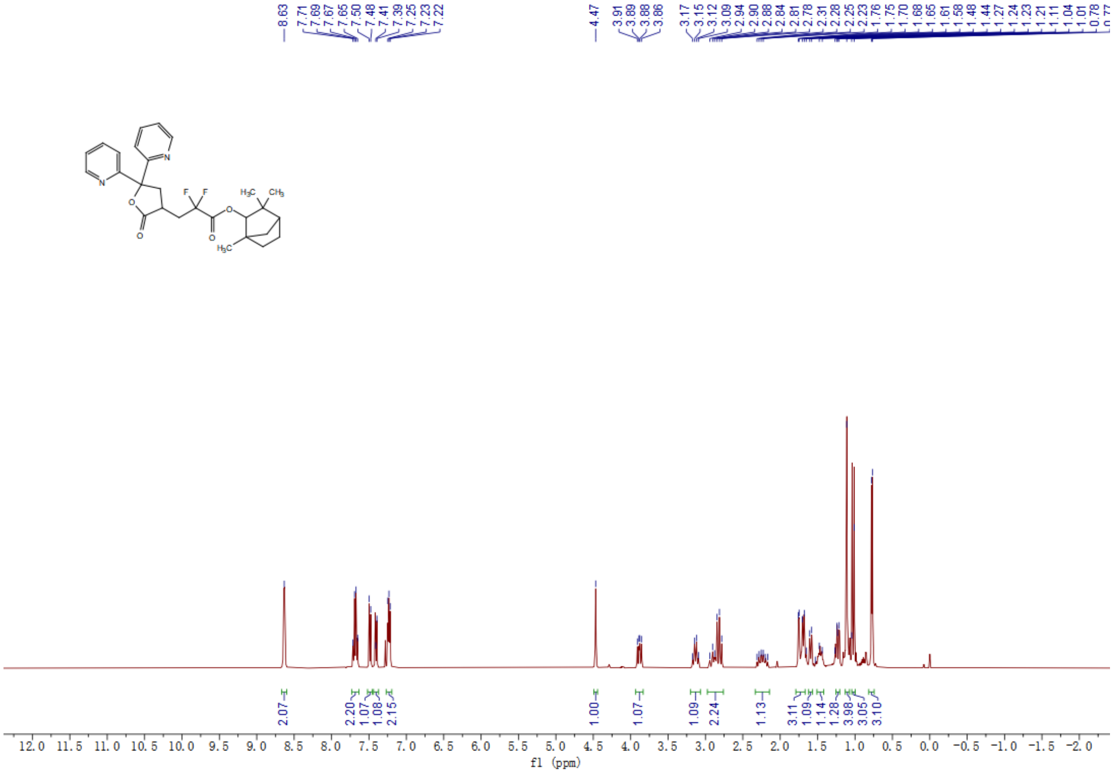

^{13}C NMR spectrum of **3ae** in $CDCl_3$ (101 MHz)

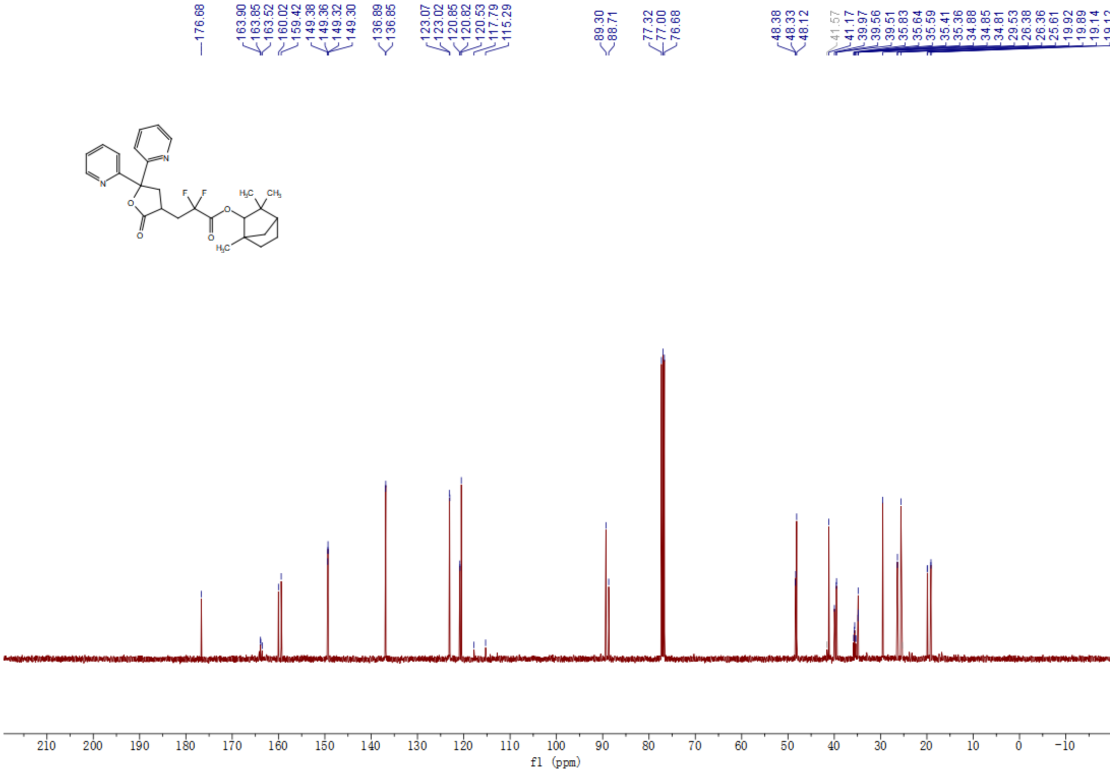

^{19}F NMR spectrum of **3ae** in $CDCl_3$ (376 MHz)

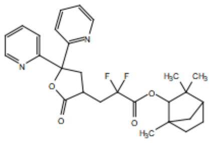

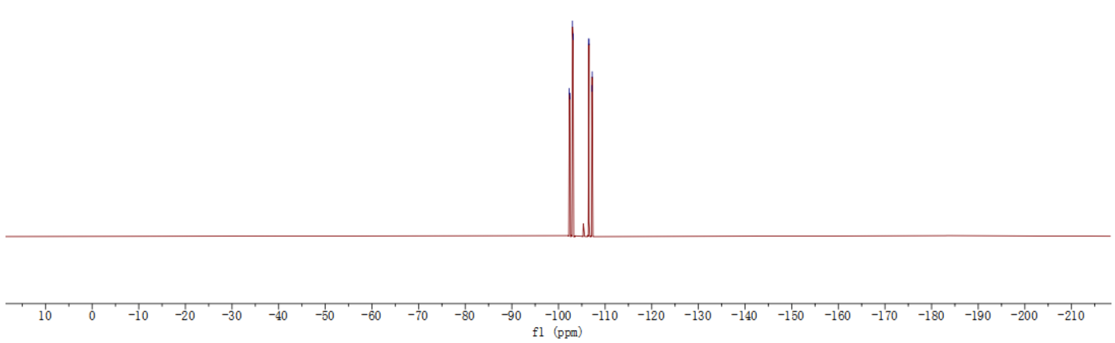

^{1}H NMR spectrum of **3ae** in CDCl3 (400 MHz)

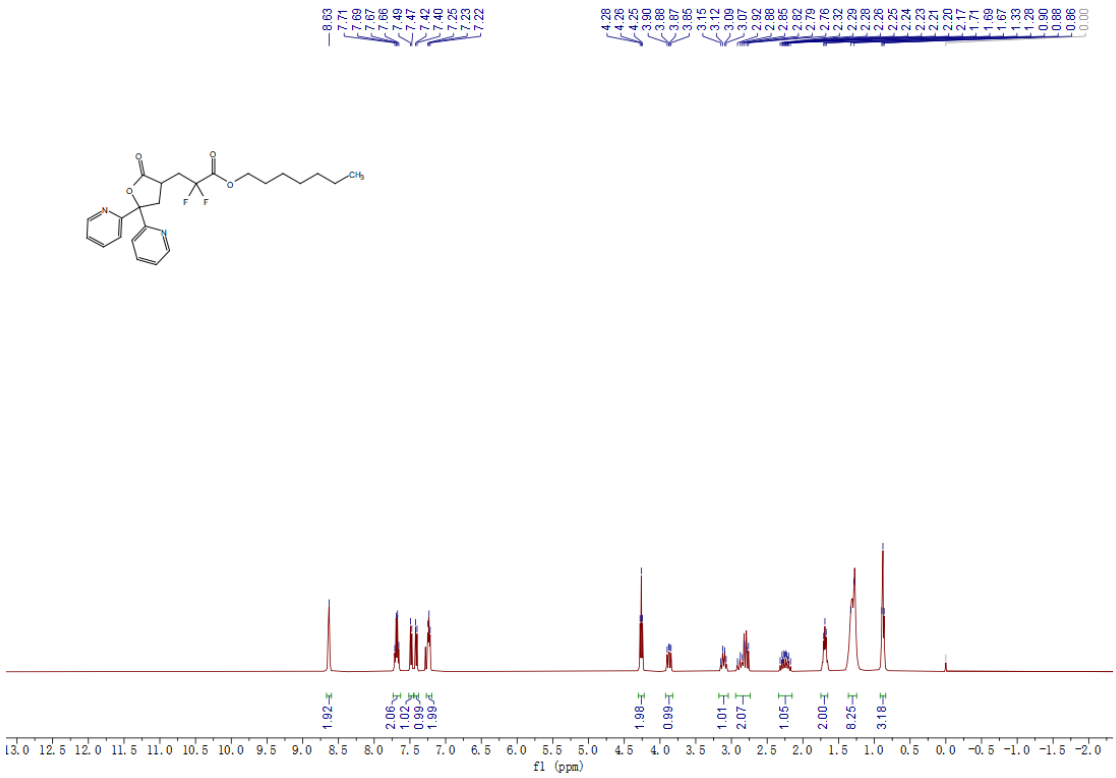

^{13}C NMR spectrum of **3ae** in $CDCl_3$ (101 MHz)

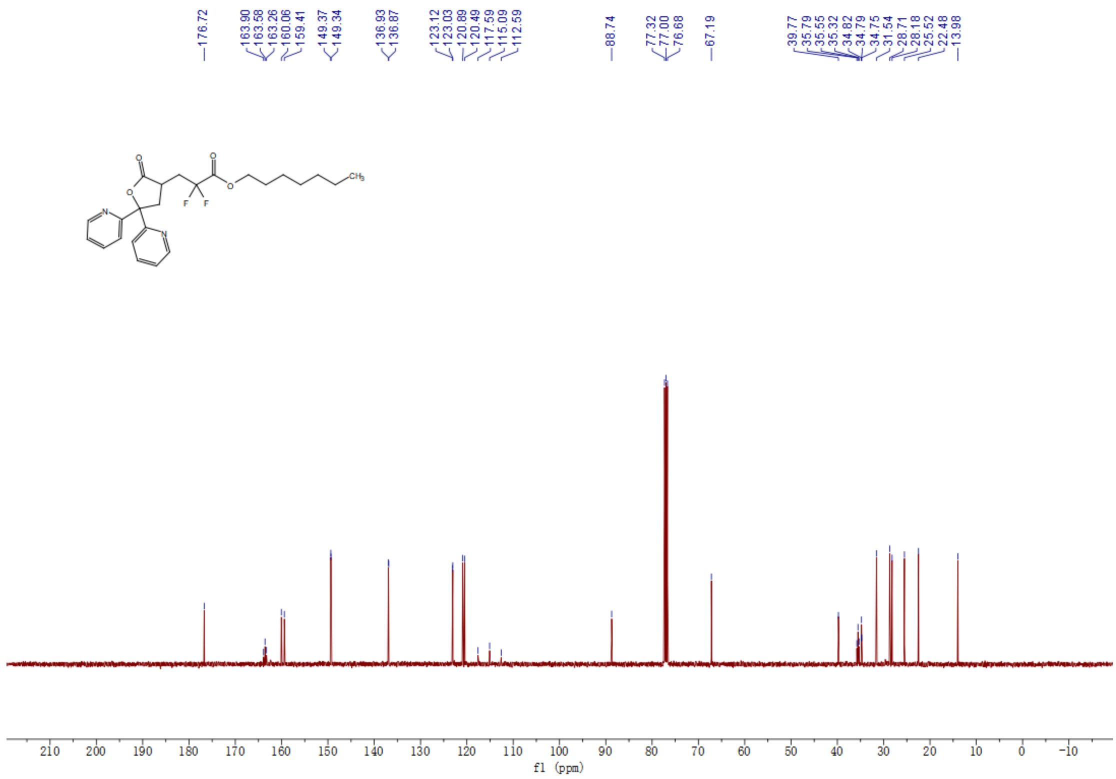

^{19}F NMR spectrum of **3ae** in $CDCl_3$ (376 MHz)

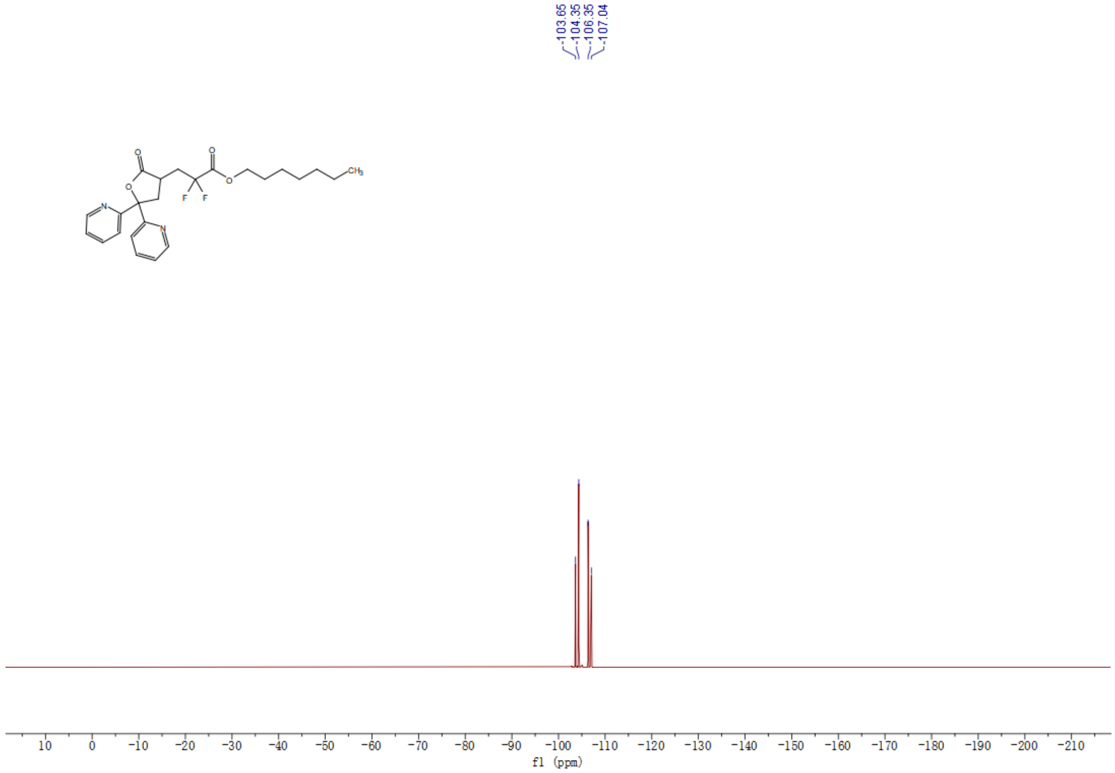

^{1}H NMR spectrum of **3af** in CDCl3 (400 MHz)

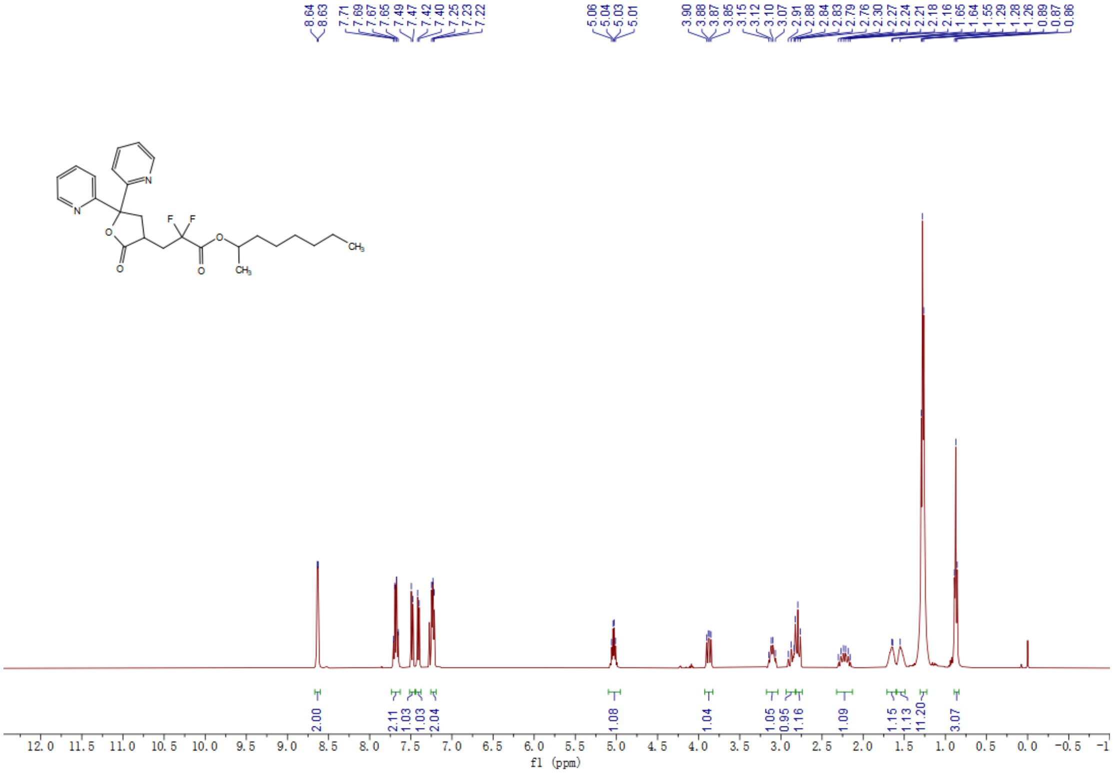

^{13}C NMR spectrum of **3af** in $CDCl_3$ (101 MHz)

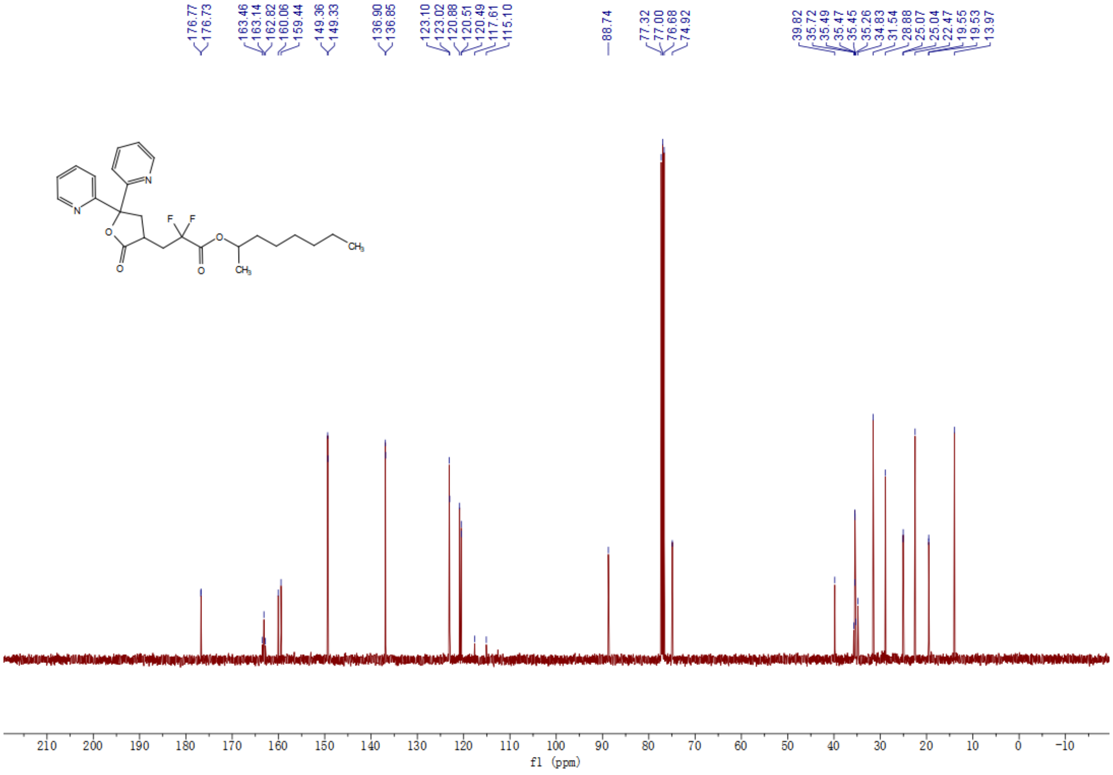

^{19}F NMR spectrum of **3af** in $CDCl_3$ (376 MHz)

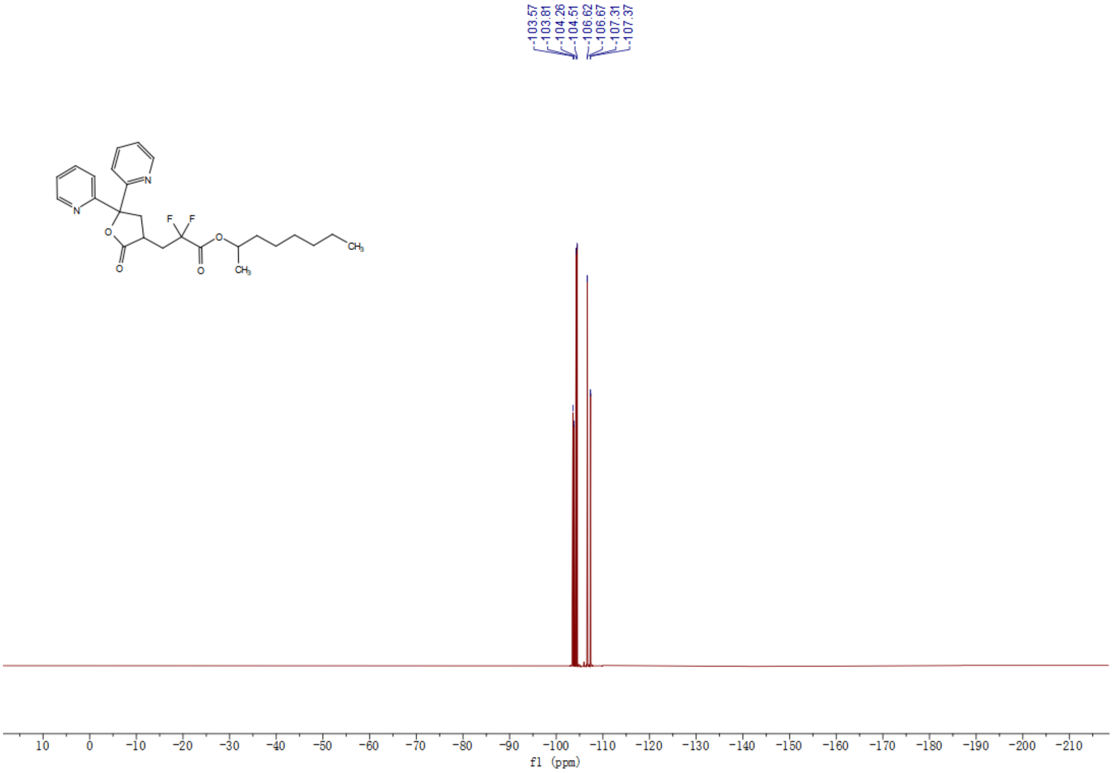

^{1}H NMR spectrum of **3ag** in $CDCl_3$ (400 MHz)

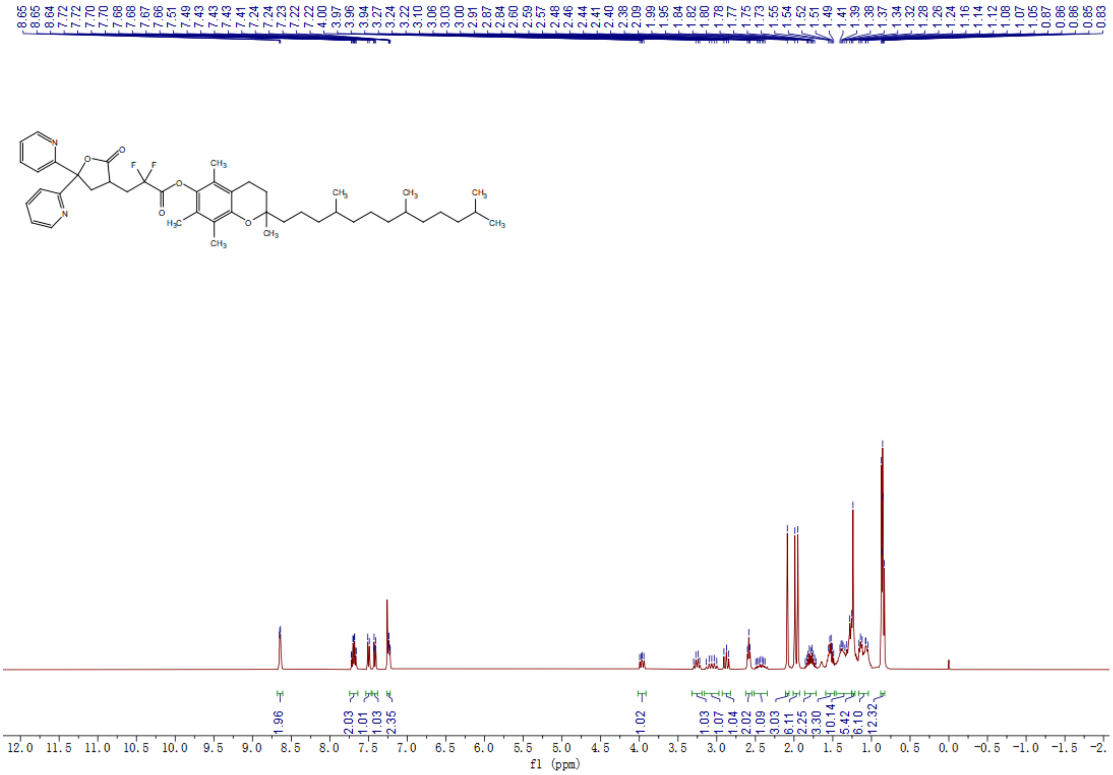

^{13}C NMR spectrum of **3ag** in $CDCl_3$ (101 MHz)

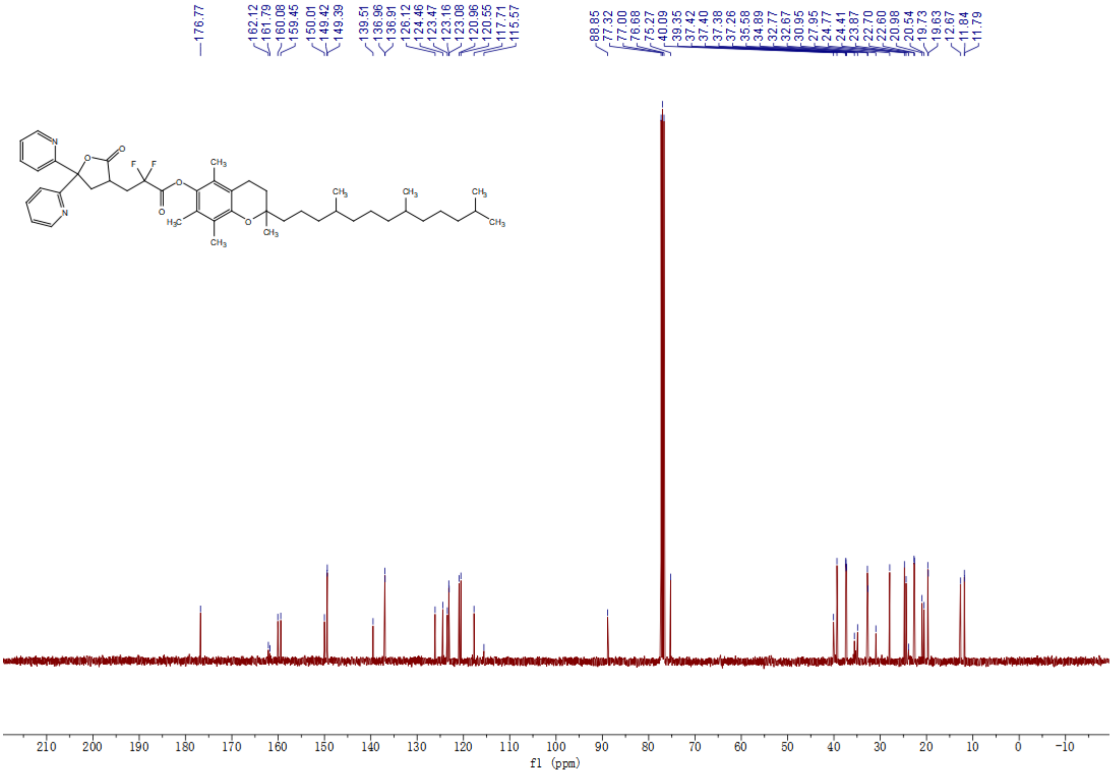

^{19}F NMR spectrum of **3ag** in $CDCl_3$ (376 MHz)

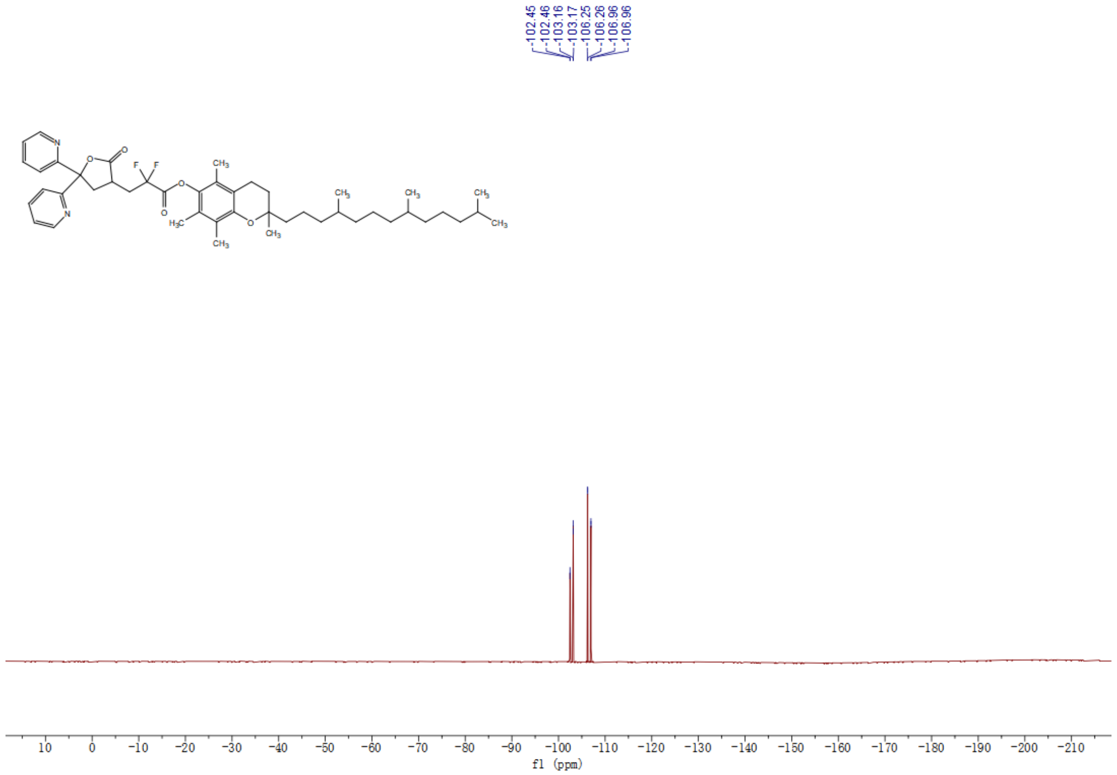

^{1}H NMR spectrum of **3ah** in $CDCl_3$ (400 MHz)

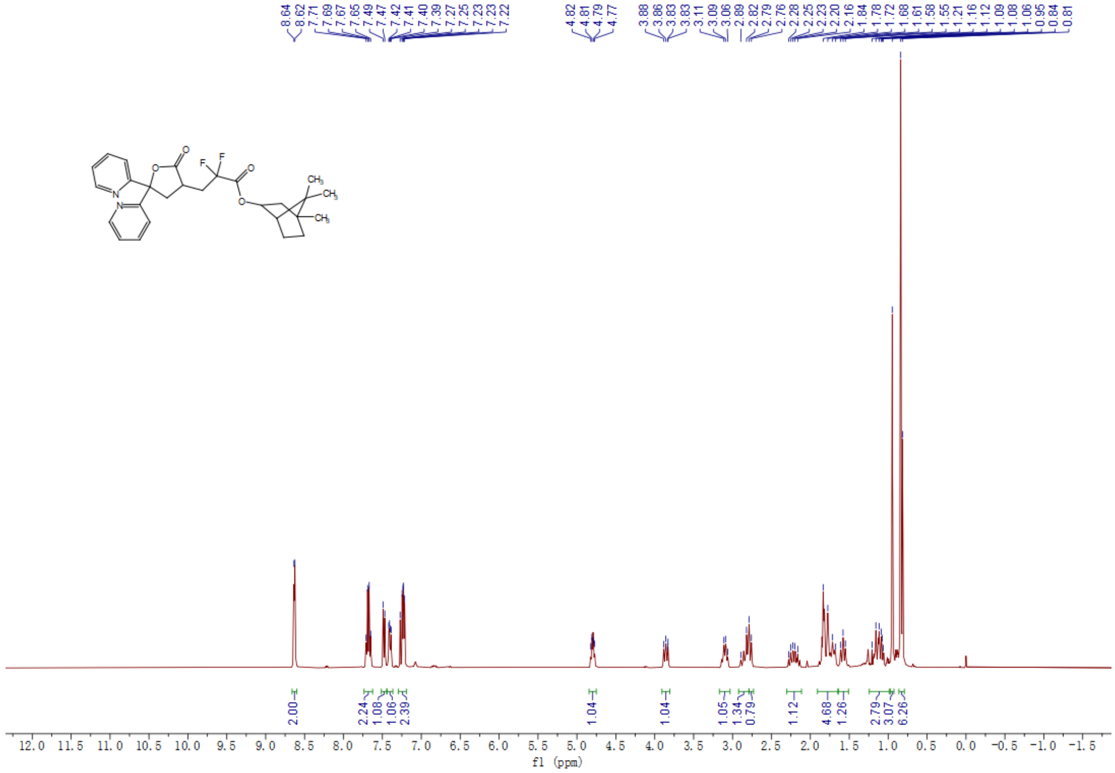

^{13}C NMR spectrum of **3ah** in $CDCl_3$ (101 MHz)

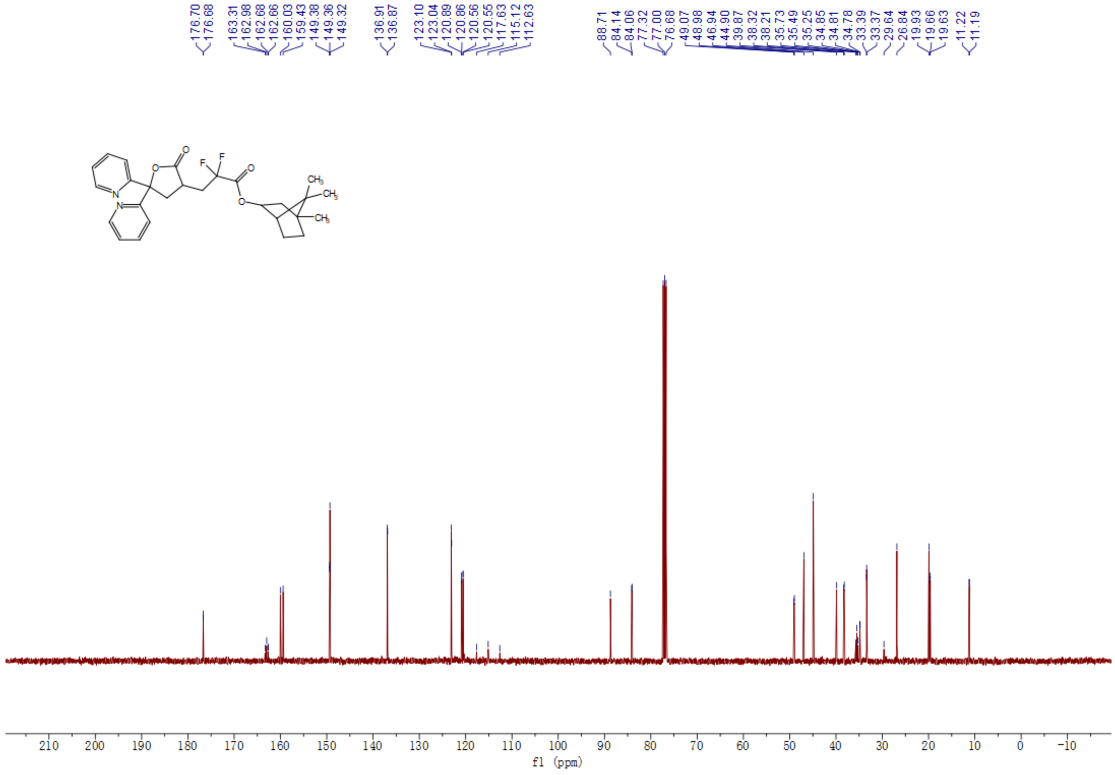

^{19}F NMR spectrum of **3ah** in $CDCl_3$ (376 MHz)

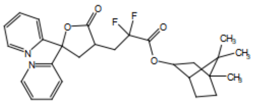

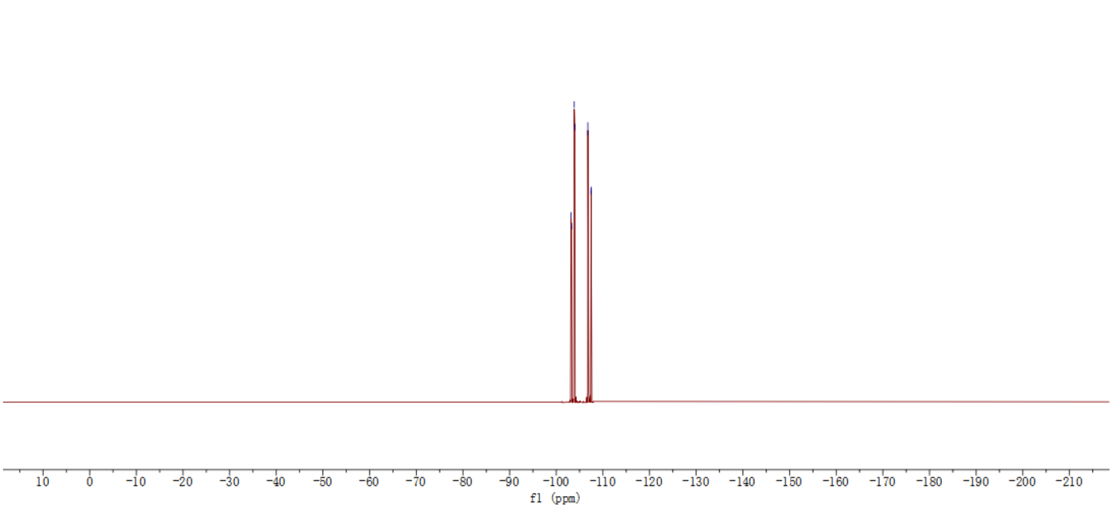

^{1}H NMR spectrum of **3ai** in $CDCl_3$ (400 MHz)

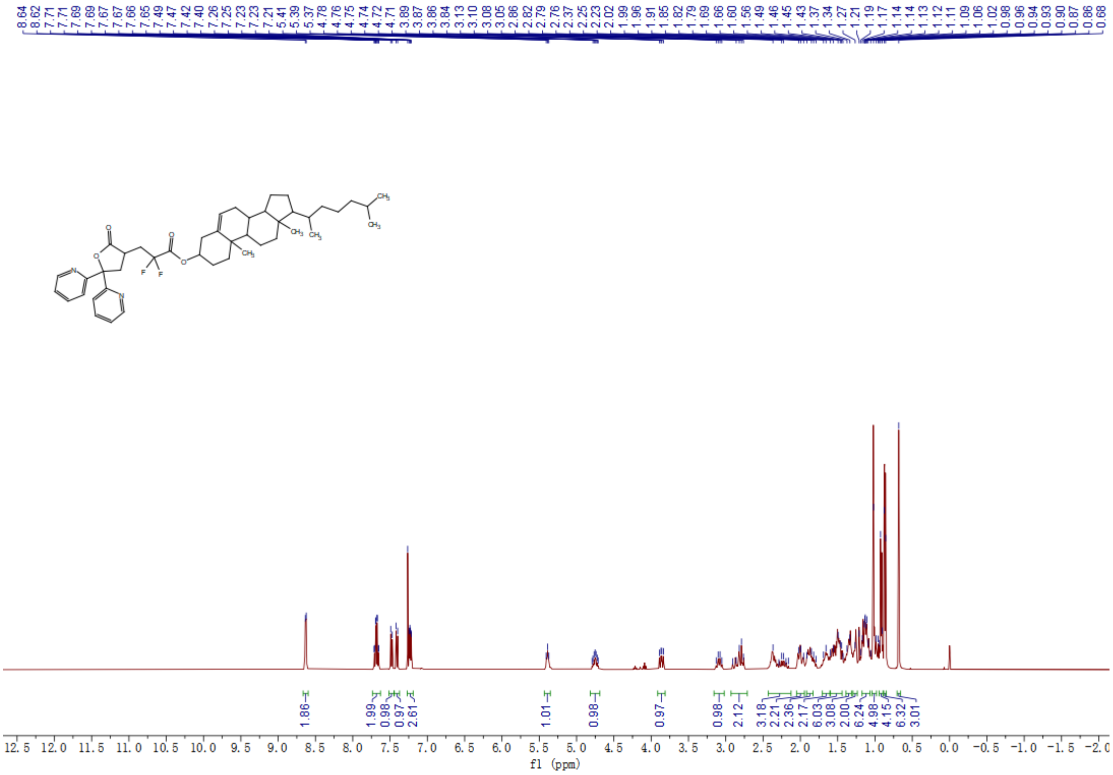

^{13}C NMR spectrum of **3ai** in $CDCl_3$ (101 MHz)

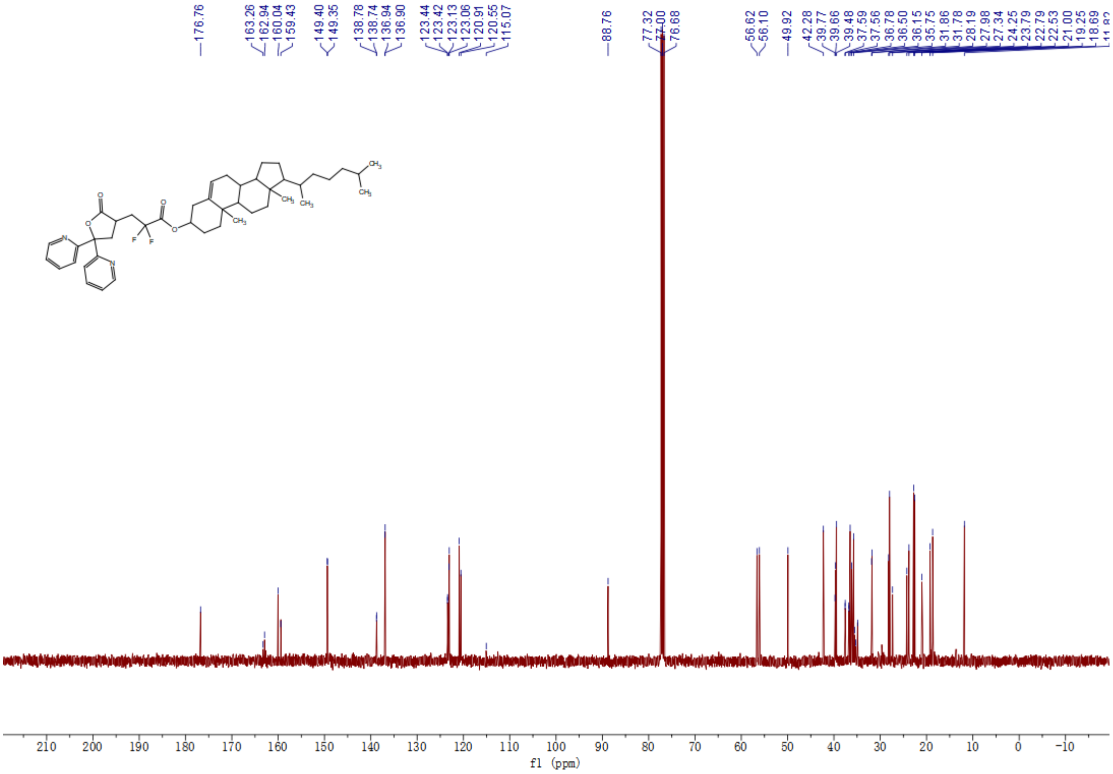

^{19}F NMR spectrum of **3ai** in $CDCl_3$ (376 MHz)

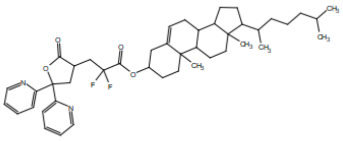

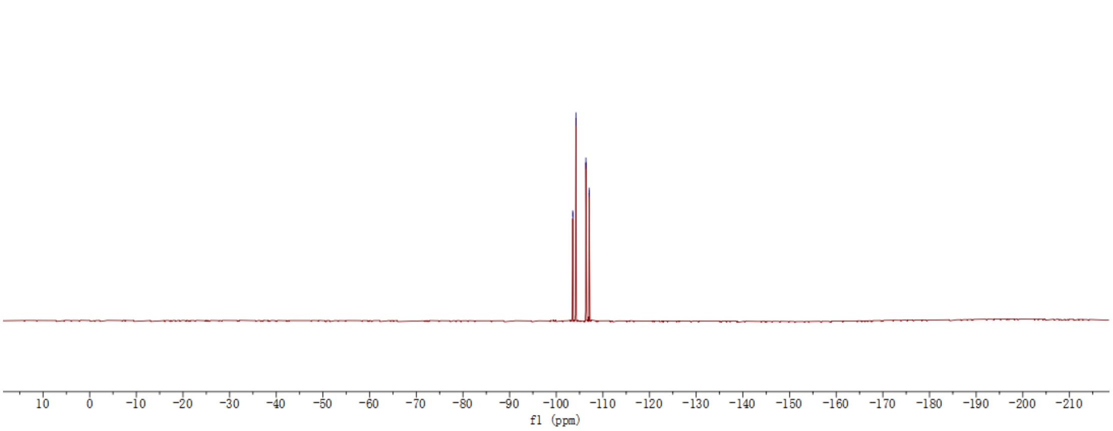

^{1}H NMR spectrum of **3aj** in $CDCl_3$ (400 MHz)

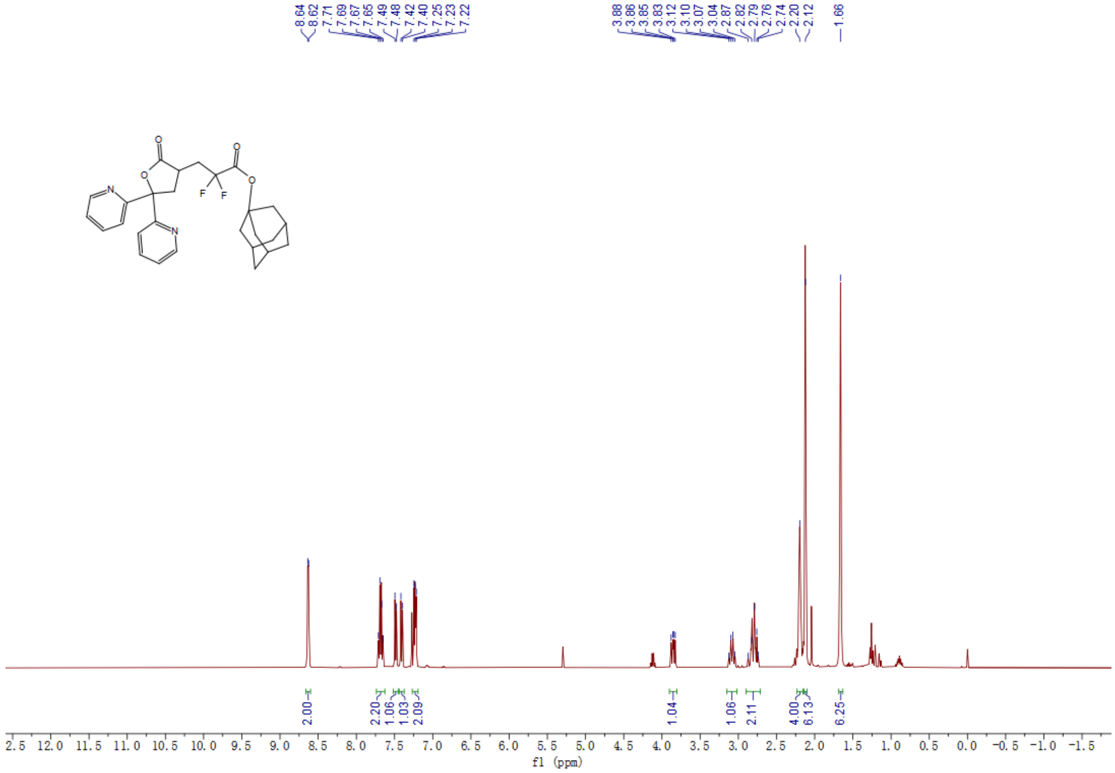

^{13}C NMR spectrum of **3aj** in $CDCl_3$ (101 MHz)

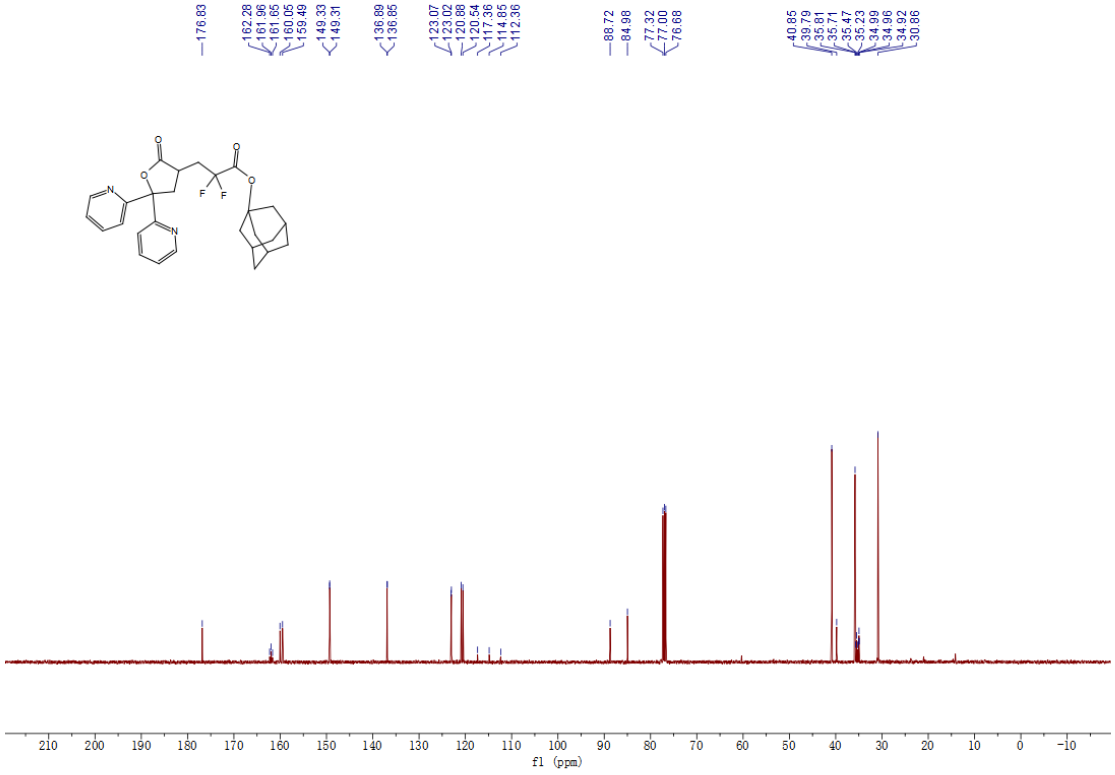

^{19}F NMR spectrum of **3aj** in $CDCl_3$ (376 MHz)

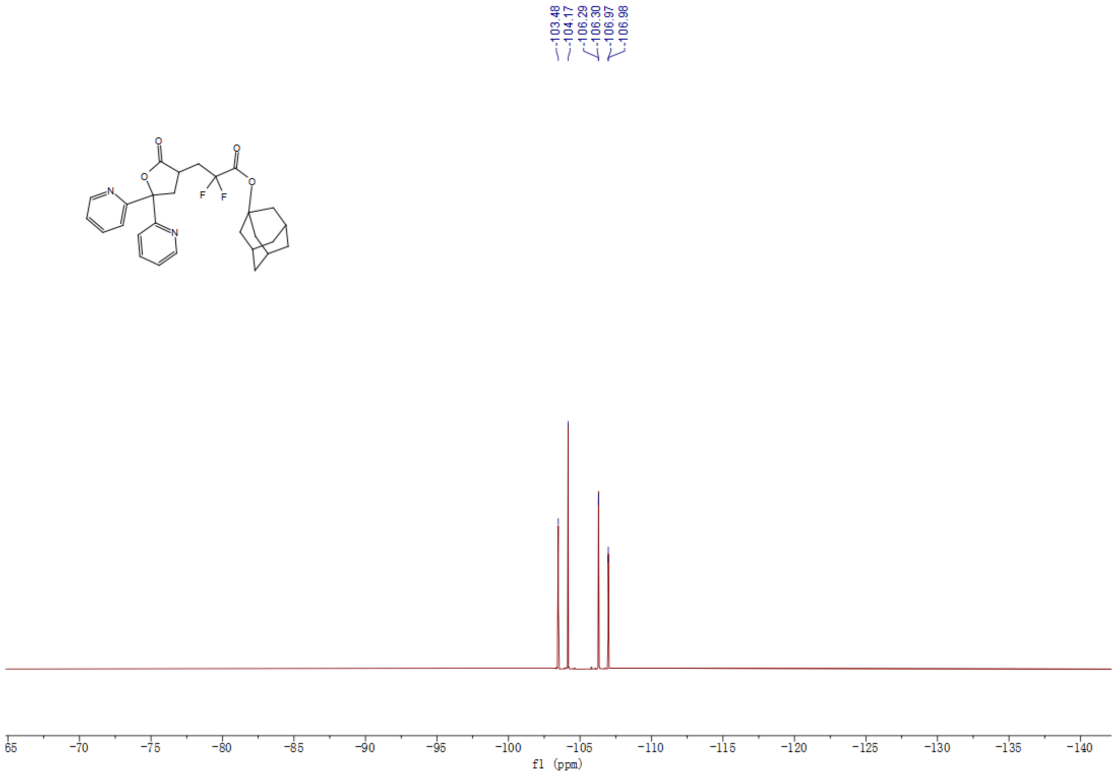

^{1}H NMR spectrum of **3ak** in $CDCl_3$ (400 MHz)

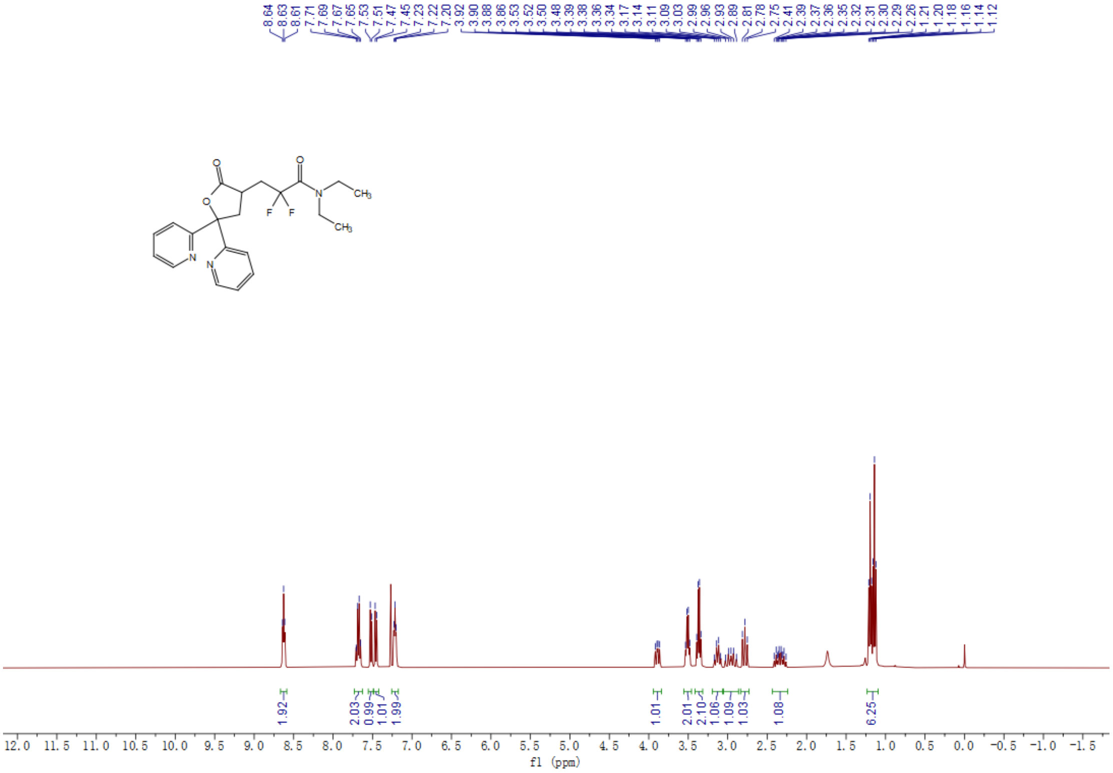

^{13}C NMR spectrum of **3ak** in $CDCl_3$ (101 MHz)

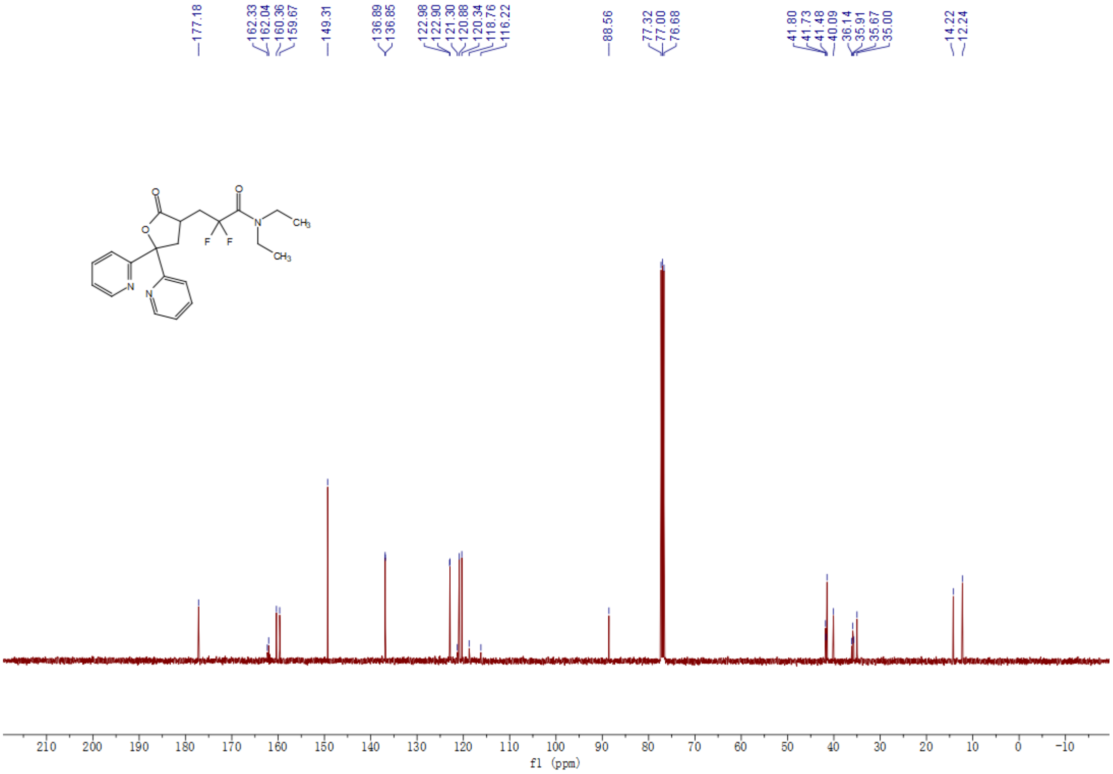

^{19}F NMR spectrum of **3ak** in $CDCl_3$ (376 MHz)

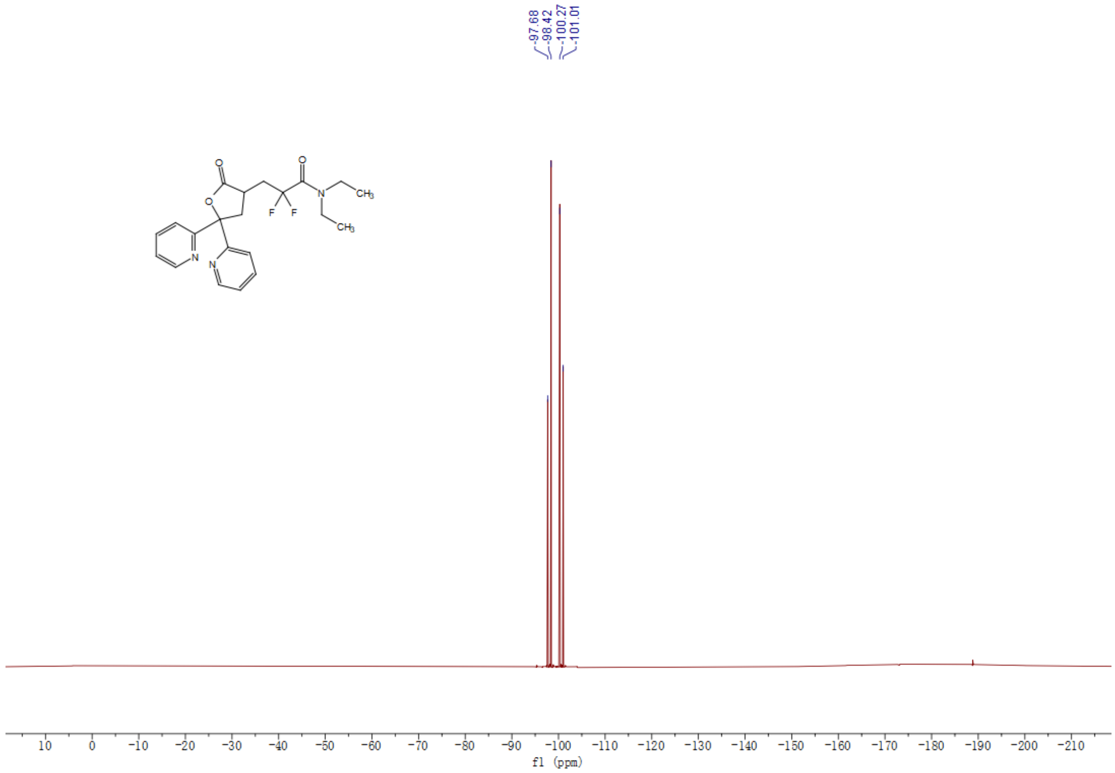

^{1}H NMR spectrum of **3al** in $CDCl_3$ (400 MHz)

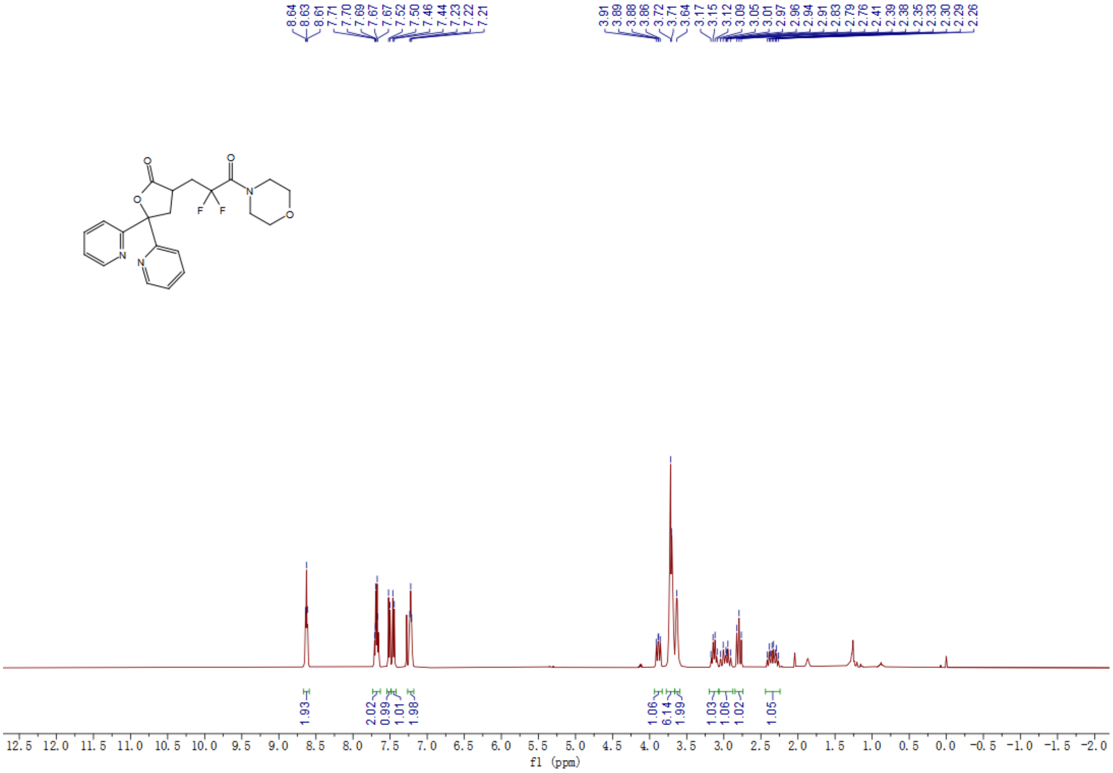

^{13}C NMR spectrum of **3al** in $CDCl_3$ (101 MHz)

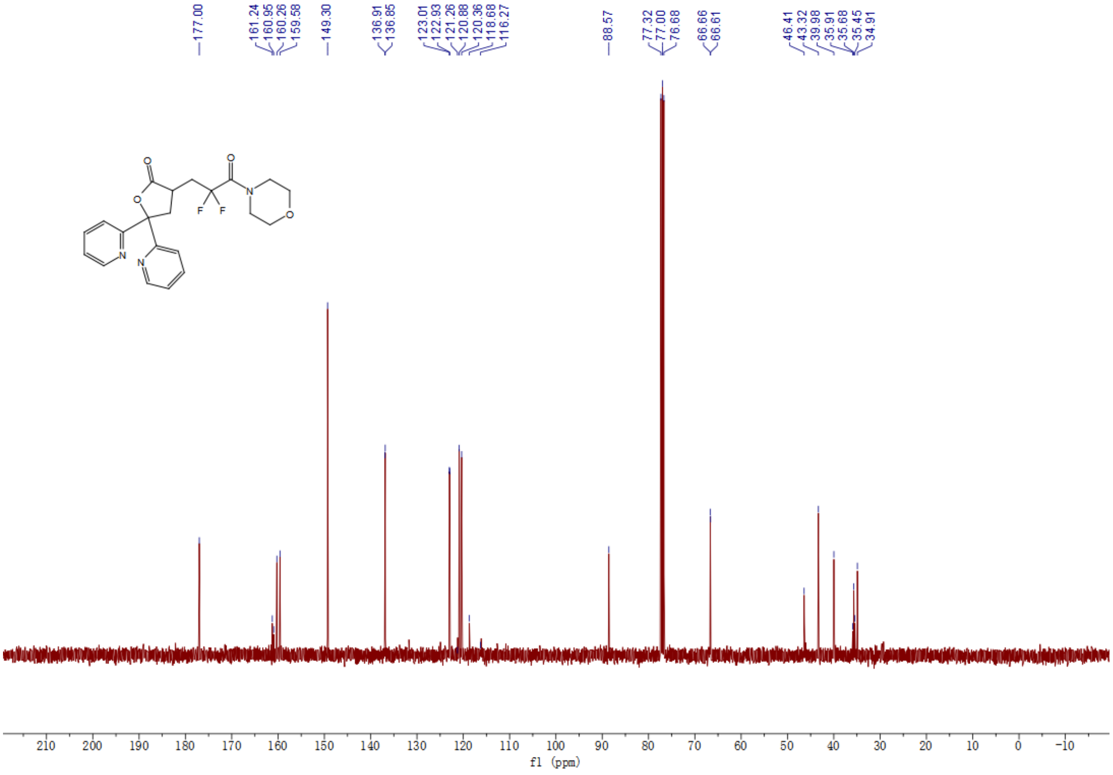

^{19}F NMR spectrum of **3al** in $CDCl_3$ (376 MHz)

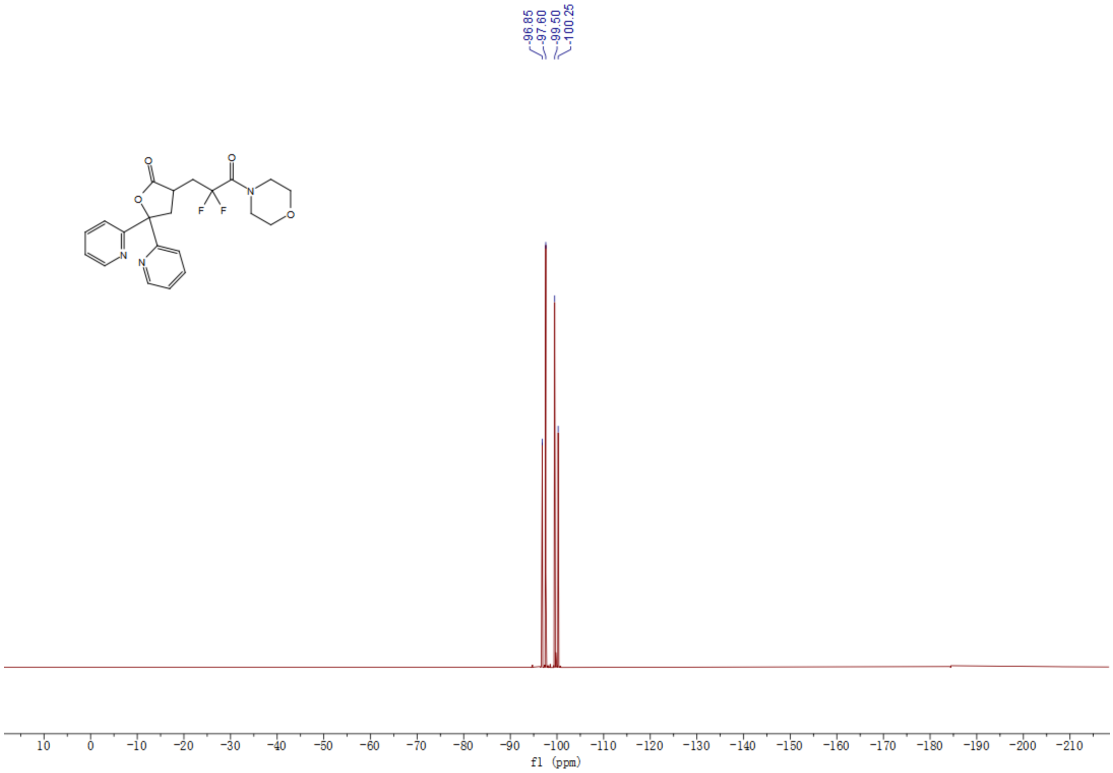

^{1}H NMR spectrum of **3am** in $CDCl_3$ (400 MHz)

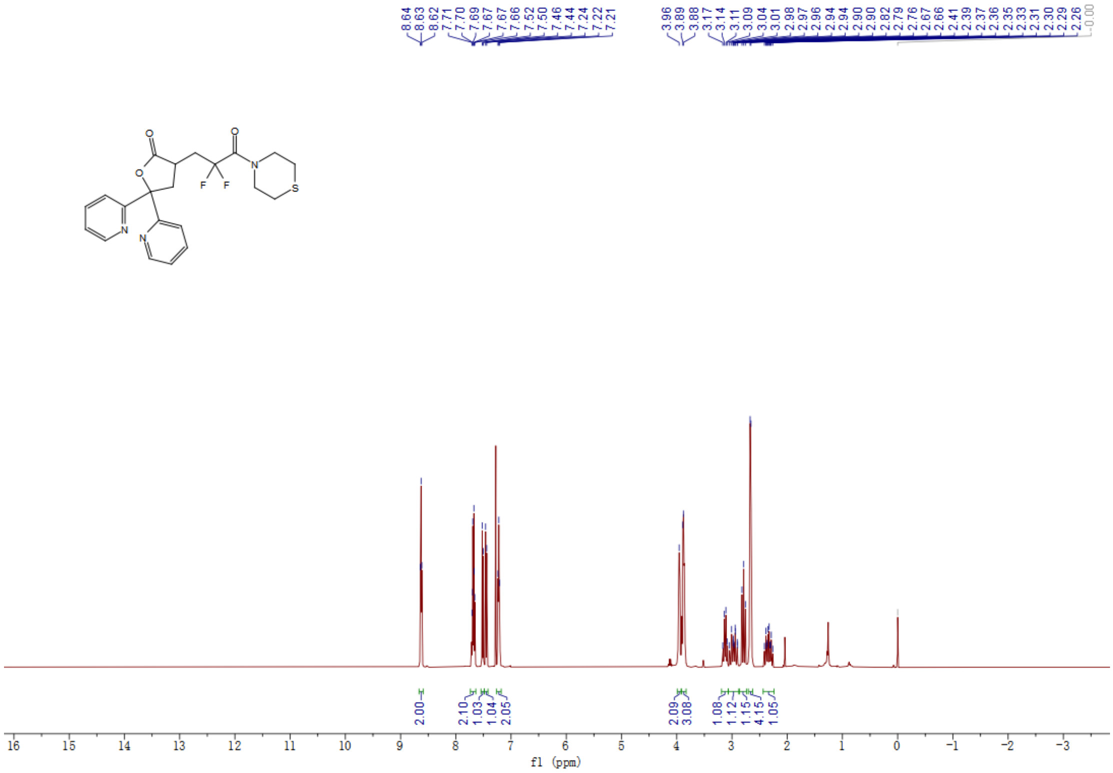

^{13}C NMR spectrum of **3am** in $CDCl_3$ (101 MHz)

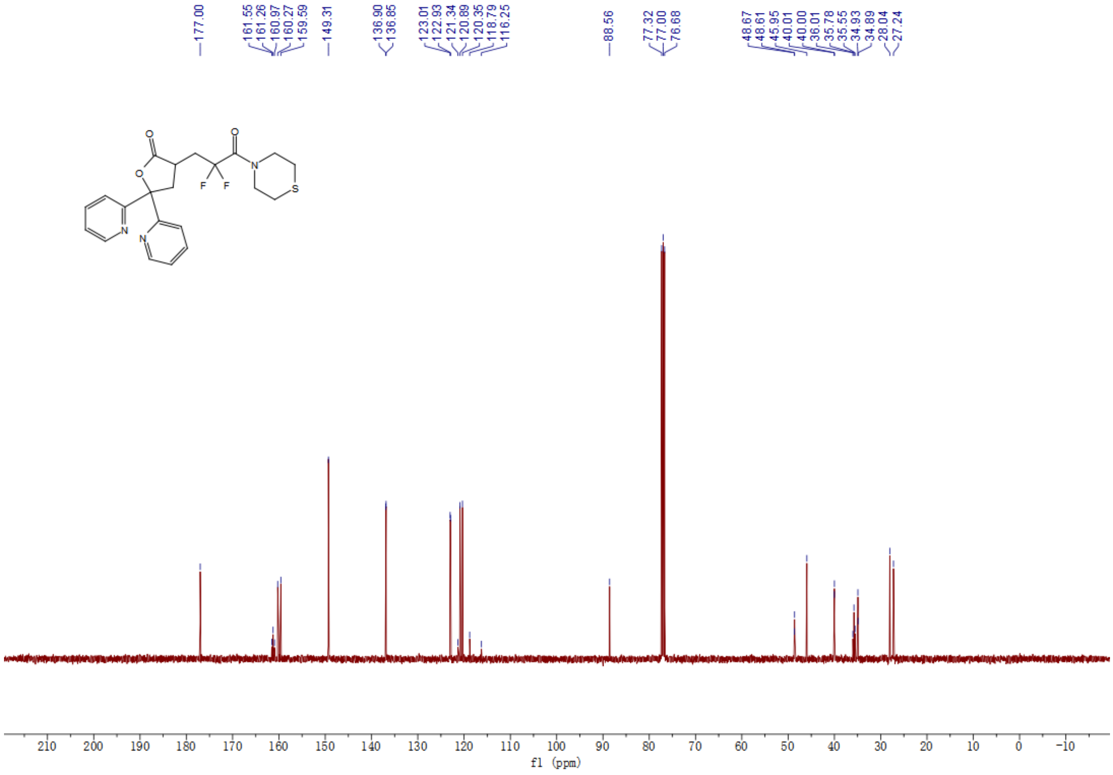

^{19}F NMR spectrum of **3am** in $CDCl_3$ (376 MHz)

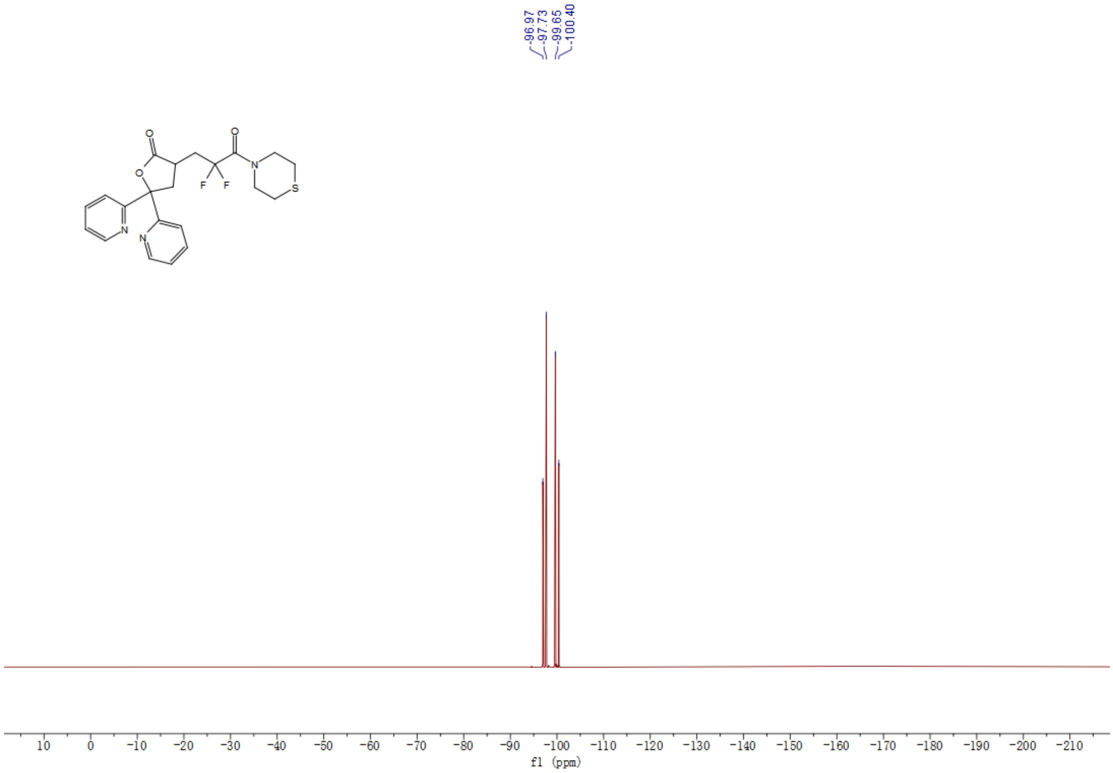

^{1}H NMR spectrum of **3an** in $CDCl_3$ (400 MHz)

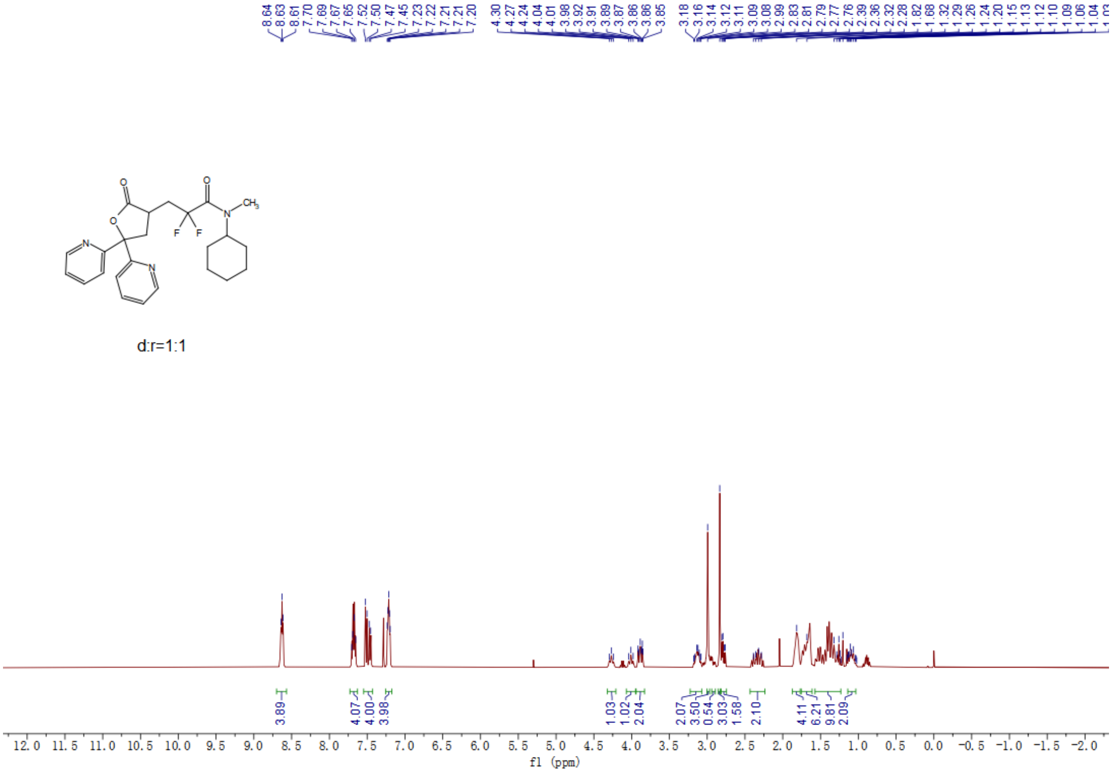

^{13}C NMR spectrum of **3an** in $CDCl_3$ (101 MHz)

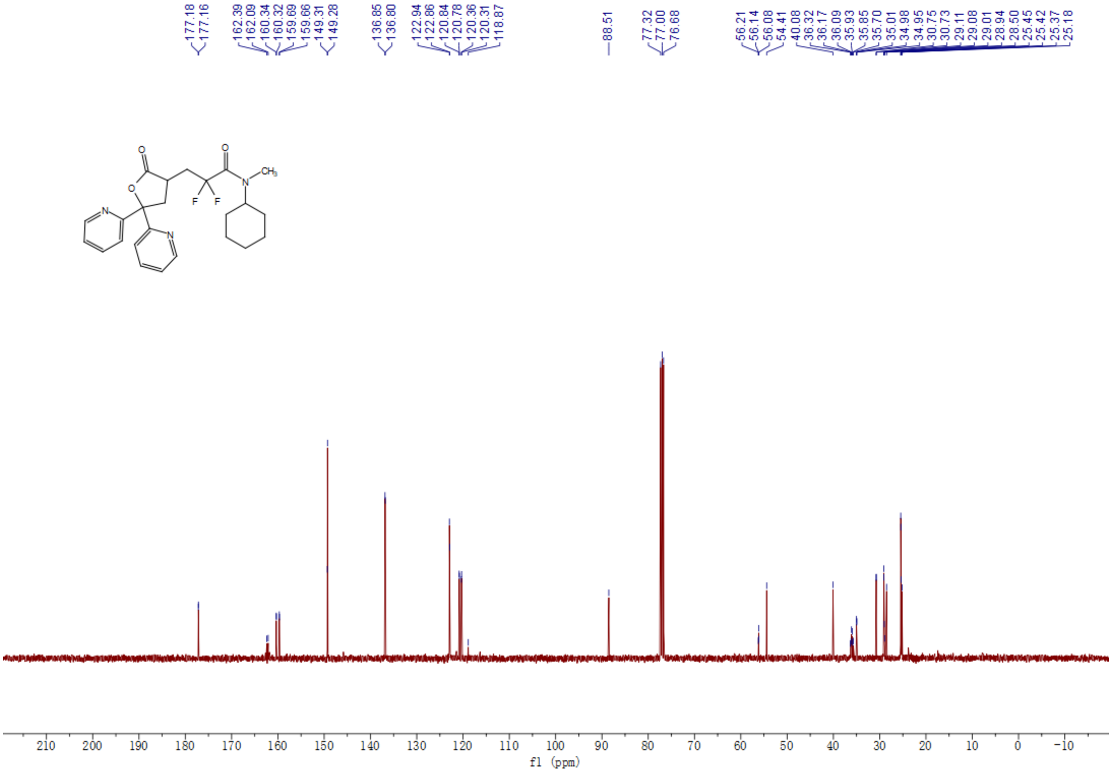

^{19}F NMR spectrum of **3an** in $CDCl_3$ (376 MHz)

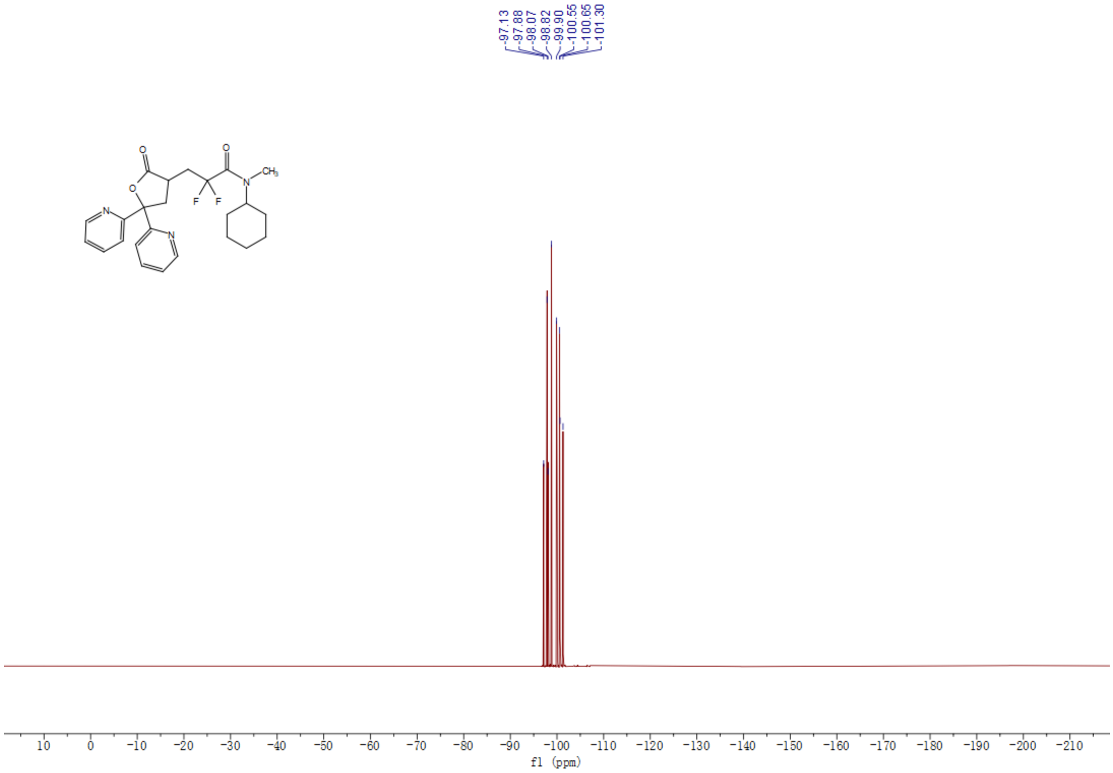

^{1}H NMR spectrum of **3ao** in $CDCl_3$ (400 MHz)

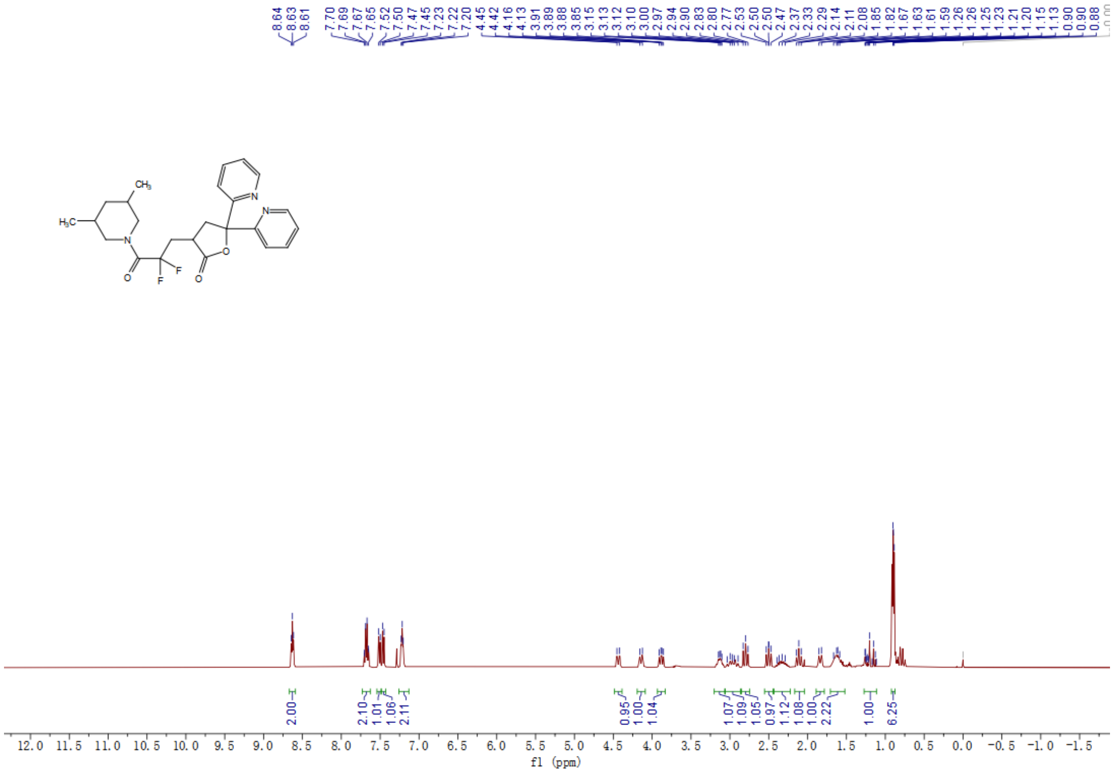

^{13}C NMR spectrum of **3ao** in $CDCl_3$ (101 MHz)

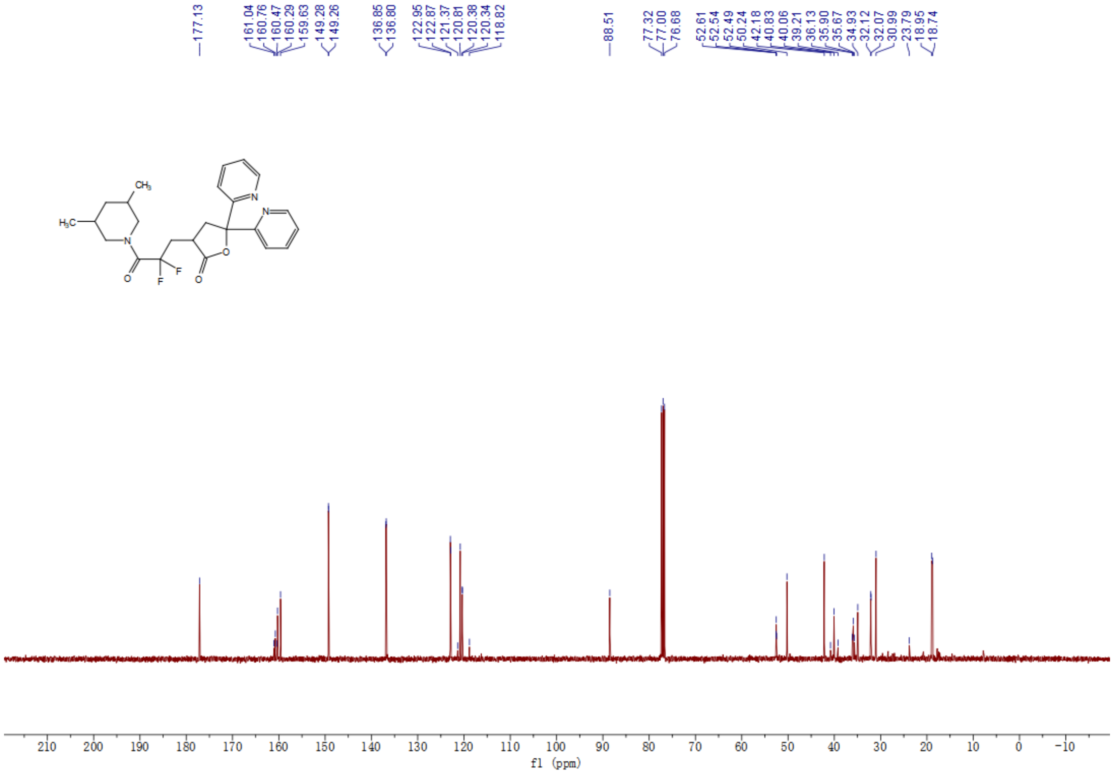

^{19}F NMR spectrum of **3ao** in $CDCl_3$ (376 MHz)

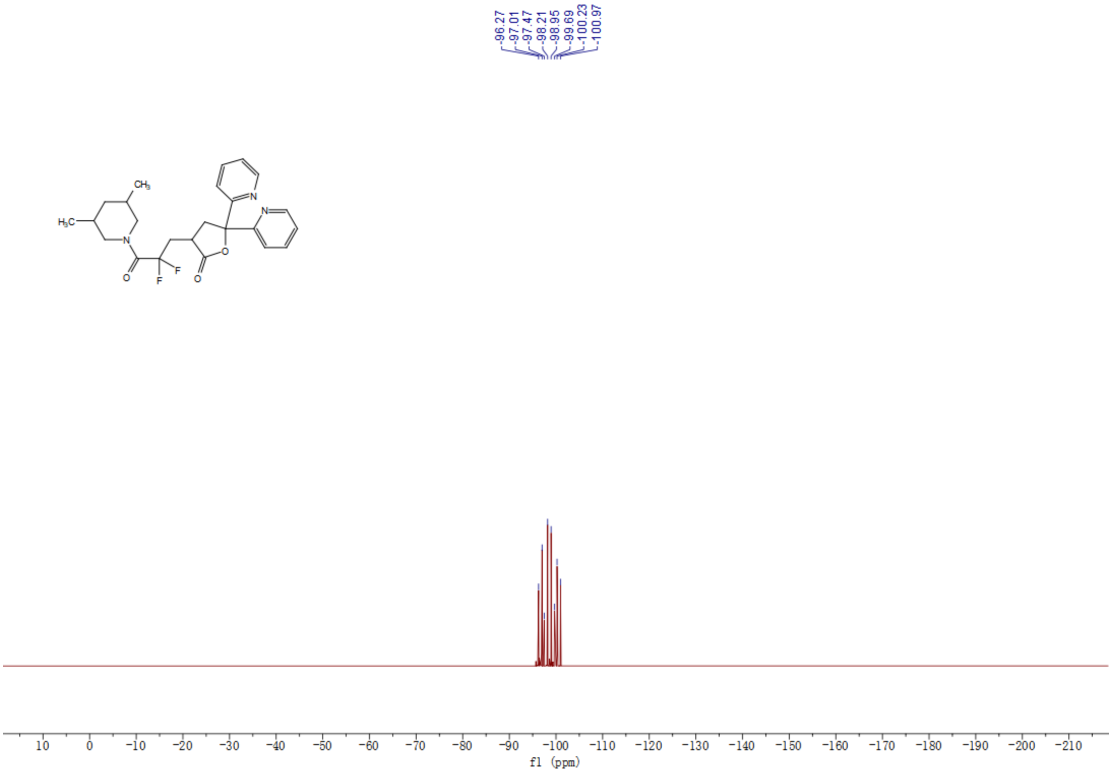

^{1}H NMR spectrum of **3ap** in $CDCl_3$ (400 MHz)

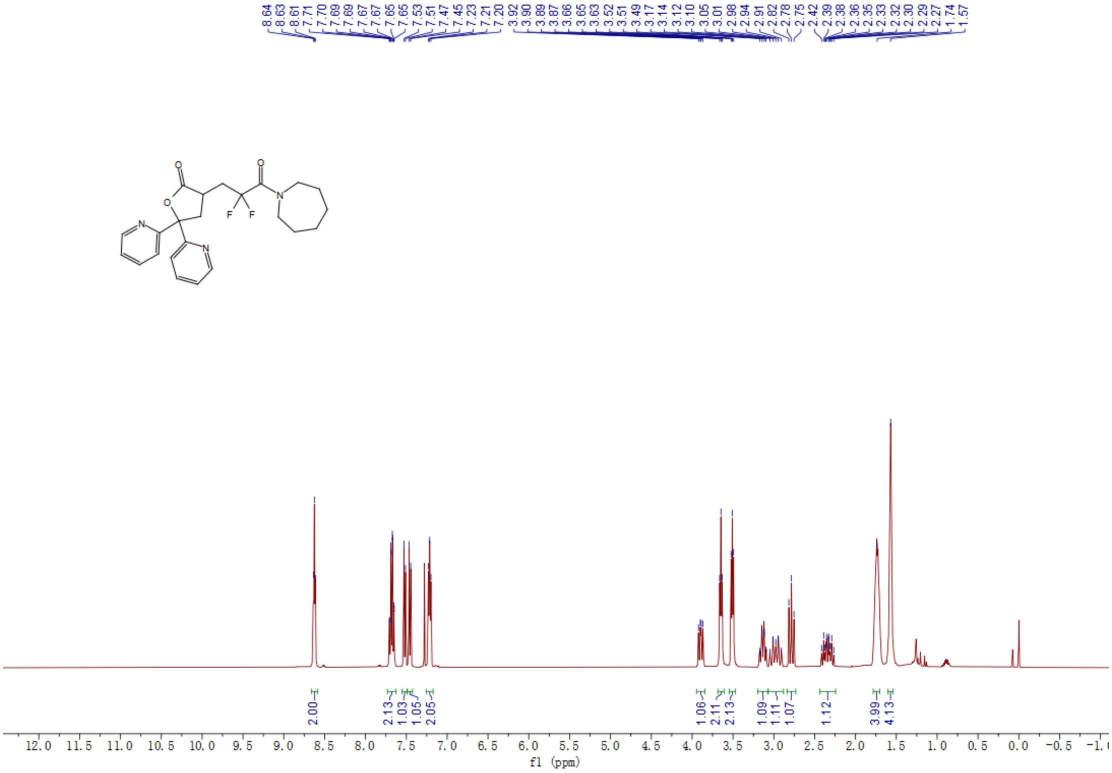

^{13}C NMR spectrum of **3ap** in $CDCl_3$ (101 MHz)

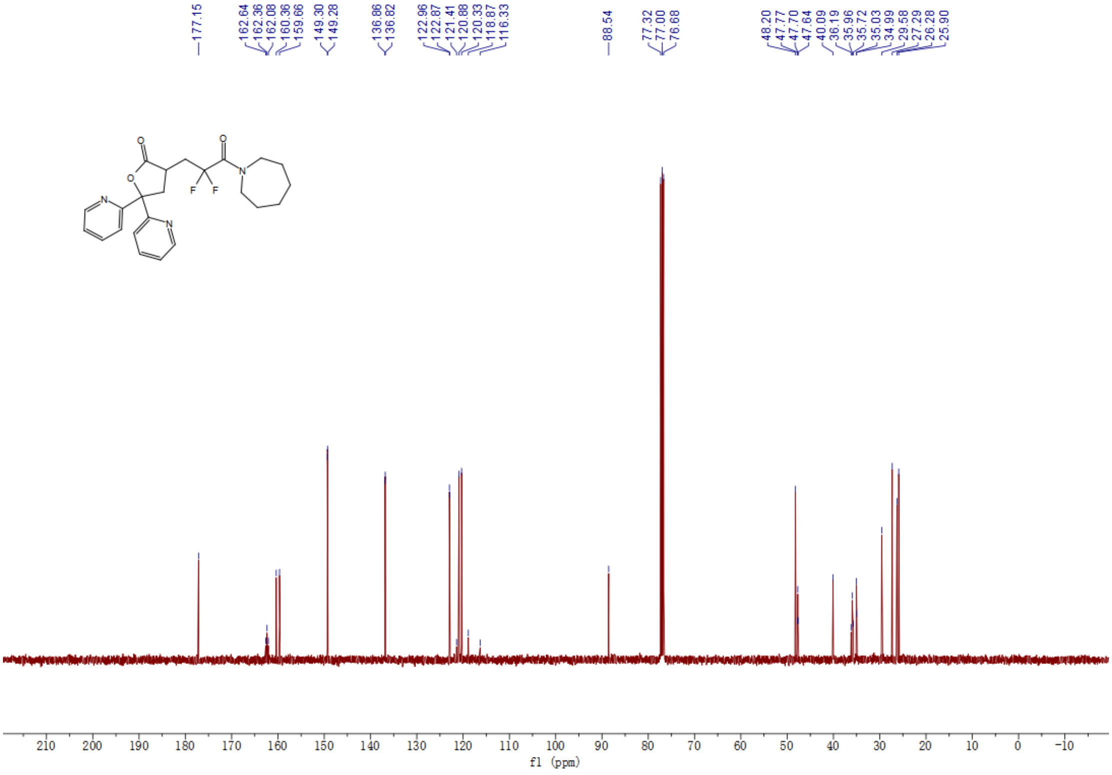

^{19}F NMR spectrum of **3ap** in $CDCl_3$ (376 MHz)

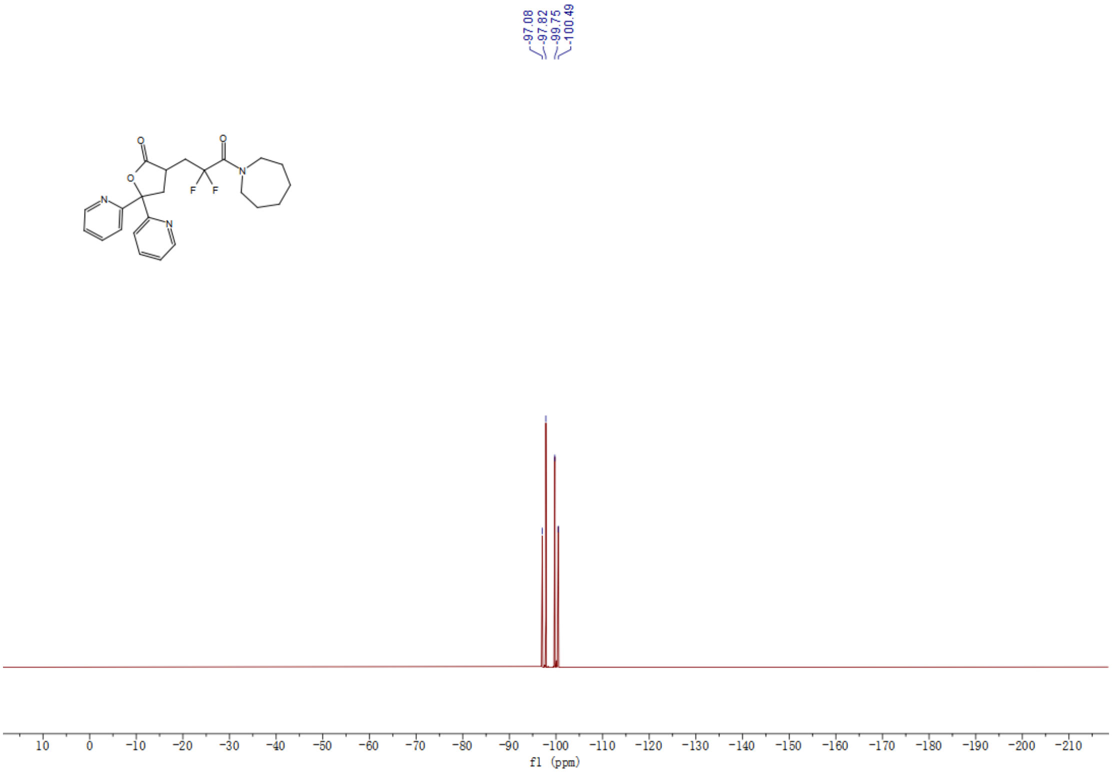

^{1}H NMR spectrum of **3ba** in $CDCl_3$ (400 MHz)

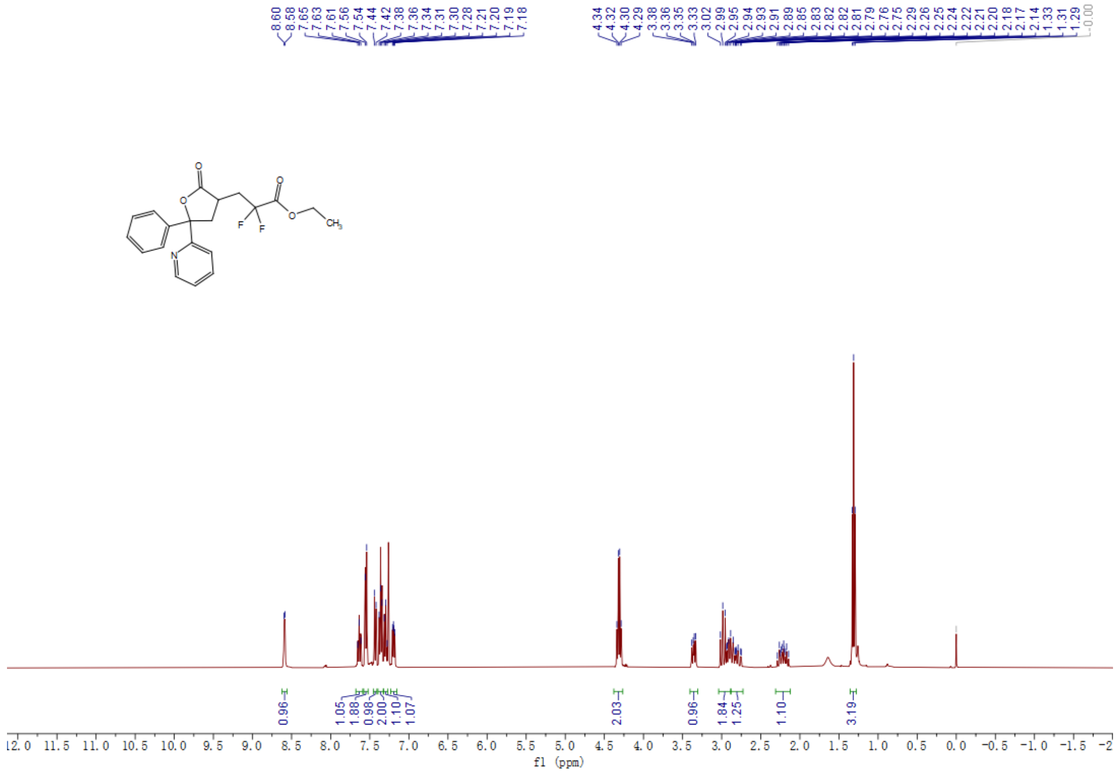

^{13}C NMR spectrum of **3ba** in $CDCl_3$ (101 MHz)

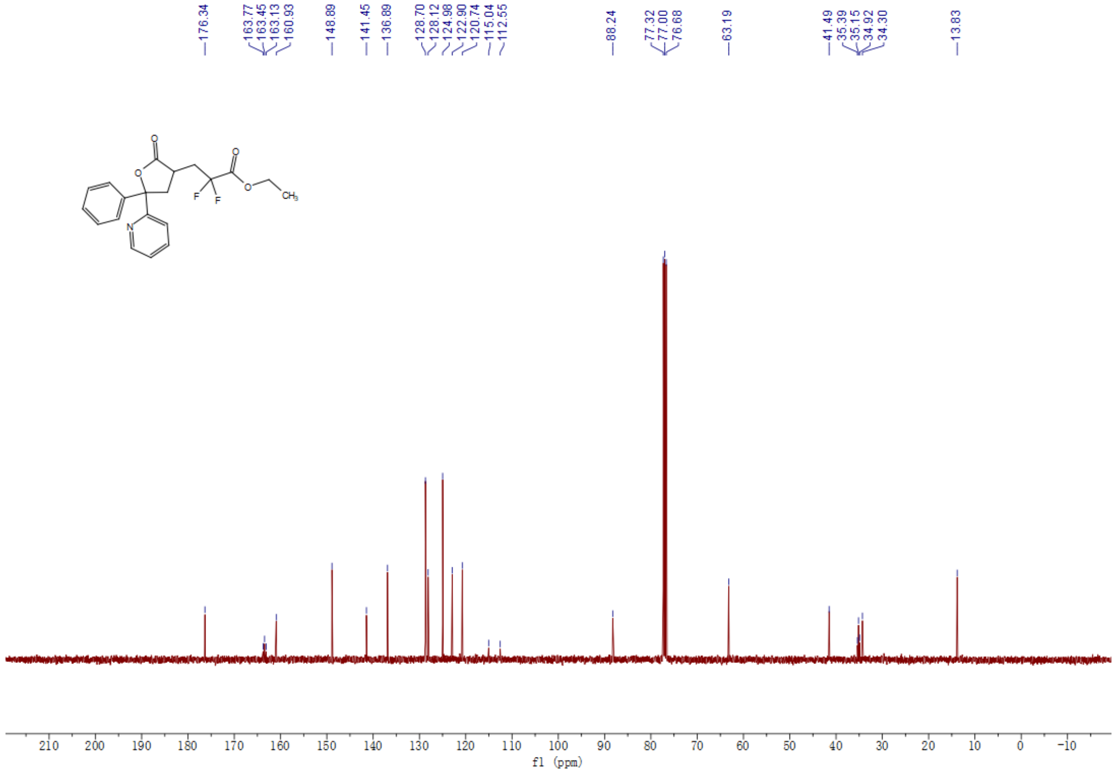

^{19}F NMR spectrum of **3ba** in $CDCl_3$ (376 MHz)

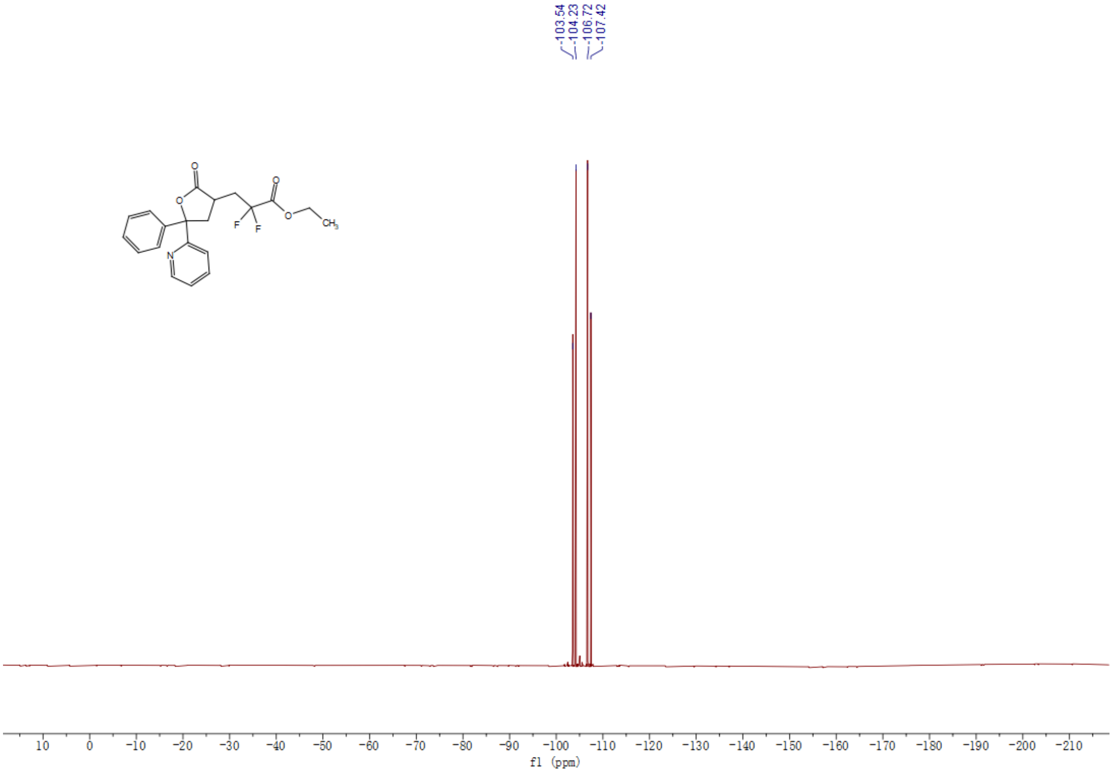

^{1}H NMR spectrum of **3ca** in $CDCl_3$ (400 MHz)

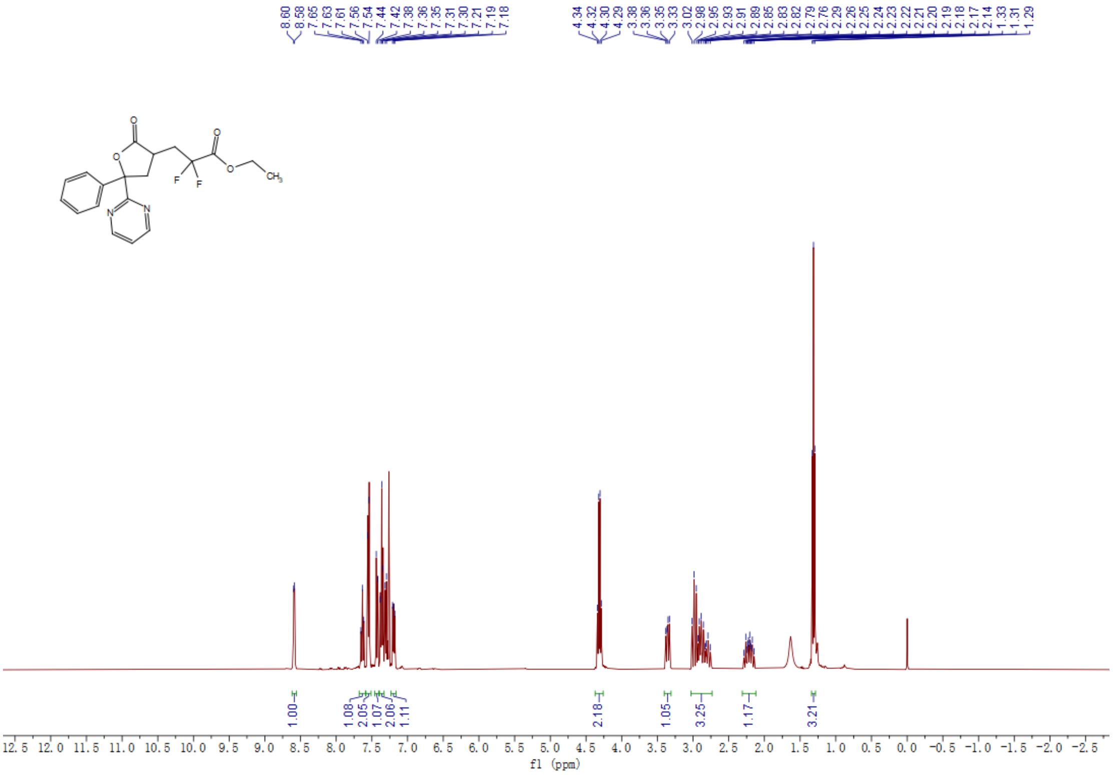

^{13}C NMR spectrum of **3ca** in $CDCl_3$ (101 MHz)

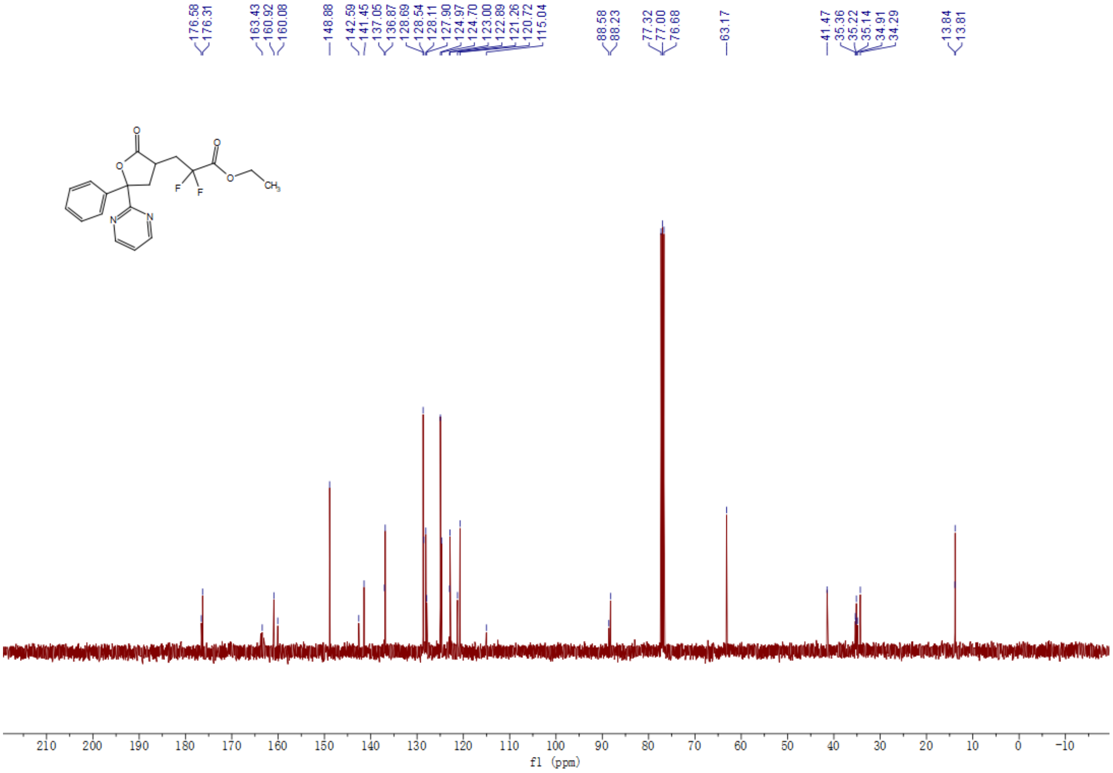

^{19}F NMR spectrum of **3ca** in $CDCl_3$ (376 MHz)

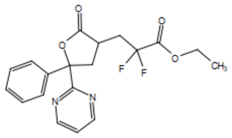

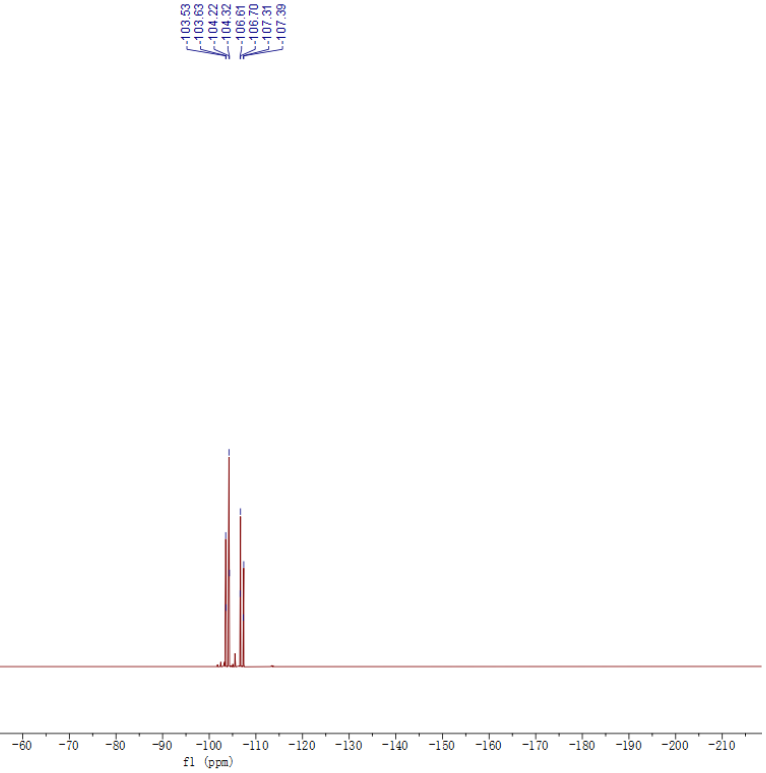

^{1}H NMR spectrum of **3da** in $CDCl_3$ (400 MHz)

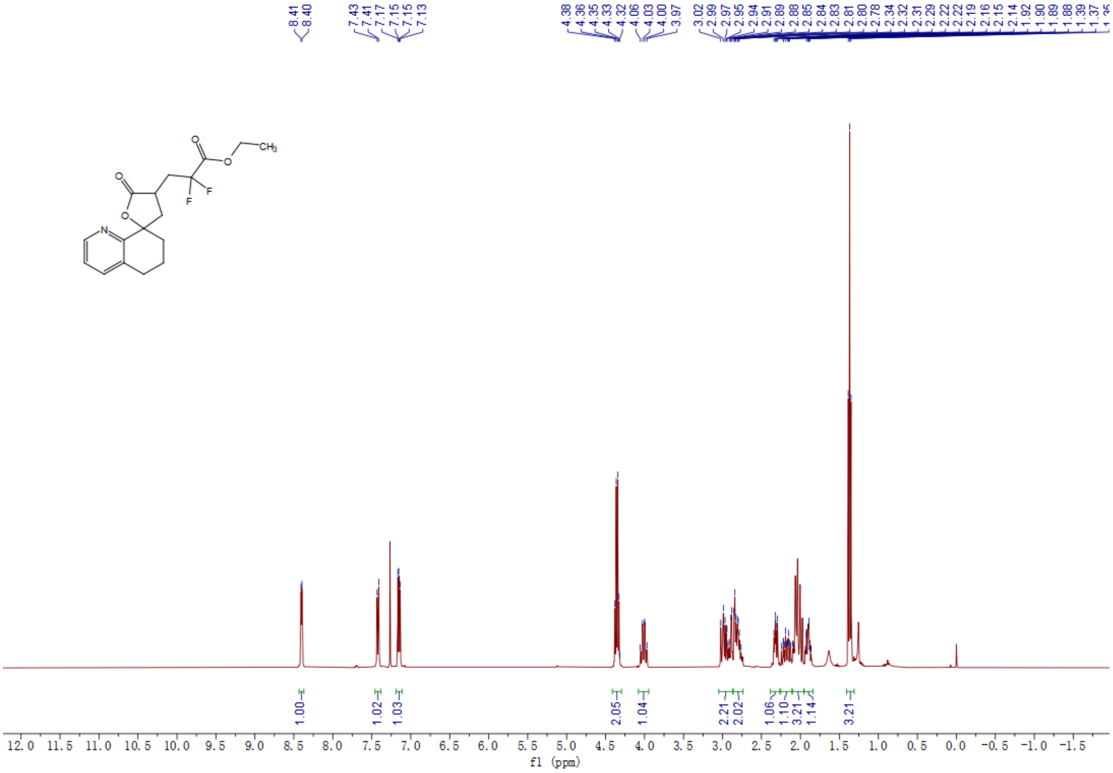

^{13}C NMR spectrum of **3da** in $CDCl_3$ (101 MHz)

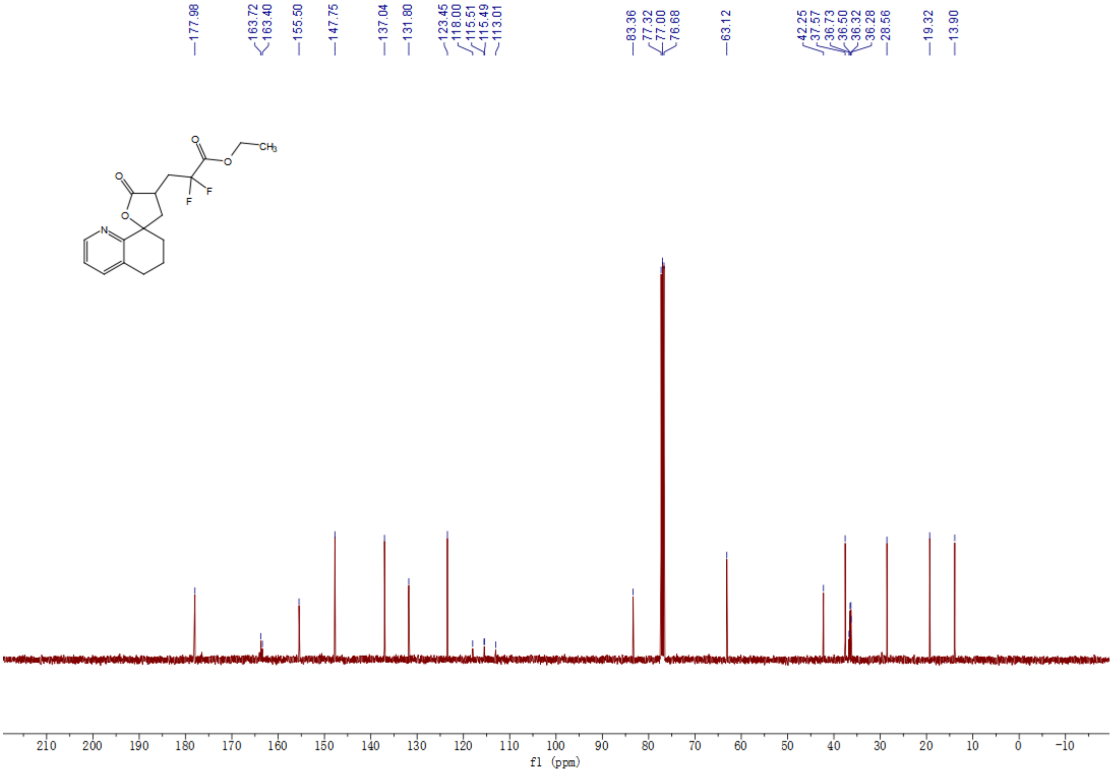

^{19}F NMR spectrum of **3da** in $CDCl_3$ (376 MHz)

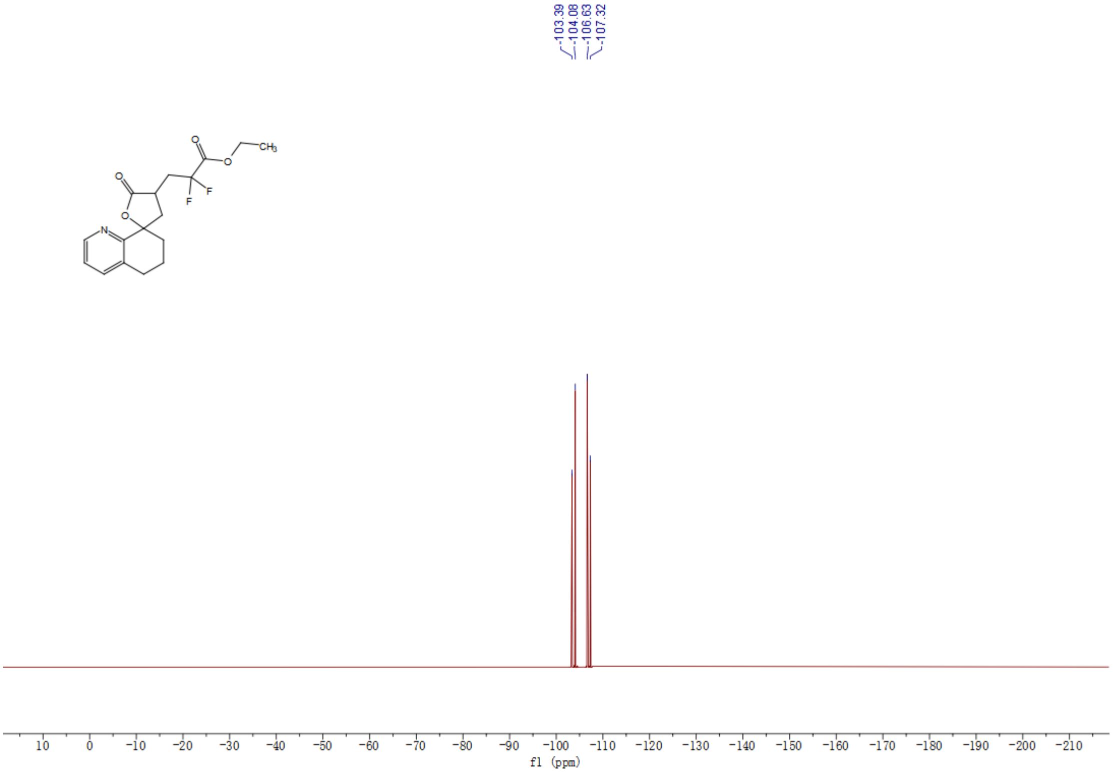

^{1}H NMR spectrum of 3ga in $CDCl_3$ (400 MHz)

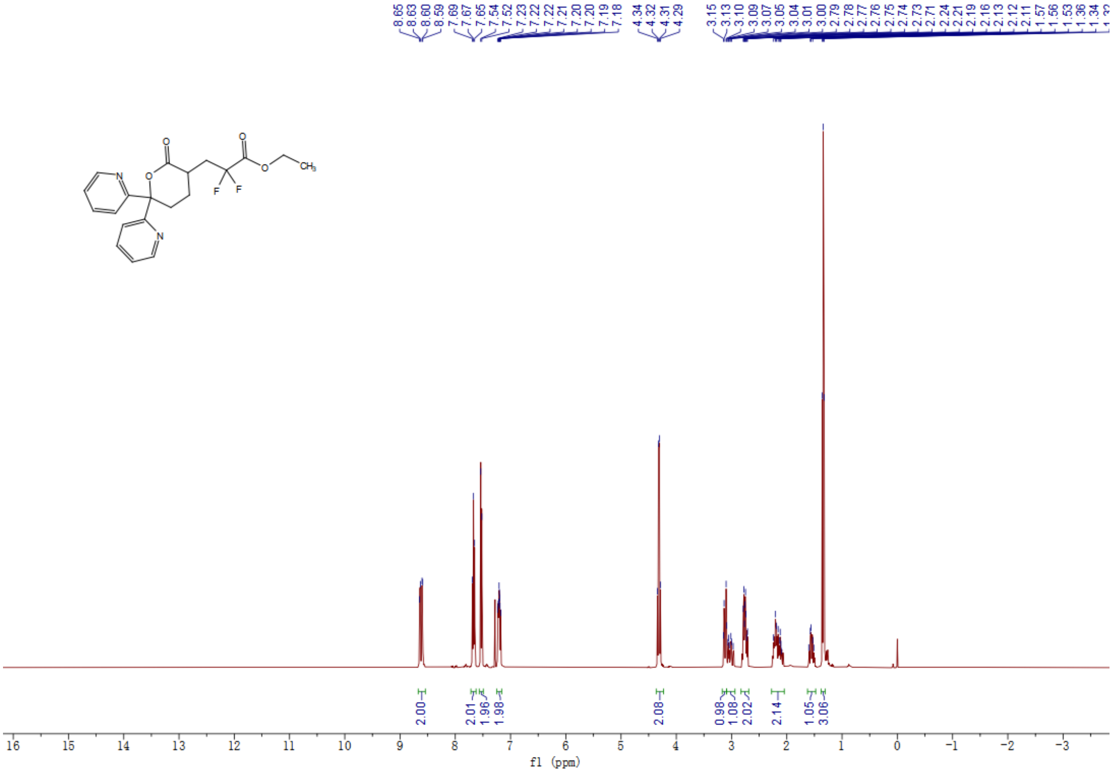

^{13}C NMR spectrum of **3ga** in $CDCl_3$ (101 MHz)

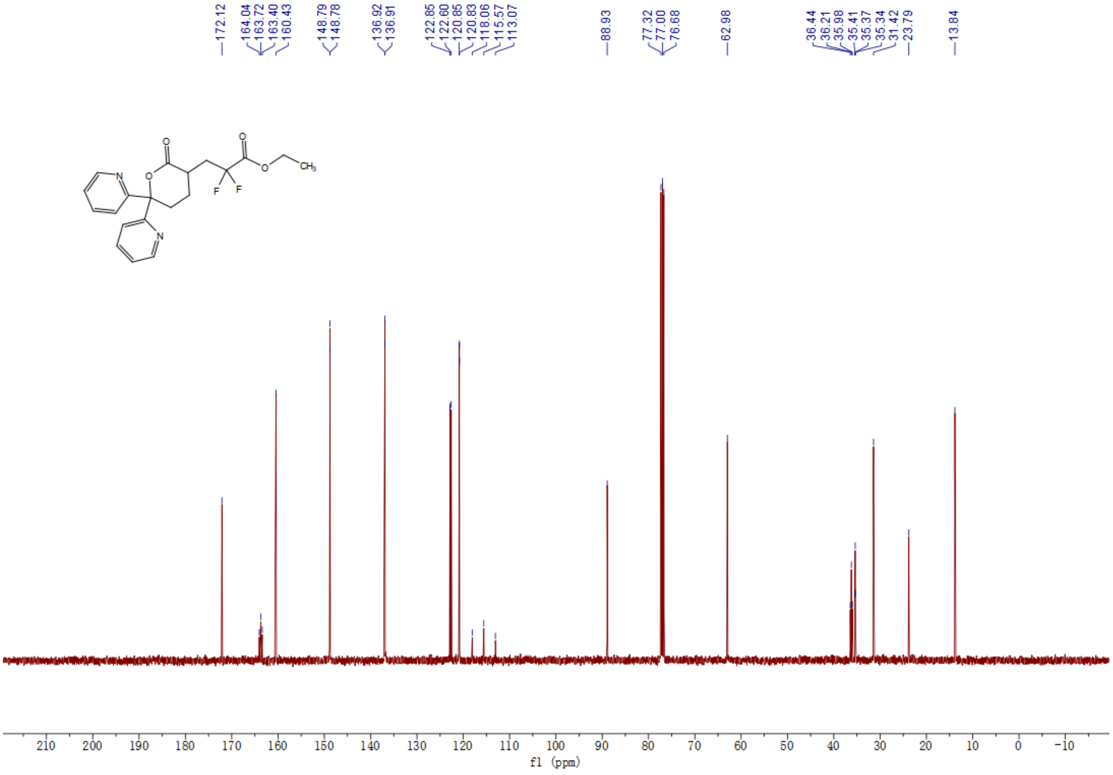

^{19}F NMR spectrum of **3ga** in $CDCl_3$ (376 MHz)

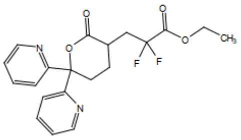

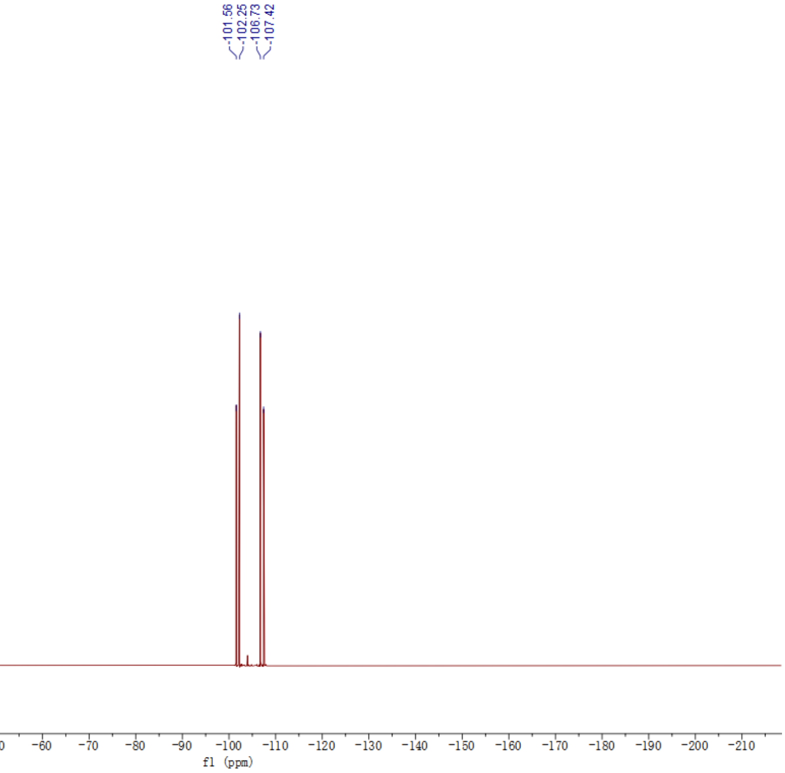

^{1}H NMR spectrum of **3gp** in $CDCl_3$ (400 MHz)

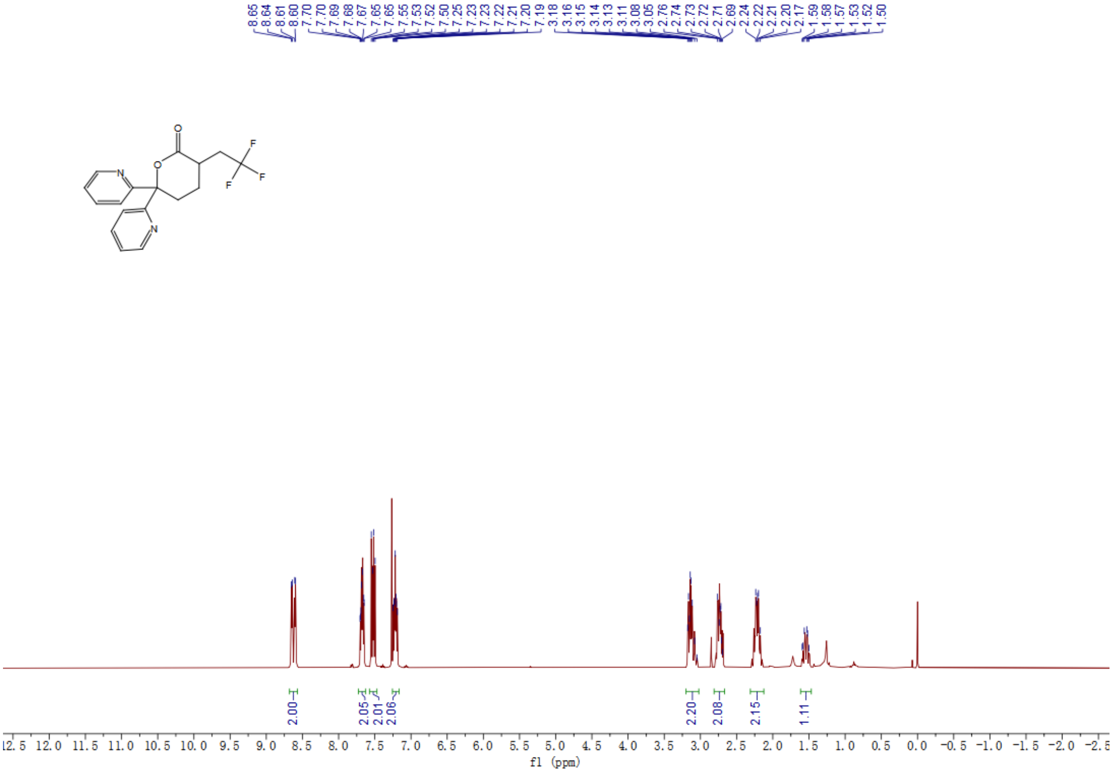

^{13}C NMR spectrum of **3gp** in $CDCl_3$ (101 MHz)

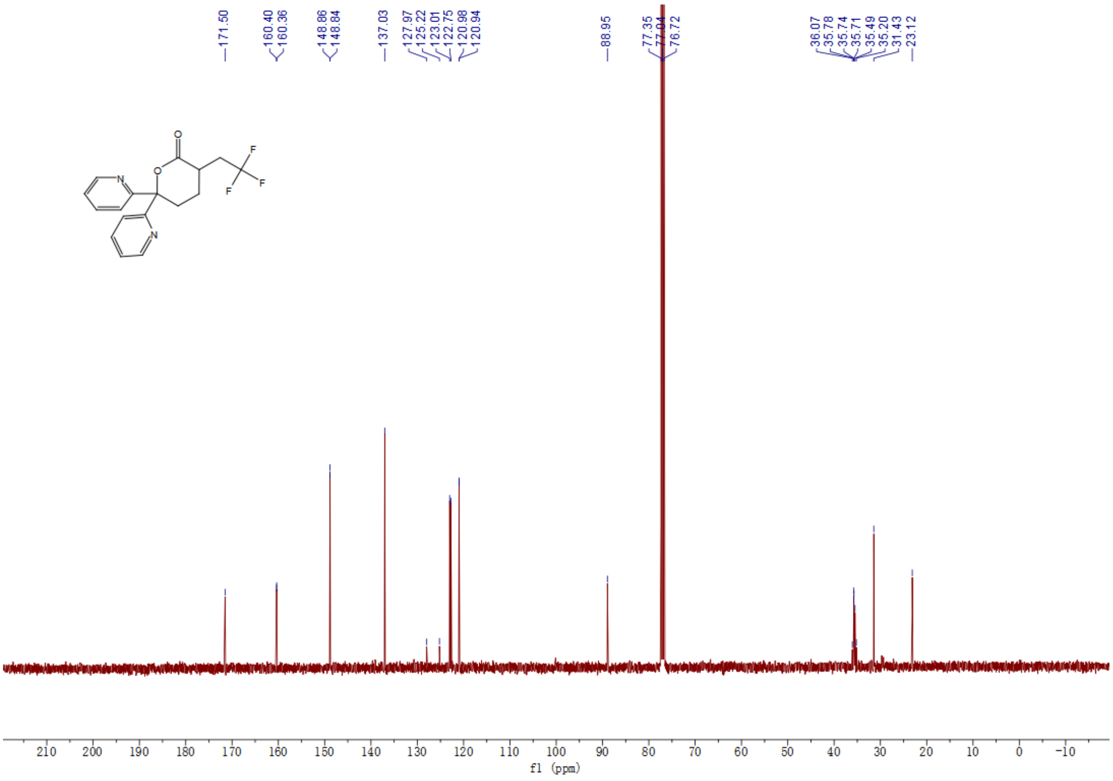

^{19}F NMR spectrum of **3gp** in $CDCl_3$ (376 MHz)

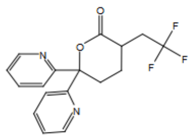

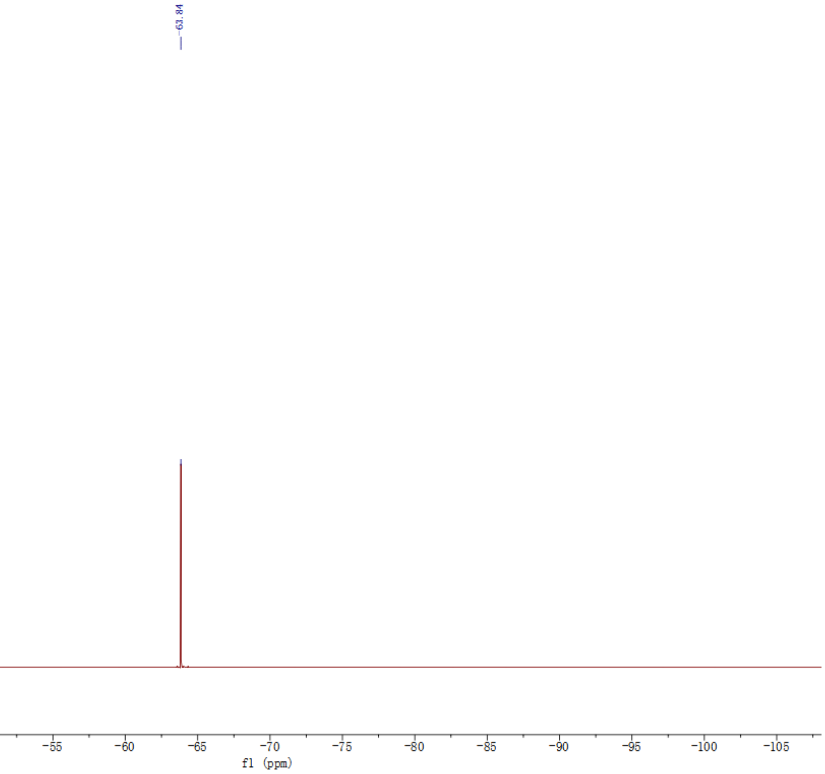

^{1}H NMR spectrum of **3ha** in $CDCl_3$ (400 MHz)

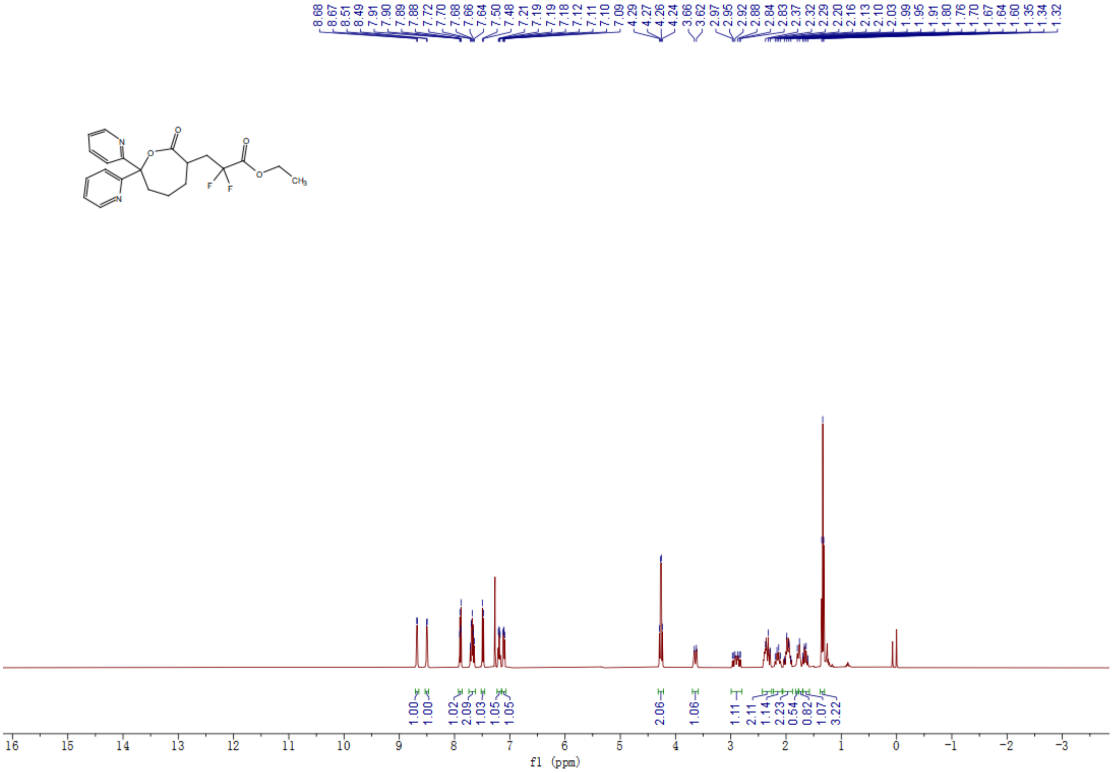

^{13}C NMR spectrum of **3ha** in $CDCl_3$ (101 MHz)

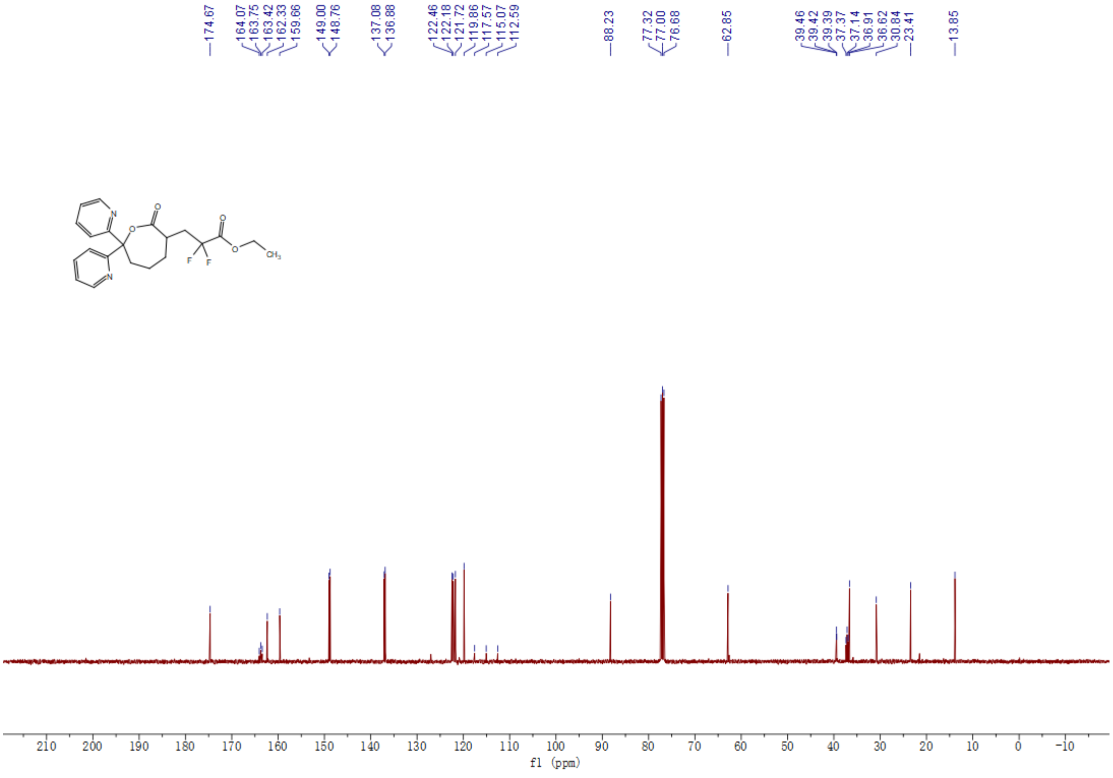

^{19}F NMR spectrum of **3ha** in $CDCl_3$ (376 MHz)

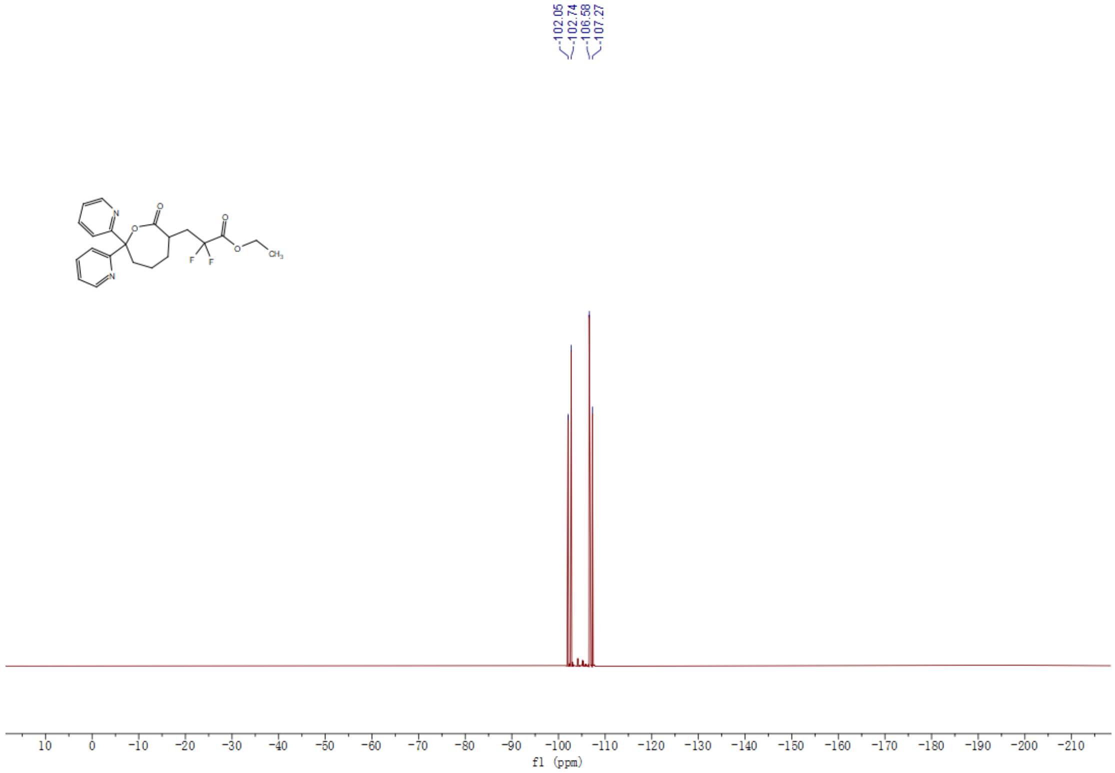

^{1}H NMR spectrum of **3aq** in $CDCl_3$ (400 MHz)

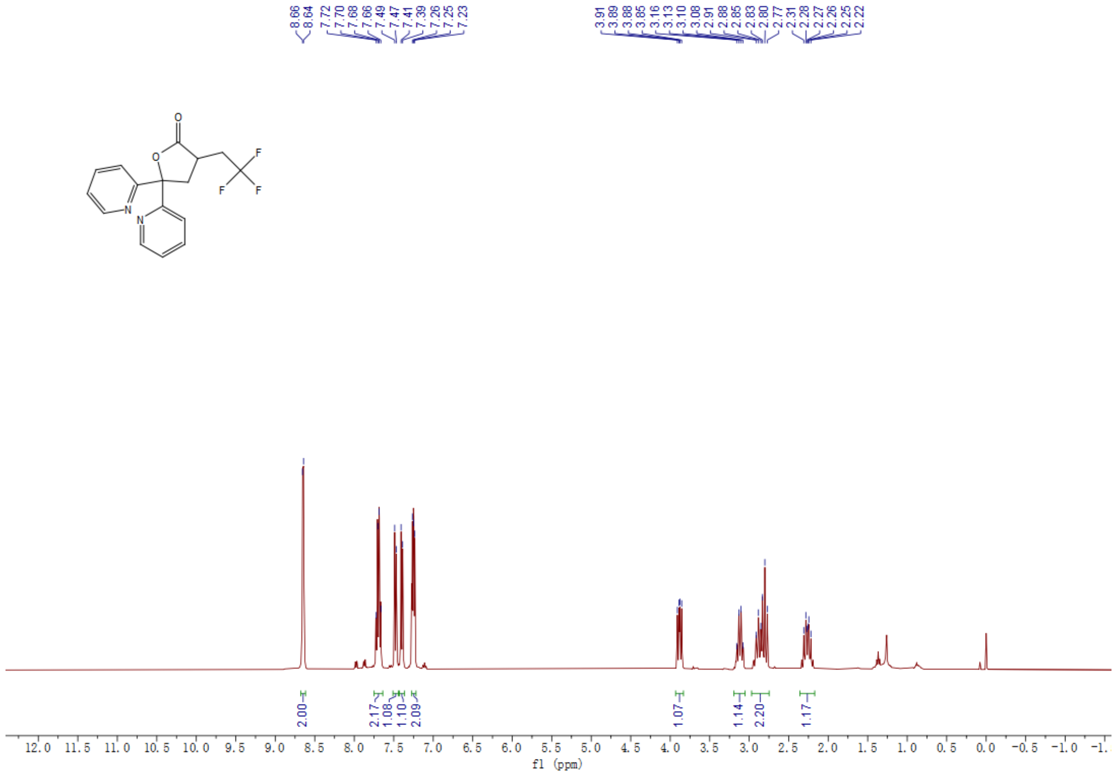

^{13}C NMR spectrum of **3aq** in $CDCl_3$ (101 MHz)

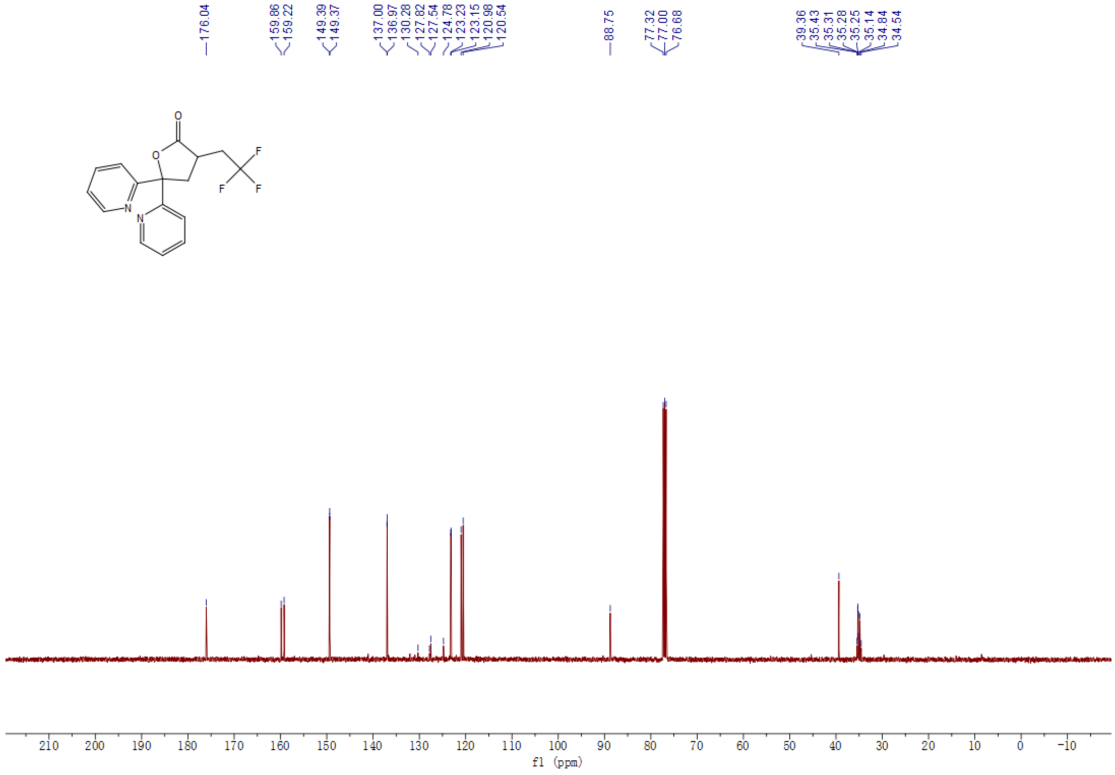

^{19}F NMR spectrum of **3aq** in $CDCl_3$ (376 MHz)

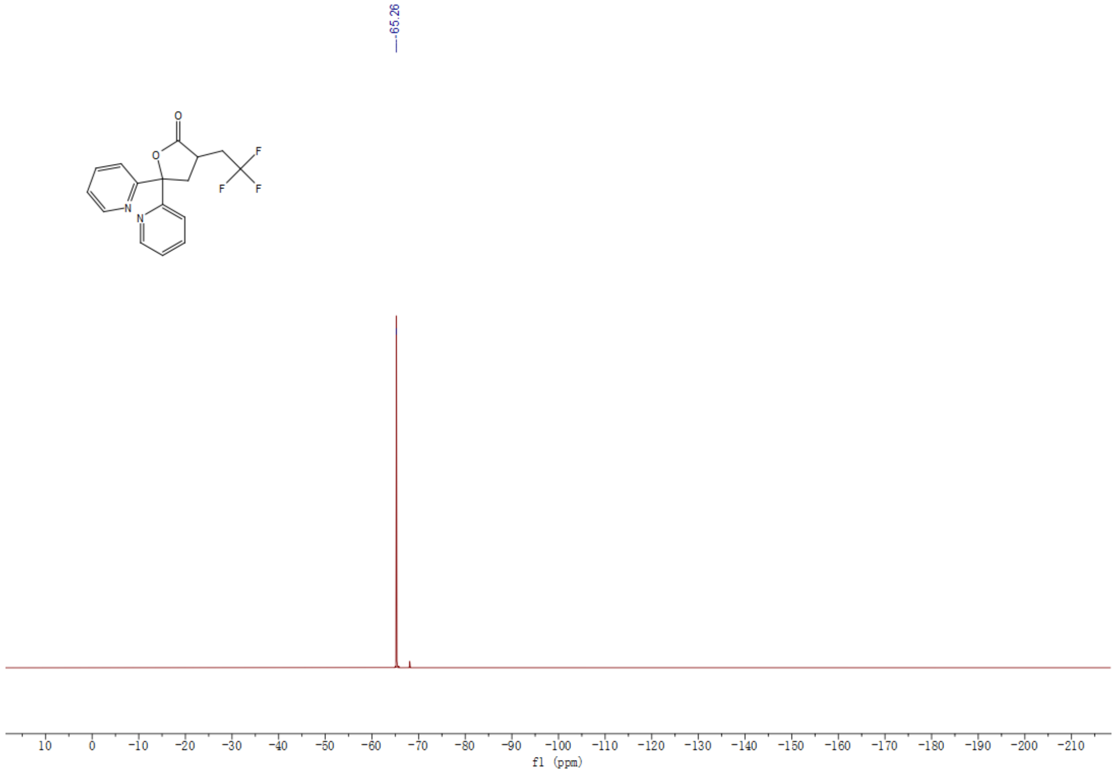

^{1}H NMR spectrum of **3ar** in $CDCl_3$ (400 MHz)

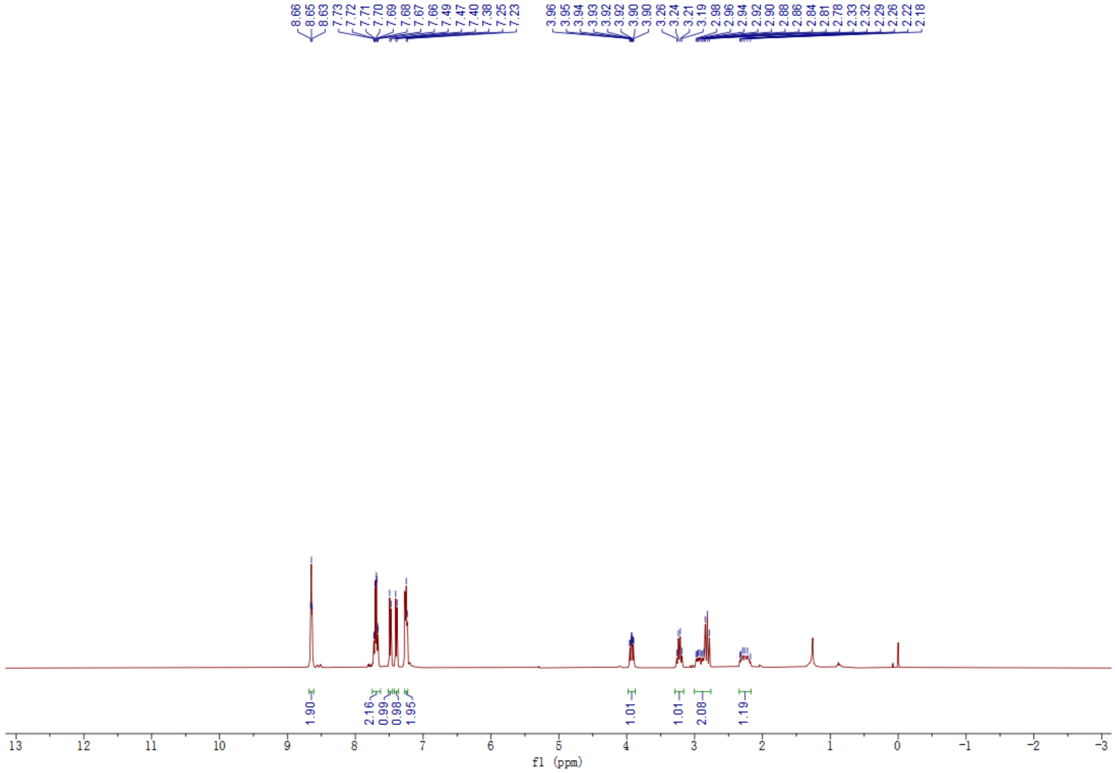

^{13}C NMR spectrum of **3ar** in $CDCl_3$ (101 MHz)

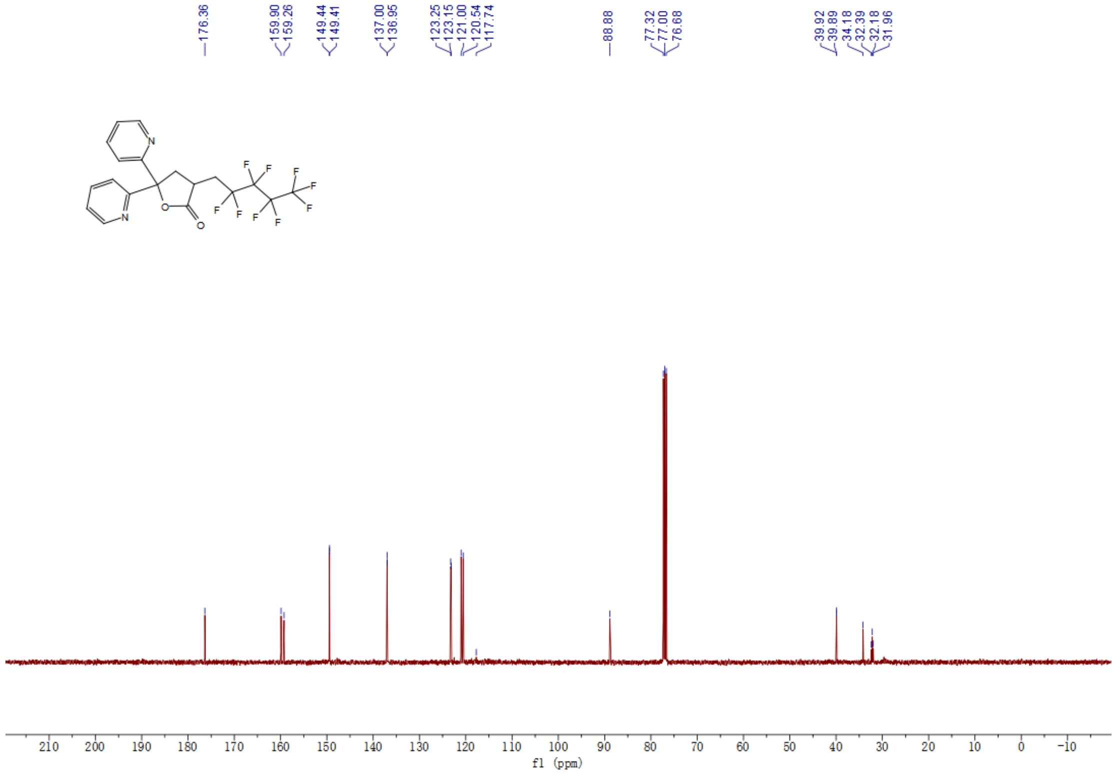

^{19}F NMR spectrum of **3ar** in $CDCl_3$ (376 MHz)

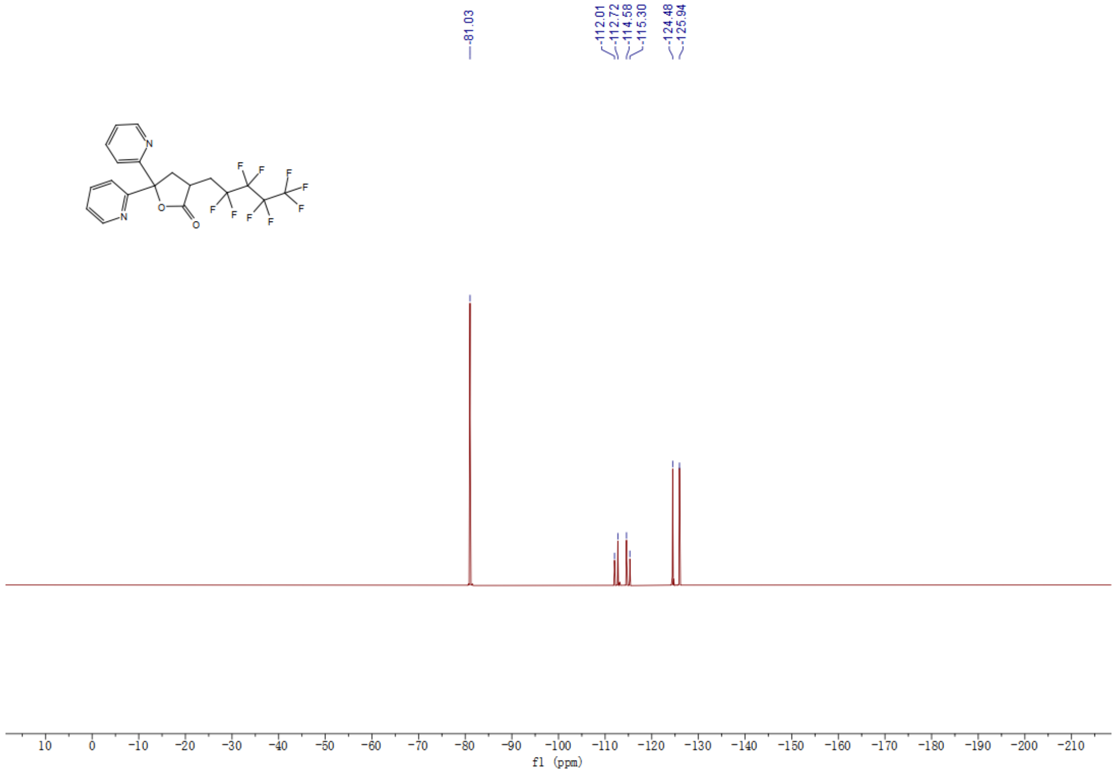

^{1}H NMR spectrum of **3as** in $CDCl_3$ (400 MHz)

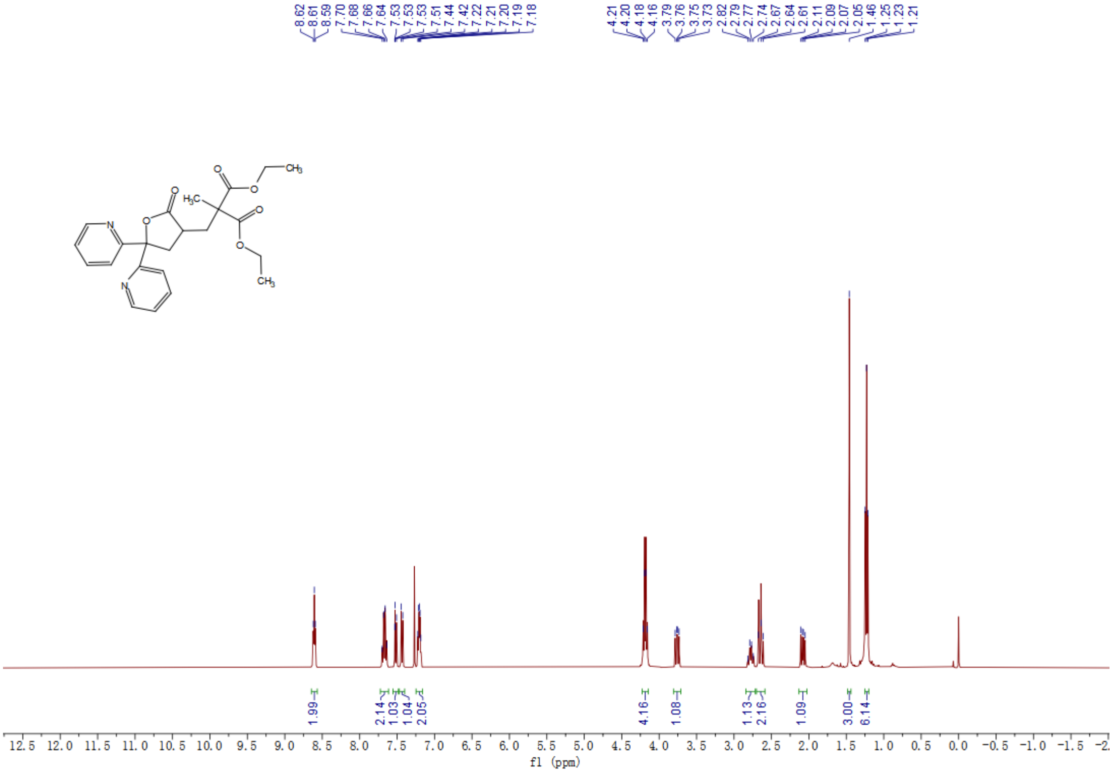

^{13}C NMR spectrum of **3as** in $CDCl_3$ (101 MHz)

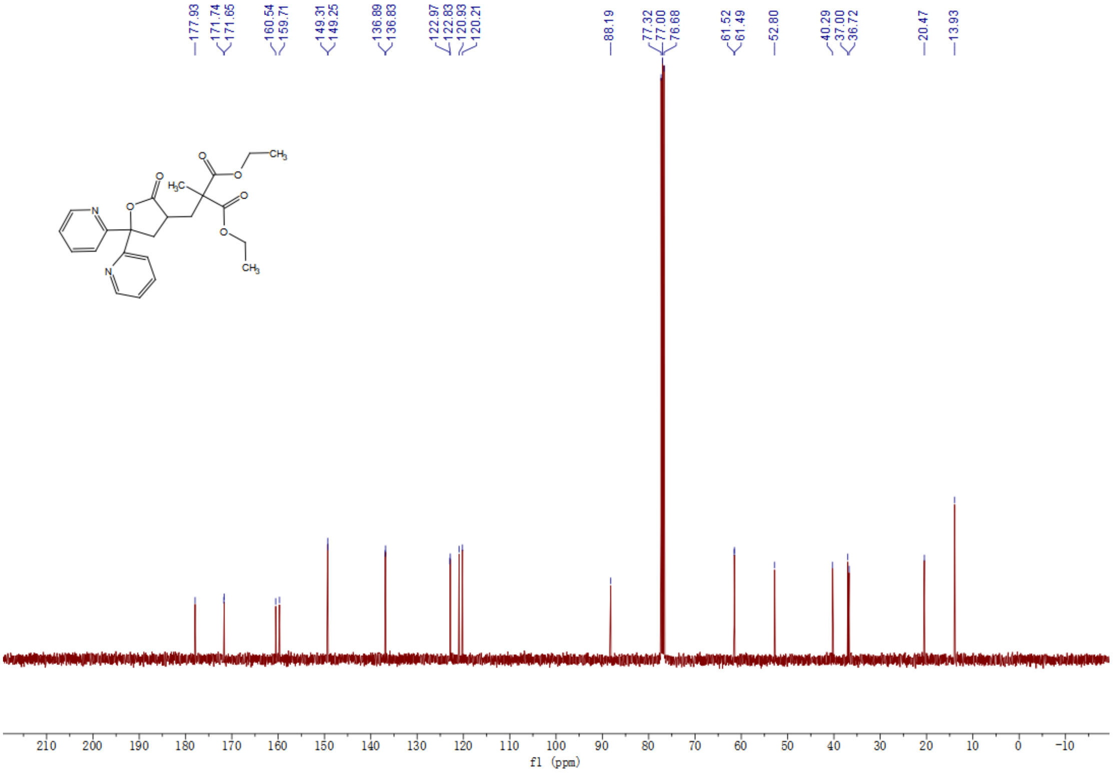

7 Reference

1.Y. Wang, H. Yang, Y. Zheng, M. Hu, J. Zhu, Z.-P. Bao, Y. Zhao and X.-F. Wu, *Nat. Catal.*, 2024, **7**, 1065-1075.

2.Z.-P. Bao, Y. Zhang and X.-F. Wu, *Chem. Sci.*, 2022, **13**, 9387-9391.

3.C. Chen, R. Zeng, J. Zhang and Y. Zhao, *Eur. J. Org. Chem.*, 2017, **2017**, 6947-6950.
